# Carboxylate Catalysis: A Catalytic *O*-Silylative Aldol Reaction of Aldehydes and Ethyl Diazoacetate

**DOI:** 10.1021/acs.joc.3c01304

**Published:** 2023-09-28

**Authors:** Saara Riuttamäki, Anton Bannykh, Petri M. Pihko

**Affiliations:** Department of Chemistry and NanoScience Center, University of Jyväskylä, P.O.B. 35, Jyväskylä FI-40014, Finland

## Abstract

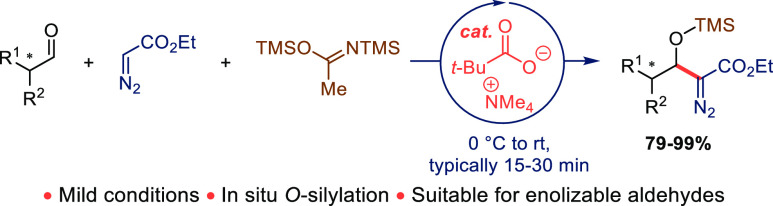

A mild catalytic variant of the aldol reaction between ethyl diazoacetate and aldehydes is described using a combination of *N*,*O*-bis(trimethylsilyl)acetamide and catalytic tetramethylammonium pivalate as catalyst. The reaction proceeds rapidly at ambient temperature to afford the *O*-silylated aldol products in good to excellent yield, and the acetamide byproducts can be removed by simple filtration.

## Introduction

Synthetic reactions involving catalytic bases are sometimes sensitive to water or unprotected hydroxy groups in the substrate, as they are rapidly deprotonated to potential nucleophiles by the catalyst. In addition, unprotected hydroxyl groups in the product could also present problems. For example, the aldol addition and related reactions give rise to unprotected aldolates, which may render the reaction reversible and result in incomplete conversions.^1^

To overcome these limitations, reactions involving catalytic bases are sometimes assisted by auxiliary hard acid reagents, such as magnesium ions or silylating agents.^2^ Herein we describe a particularly mild combination of a carboxylate salt and *N*,*O*-bis(trimethylsilyl)acetamide (BSA)^3^ as a catalyst/silyl reagent combination, as traces of reagent and catalyst can be removed by simple trituration, filtration, and concentration.

**Scheme 1:**
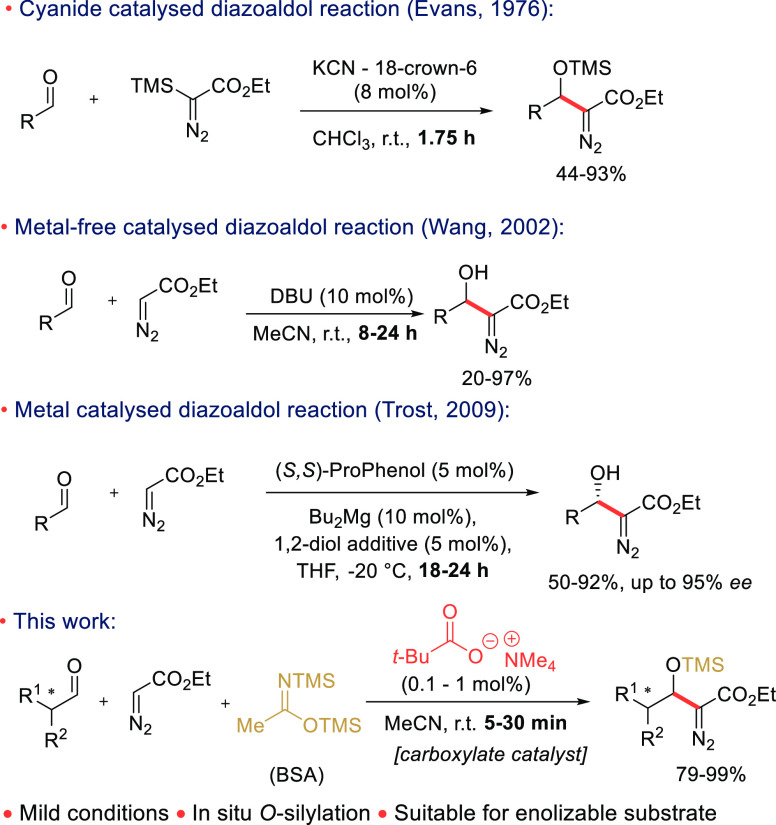
Examples of Aldol-Type Reactions between Ethyl Diazoacetates and Aldehydes

We have selected the aldol reaction between ethyl diazoacetate and aldehydes to illustrate the benefits of the catalytic system. The first published variant of the reaction, catalyzed by KOH in methanol, resulted in an equilibrium that favored the starting materials.^4^ To overcome this limitation, milder catalytic methods and other variants have been published. In 1976, Evans and co-workers described a mild *O*-silylative variant which required the use of TMS-activated diazoacetate and catalytic KCN/18-crown-6.^5^ Even milder methods employing, among others, DBU,^6^ quaternary ammonium hydroxide,^7^ mixed La_2_O_3_/MgO,^8^ or benzoic acid^9^ as catalysts have been disclosed. With metal phenolates or organometallic bases, the reaction has also been rendered enantioselective^10^ (Scheme 1).

Typically, the reactions have been restricted to simple aryl or alkyl aldehydes, and chromatographic purification has been required for the products. On the basis of our previous work on carboxylate catalysis in the enolization of thioesters with simple carboxylate catalysts such as tetramethylammonium pivalate (TMAP),^11^ we hypothesized that the diazoacetate aldol reaction should also be amenable to carboxylate catalysis. A few examples of catalytic systems involving carboxylate catalysts have been published, demonstrating diverse applications in C–C bond formation as well as in proton-transfer reactions.^12^ Herein we describe a particularly mild, catalytic method for the one-pot silylative diazoacetate aldol using a mild silylating agent (*N*,*O*-bis(trimethylsilyl)acetamide, BSA) that gives the *O*-silylated aldol products in good yields even without any chromatographic purification.

## Results and Discussion

Tetramethylammonium pivalate (TMAP, **3**) was chosen as the carboxylate catalyst for our test reactions with benzaldehyde **1a** and ethyl diazoacetate **2** since TMAP is soluble in acetonitrile. With a catalytic amount of TMAP (Table 1, entry 1), only traces alcohol **4a** could be obtained. We hypothesized that the poor turnover in this experiment resulted from deactivation of the catalyst by proton transfer from **4a**. Indeed, with a stoichiometric amount (1.2 equiv) of TMAP, the reaction reached 41% conversion (Table 1, entry 2). To overcome this problem, a silylating agent, *N*,*O*-bis(trimethylsilyl)acetamide (BSA), was added to the reaction (entry 3), this resulted in full conversion to *O*-silylated product **5a** in less than 3 h. Encouraged by this success, we explored the conditions further on a preparative scale (Table 1, entries 4–8).

**Table 1:**
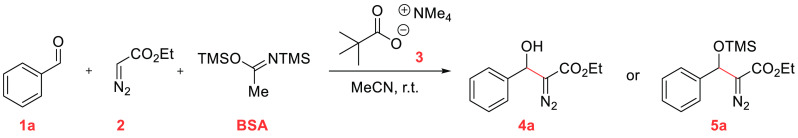
Optimization of the TMAP-Catalyzed Diazoaldol-Type Reaction

entry	**1a** (mmol/M)	BSA (equiv)	TMAP (equiv)	reaction time	yield (%)/product^a^
1	0.04/0.07	–	0.1	1 h	3^a^/**4a**
2	0.04/0.07	–	1.2	30 min	41^a^/**4a**
3	0.04/0.07	1.1	0.05	2.5 h	100^a^/**5a**
4	0.98/0.07	2.0	0.01	10 min	93/**5a**
5	0.98/0.15	2.0	0.1	10 min	94/**5a**
6	0.98/0.3	2.0	0.01	10 min	92/**5a**
8	0.98/1.0	2.0	0.01	5 min	96/**5a**
9	0.98/1.0	2.0	0.001	24 h	94/**5a**

a Conversion to product (determined by ^1^H NMR). Bn_2_O (1 equiv) was used as an internal standard.

**Scheme 2:**
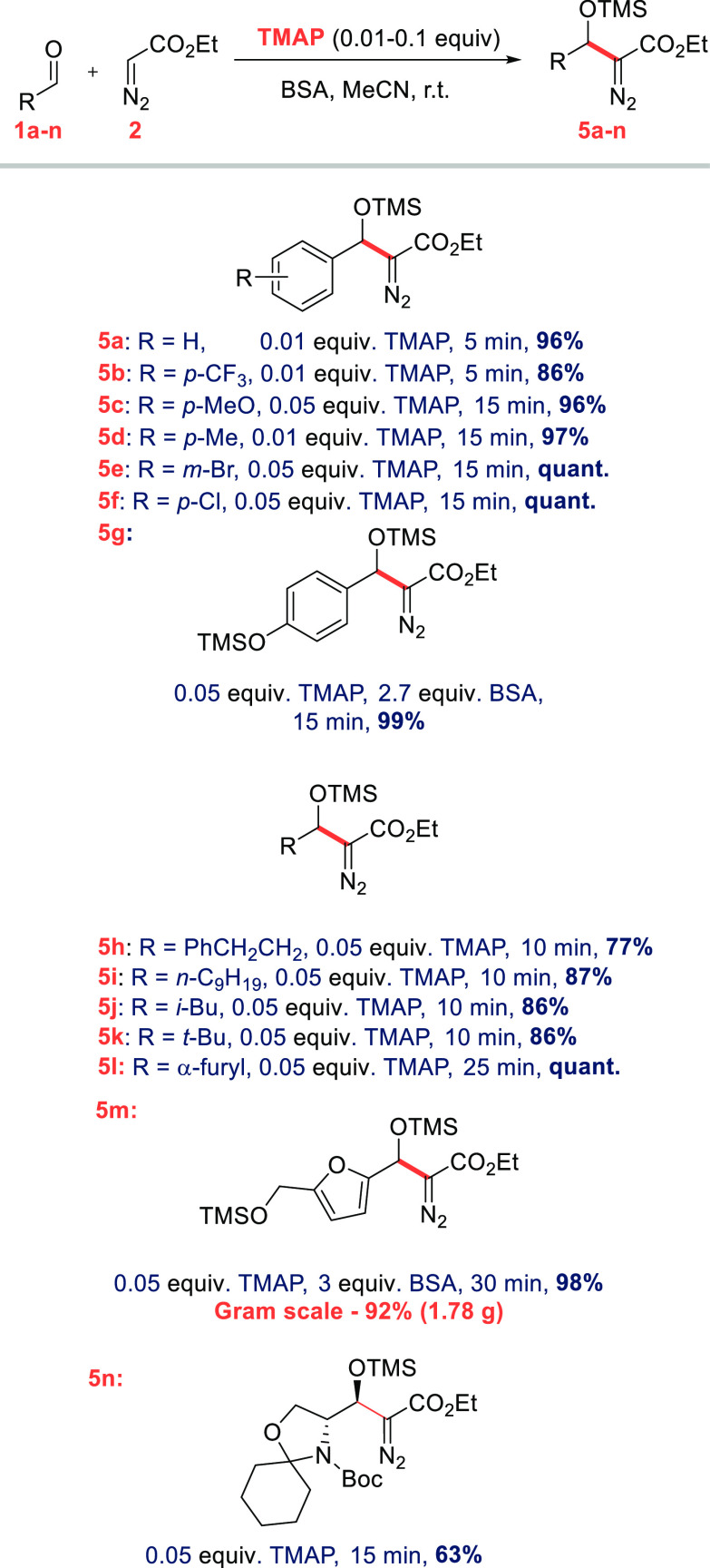
Substrate Scope of the TMAP-Catalyzed *O*-Silylative Aldol Reaction

In dry acetonitrile, the reaction was generally over in 5–10 min after adding 1–10 mol % of the catalyst (Table 1, entries 4–8). Only with very low catalyst loading (0.1 mol %, see entry 9), the reaction takes 24 h to reach completion. The reaction proved to be very high yielding, with over 90% yield in every optimization test (entries 4–9). The same theme continued in the substrate scope (Scheme 2). The reaction gives high yields with both aromatic (**5a**–**g**), aliphatic (**5h**–**m**) and with heterocyclic aldehydes (**5l**–**n**). In most cases, the products could be obtained without chromatographic purification, as the silylacetamide byproducts could be removed by simple trituration with hexanes and filtration (see Experimental Section for details). 4-Hydroxybenzaldehyde (**1g**) is also a viable substrate if it is first treated with excess of BSA to silylate the phenolic hydroxy group before adding the catalyst to the reaction mixture. The doubly *O*-silylated product **5g** was obtained in high yield (98%).

**Scheme 3:**
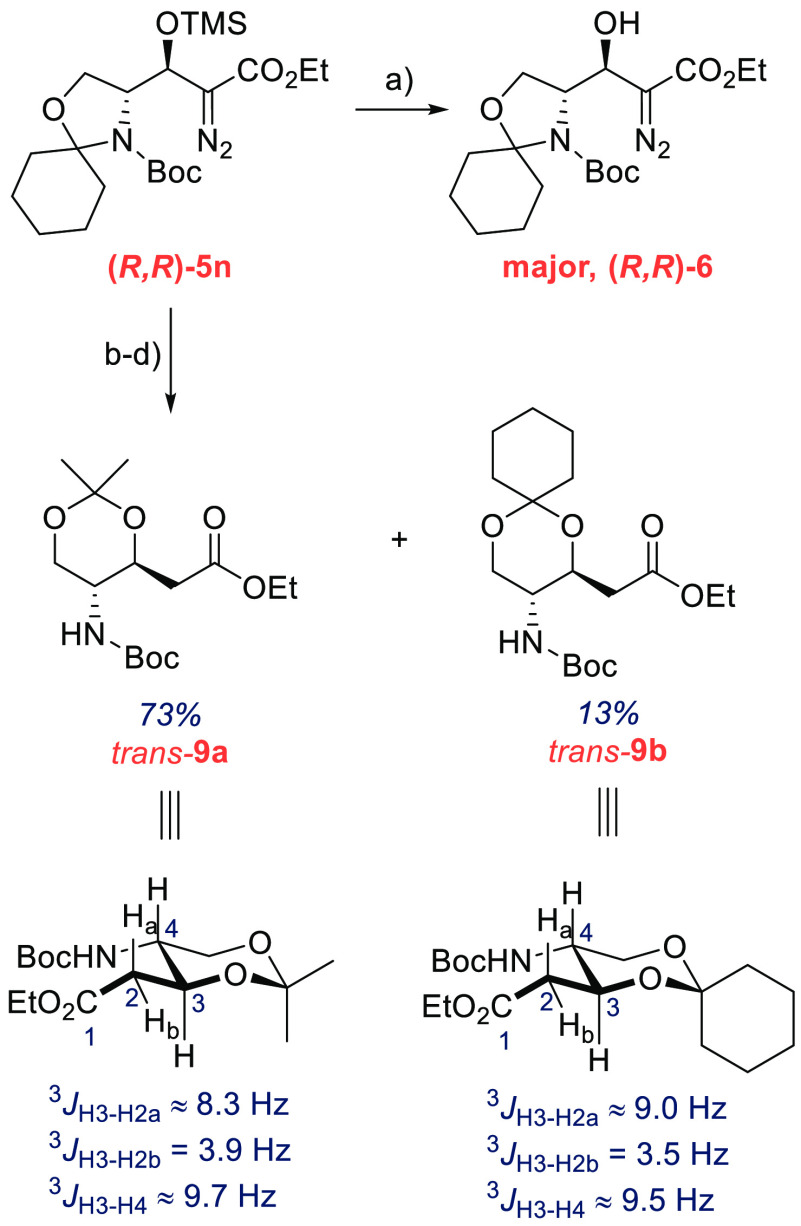
Stereochemical Assignment of **5n** Conditions: (a) 1% HCl, THF, 0 °C, 84%; (b) H_2_, PtO_2_ (10 mol %), AcOH (cat.), EtOAc, rt., 73%; (c) HF–pyridine (20 mol %), MeCN–H_2_O, 0 °C, 81%; (d) 2,2-dimethoxypropane, (+)-CSA (10 mol %), Me_2_CO, rt.

The conditions are mild enough to preserve enantiomeric purity of sensitive aldehydes, as exemplified by the synthesis of **5n** in 91:9 dr. After desilylation, the corresponding alcohol **6** shows excellent er = 97:3 (dr = 91:9) (see Scheme 3). Similar results were obtained with a reaction carried out at 0 °C. **5n** can be readily converted to the acetonide **9a**, enabling the confirmation of the stereochemistry (Scheme 3). The observed anti stereochemistry is consistent with literature precedents^9^ and the polar Felkin–Anh model.^13^

**Scheme 4:**
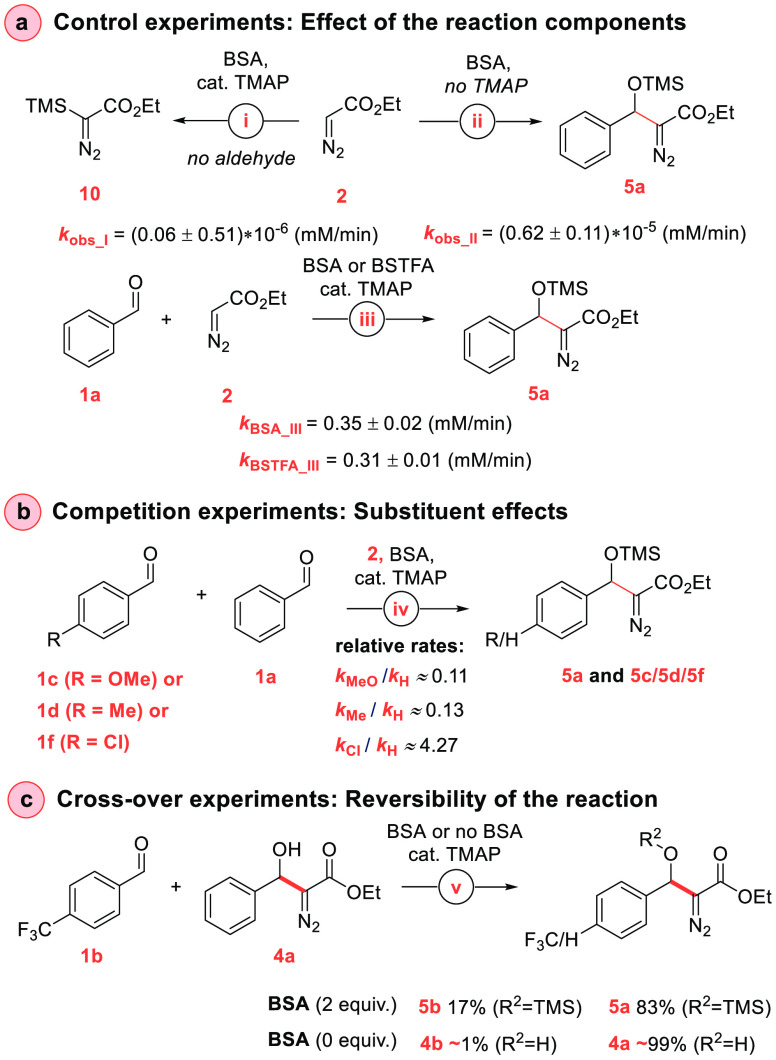
Experiments To Probe the Reaction Mechanism Reaction conditions for control experiment: (a) (i) EDA (1 equiv, 0.08 M), BSA (2 equiv), TMAP (1 mol %), Bn_2_O (1 equiv), CD_3_CN (600 μL), rt. (ii) **1a** (1 equiv, 0.06 M), EDA (1 equiv), BSA (2 equiv), Bn_2_O (1 equiv), CD_3_CN (600 μL), rt. (iii) **1a** (1 equiv, 0.07 M), EDA (1 equiv), BSA or BSTFA (2 equiv), TMAP (0.1 mol %), Bn_2_O (1 equiv), CD_3_CN (600 μL), 25 °C. (iv) **1a** (1 equiv, 0.37 M), EDA (0.9 equiv), BSA (2 equiv), TMAP (2 mol %), trichloroethylene (1.1 equiv), CD_3_CN (537 μL), 25 °C. (v) **1b** (1 equiv, 0.07 M), **4a** (1 equiv), BSA (2 equiv or none), TMAP (0.1 mol %), Bn_2_O (1 equiv), CD_3_CN (600 μL), 25 °C.

Experiments to provide insight into the mechanism of the reaction are summarized in Scheme 4. In the absence of aldehyde **1a**, **2** is *C*-silylated to give **10** but at a rate which is over 5 orders of magnitude slower than the aldol reaction (6.2 ± 0.6 nM min^–1^ vs 0.35 ± 0.02 mM min^–1^, Scheme 4a). The slow rate suggests that **10** is not an intermediate. The aldol reaction between **1a** and **2** proceeds in the absence of TMAP, but the rate is negligible compared to the TMAP/BSA catalytic process (0.62 ± 0.11 nM min^–1^ vs 0.35 ± 0.02 mM min^–1^, Scheme 4a). Interestingly, bis(trimethylsilyl)trifluoroacetamide (BSTFA) provides the aldol product at a rate which is comparable to the rate observed with BSA (Scheme 4a). These four control experiments suggest that the catalytic cycle likely involves an active species generated from BSA or BSTFA and TMAP.

**Scheme 5:**
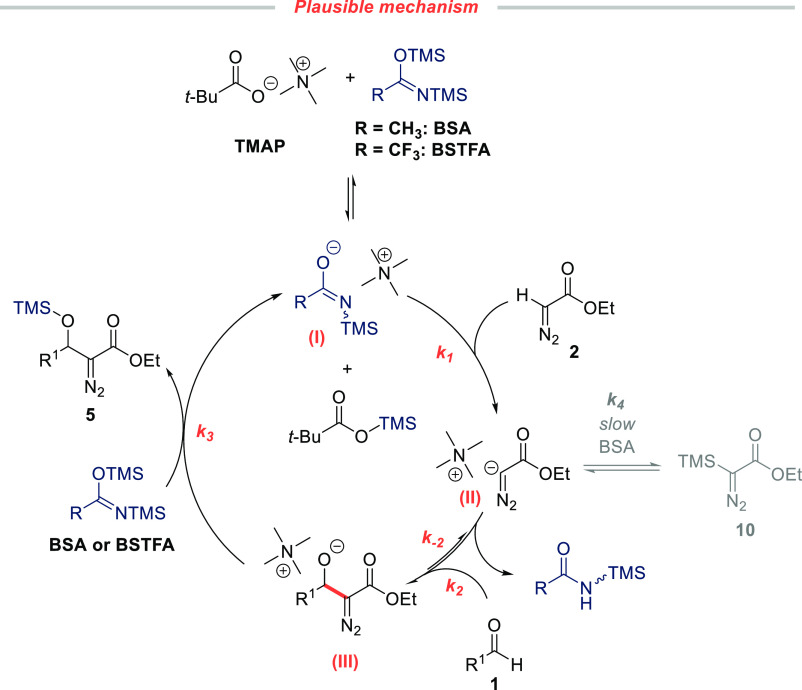
Proposed Catalytic Cycle of the Carboxylate-Catalyzed Silylative Aldol Reaction Involving a Probase Mechanism

The active, on-cycle species could be the desilylated anion derived from BSA/BSTFA and TMAP via silyl transfer to the carboxylate anion (a probase mechanism).^14^ A catalytic cycle consistent with this scenario presented in Scheme 5. We propose that the base deprotonates **2** (*k*_1_ in Scheme 5). However, the turnover rate is not determined by the deprotonation step, since this should lead to a measurable difference between the rates obtained with BSA and BSTFA.

The aldol reaction to give **5a** must also involve C–C bond formation and silylation steps, and these steps might be slower than the proton transfer steps. To explore substituent effects with different aldehyde electrophiles, we examined the relative rates of the formation of **5a**,**c**,**d**,**f** via competition experiments (Scheme 4b, for details, see the Supporting Information).^15^ While a reliable ρ value could not be established from these experiments alone, the reaction was found to be accelerated by electron-withdrawing and decelerated by electron-donating groups (*p*-OMe, *k*_rel_ ≈ 0.11; *p*-Me, *k*_rel_ ≈ 0.13; and *p*-Cl, *k*_rel_ ≈ 4.3 compared to **5a**). These data are consistent with a turnover-limiting nucleophilic addition to the aldehyde carbonyl.

Finally, to examine to which extent the aldol process is reversible, we carried out two crossover experiments (Scheme 4c). In the first experiment, we exposed the desilylated **4a** and aldehyde **1b** to the reaction conditions (Scheme 4c). The results show that there is initial crossover to give **1a** and silylated **5b**, but the crossover process stops within minutes when the products are silylated, and no **4a** could be detected after 5 min. This result suggests that the aldol step might be reversible (*k*_–2_ in Scheme 5), but the silylation step (*k*_3_) is essentially irreversible. The second crossover experiment without BSA gave no crossover products (Scheme 4c), consistent with the probase mechanism.

Taken together, these experiments are consistent with a catalytic cycle (Scheme 5) where the desilylated BSA (**I**) acts as a base, generating the enolate (**II**) which then reacts with aldehyde in the turnover-determining step to form the aldolate (**III**).

## Conclusion

In conclusion, we have established a mild carboxylate-catalyzed silylative diazoaldol reaction that proceeds rapidly at rt with a range of substrates and provides the aldol products within minutes, generally without the need of any chromatographic purification. A probase mechanism where the carboxylate catalyst reacts with the silylating agent to generate an active base catalyst is suggested on the basis of reaction progress studies. Applications of the carboxylate-based probase catalysis in other reactions are ongoing.

## Experimental Section

### General Procedure for the Catalytic Aldol Reaction (GP)

To a solution of aldehyde (limiting reagent, typically 0.98 mmol, 1.0 equiv), ethyl diazoacetate (1.03 mmol, 1.1 equiv), and *N*,*O*-bis(trimethylsilyl)acetamide (1.96 mmol, 2.0 equiv) in dry MeCN was added a freshly prepared solution of tetramethylammonium pivalate (TMAP) (57.3 mM in MeCN, 0.001–0.10 equiv). The reaction progress was monitored by taking small aliquots which were analyzed by ^1^H NMR. After no aldehyde remained in the reaction mixture (typically after 5–25 min), the NMR sample solution was combined with the reaction mixture, EtOAc (10 mL) and water (15 mL) were added, and the layers were separated. The organic phase was dried over Na_2_SO_4_, filtered, and concentrated to give the crude product as a tan/orange oil, and the side product *N*-trimethylsilylacetamide as white crystals. Repeated trituration with EtOAc/hexanes (5:95, ca. 25 mL total), and filtration provided the pure products (typically 2–3 times was sufficient).

#### Ethyl 2-Diazo-3-(trimethylsiloxy)-3-phenylpropanoate (**5a**)

Prepared using the GP, with benzaldehyde (104.0 mg, 980 μmol, 0.1 mL, 1.0 equiv) and TMAP solution (0.45 mL, 9.8 μmol, 0.01 equiv) to give, after 5 min reaction time, 276 mg (93%) of **5a** as a yellow oil. ^1^H NMR (300 MHz, CDCl3) δ 7.43–7.23 (m, 5H), 5.84 (s, 1H), 4.25 (obsd ABX_3_, 2H, Δ*v* = 7.2 Hz, |*J*_AB_| = 10.7 Hz, |*J*_AX_| = |*J*_BX_| = 7.1 Hz), 1.29 (t, *J* = 7.1 Hz, 3H), 0.16 (s, 9H). ^13^C{^1^H} NMR (75 MHz, CDCl_3_) δ 165.7, 141.2, 128.6, 127.9, 125.6, 68.9, 61.0, 14.6, −0.1. Spectral data corresponds to previously published data.^16^

#### Ethyl 2-Diazo-3-(4-(trifluoromethyl)phenyl)-3-((trimethylsilyl)oxy)propanoate (**5b**)

Prepared using the GP, with 4-trifluoromethylbenzaldehyde (158.8 mg, 912 μmol, 0.125 mL, 1.0 equiv) and TMAP solution (0.16 mL, 9.12 μmol, 0.01 equiv) to give, after 5 min reaction time, 282 mg (86%) of **5b** as a yellow oil; ^1^H NMR (300 MHz, CDCl_3_) δ 7.61 (d, *J* = 7.9 Hz, 2H), 7.51 (d, *J* = 8.8 Hz, 1H), 5.87 (s, 1H), 4.27 (obsd ABX_3_, 2H, Δ*v* = 7.9 Hz, |*J*_AB_| = 10.8 Hz, |*J*_AX_| = |*J*_BX_| = 7.1 Hz), 1.30 (t, *J* = 7.1 Hz, 3H), 0.17 (s, 9H); ^13^C{^1^H} NMR (75 MHz, CDCl_3_) δ 165.4, 145.4 (d, *J* = 1.5 Hz), 130.2 (q, *J* = 32.4 Hz), 126.0, 125.7 (q, *J* = 3.8 Hz), 124.2 (d, *J* = 272.0 Hz), 68.4, 61.2, 14.6, −0.1.; IR (neat, ATR): ν_max_ 2093, 1688, 1252 cm^–1^. HRMS (ESI^+^) *m*/*z*: [M + Na]^+^ calculated for C_15_H_19_F_3_N_2_O_3_SiNa^+^ 383.1010, observed 383.1010, Δ = 0.0 ppm.

#### Ethyl 2-Diazo-3-(4-methoxyphenyl)-3-((trimethylsilyl)oxy)propanoate (**5c**)

Prepared using the GP, with 4-methoxybenzaldehyde (126.9 mg, 905 μmol, 0.11 mL, 1.0 equiv) and TMAP solution (0.8 mL, 45.8 μmol, 0.05 equiv) to give, after 15 min reaction time, 280 mg (96%) of **5c** as a yellow oil; ^1^H NMR (300 MHz, CDCl_3_) δ 7.35–7.23 (m, 2H), 6.93–6.82 (m, 2H), 5.79 (s, 1H), 4.26 (obsd ABX_3_, 2H, Δ*v* = 7.0 Hz, |*J*_AB_| = 10.7 Hz, |*J*_AX_| = |*J*_BX_| = 7.1 Hz), 3.80 (s, 3H), 1.29 (t, *J* = 7.1 Hz, 3H), 0.14 (s, 9H); ^13^C{^1^H} NMR (75 MHz, CDCl_3_) δ 165.8, 159.3, 133.4, 126.8, 114.0, 68.6, 60.9, 55.4, 14.7, −0.1. IR (neat, ATR): ν_max_ 2088, 1687, 1251 cm^–1^. HRMS (ESI^+^) *m*/*z*: [M + Na]^+^ calculated for C_15_H_22_N_2_O_4_SiNa^+^ 345.1242, observed 345.1251, Δ = −2.6 ppm.

#### Ethyl 2-Diazo-3-(*p*-tolyl)-3-((trimethylsilyl)oxy)propanoate (**5d**)

Prepared using the GP, with 4-methylbenzaldehyde (112.0 mg, 933 μmol, 0.11 mL, 1.0 equiv) and TMAP solution (0.16 mL, 9.2 μmol, 0.01 equiv) to give, after 15 min reaction time, 276 mg (97%) of **5d** as a yellow oil; ^1^H NMR (300 MHz, CDCl_3_) δ 7.27 (d, *J* = 8.1 Hz, 2H), 7.15 (d, *J* = 8.0 Hz, 2H), 5.81 (s, 1H), 4.26 (obsd ABX_3_, 2H, Δ*v* = 6.8 Hz, |*J*_AB_| = 10.7 Hz, |*J*_AX_| = |*J*_BX_| = 7.1 Hz), 2.34 (s, 3H), 1.29 (t, *J* = 7.1 Hz, 3H), 0.15 (s, 9H). ^13^C{^1^H} NMR (75 MHz, CDCl_3_) δ 165.8, 138.3, 137.6, 129.3, 125.5, 68.8, 60.9, 21.2, 14.7, −0.1. IR (neat, ATR) ν_max_ 2090, 1688, 1250 cm^–1^. HRMS (ESI^+^) *m*/*z*: [M + Na]^+^ calculated for C_15_H_22_N_2_O_3_SiNa^+^ 329.1292, observed 329.1285, Δ = 2.1 ppm.

#### Ethyl 3-(3-Bromophenyl)-2-diazo-3-((trimethylsilyl)oxy)propanoate (**5e**)

Prepared using the GP, with 3-bromobenzaldehyde (1.0 equiv, 175 mg, 943 μmol, 0.11 mL) and TMAP solution (0.8 mL, 45.8 μmol, 0.05 equiv) to give, after 15 min reaction time, 350 mg (quant) of **5e** as a yellow oil; ^1^H NMR (300 MHz, CD_3_CN) δ 7.67–7.54 (m, 1H), 7.54–7.24 (m, 3H), 5.83 (s, 1H), 4.22 (obsd ABX_3_, 2H, Δ*v* = 7.4 Hz, |*J*_AB_| = 10.8 Hz, |*J*_AX_| = |*J*_BX_| = 7.1 Hz), 1.25 (t, *J* = 7.1 Hz, 3H), 0.14 (s, 9H); ^13^C{^1^H} NMR (75 MHz, CD_3_CN) δ 165.8, 144.9, 131.9, 131.5, 129.5, 125.6, 123.0, 69.0, 62.0, 14.8, −0.2.; IR (neat, ATR): 2090, 1688, 1250 cm^–1^. HRMS (ESI^+^) *m*/*z*: [M + Na]^+^ calculated for C_14_H_19_BrN_2_O_3_SiNa^+^ 393.0241, observed 393.0231, Δ = 2.5 ppm.

#### Ethyl 3-(4-Chlorophenyl)-2-diazo-3-((trimethylsilyl)oxy)propanoate (**5f**)

Prepared using the GP, with 4-chlorobenzaldehyde (1.0 equiv, 131.7 mg, 937 μmol) and TMAP solution (0.8 mL, 45.8 μmol, 0.05 equiv) to give, after 15 min reaction time, 306 mg (quant) of **5f** as a yellow oil; ^1^H NMR (300 MHz, CD_3_CN) δ 7.41–7.36 (m, 4H), 5.84 (s, 1H), 4.22 (obsd ABX_3_, 2H, Δ*v* = 7.0 Hz, |*J*_AB_| = 10.8 Hz, |*J*_AX_| = |*J*_BX_| = 7.1 Hz), 1.25 (t, *J* = 7.1 Hz, 3H), 0.14 (s, 9H); ^13^C{^1^H} NMR (75 MHz, CD_3_CN) δ 165.9, 141.2, 134.1, 129.5, 128.4, 69.2, 62.0, 14.8, −0.2; IR (neat, ATR); 2092, 1690, 1249 cm^–1^. HRMS (ESI^+^) *m*/*z*: [M + Na]^+^ calculated for C_14_H_19_ClN_2_O_3_SiNa^+^ 349.0746, observed 349.0736, Δ = 2.9 ppm.

#### Ethyl 2-Diazo-3-((trimethylsilyl)oxy)-3-(4-((trimethylsilyl)oxy)phenyl)propanoate (**5g**)

Prepared using the GP, with 4-hydroxybenzaldehyde (1.0 equiv, 112.5 mg, 921 μmol), BSA (2.7 equiv, 499 mg, 2450 μmol, 0.6 mL) and TMAP solution (0.8 mL, 45.8 μmol, 0.05 equiv) to give, after 15 min reaction time, 346 mg (99%) of **5g** as a yellow oil; ^1^H NMR (300 MHz, CD_3_CN) δ 7.33–7.21 (m, 2H), 6.91–6.80 (m, 2H), 5.79 (s, 1H), 4.22 (obsd ABX_3_, 2H, Δ*v* = 6.4 Hz, |*J*_AB_| = 8.9 Hz, |*J*_AX_| = |*J*_BX_| = 7.1 Hz), 1.25 (t, *J* = 7.1 Hz, 3H), 0.24 (s, 9H), 0.13 (s, 9H); ^13^C{^1^H} NMR (75 MHz, CD_3_CN) δ 166.1, 156.0, 135.2, 127.9, 121.0, 69.4, 61.9, 14.9, 0.2, −0.1.; IR (neat, ATR); 2090, 1688, 1250 cm^–1^. HRMS (ESI^+^) *m*/*z*: [M + Na]^+^ calculated for C_17_H_28_N_2_O_4_Si_2_Na^+^ 403.1480, observed 403.1476, Δ = 1.0 ppm.

#### Ethyl 2-Diazo-5-phenyl-3-((trimethylsilyl)oxy)pentanoate (**5h**)

Prepared using the GP, with hydrocinnamaldehyde (127.4 mg, 949 μmol, 0.125 mL, 1.0 equiv) and TMAP solution (0.8 mL, 45.8 μmol, 0.05 equiv) to give, after 10 min reaction time, 233 mg (77%) of **5h** as a yellow oil; ^1^H NMR (300 MHz, CDCl_3_) δ 7.36–7.09 (m, 7H), 4.66 (ddd, *J* = 7.4, 6.0, 1.8 Hz, 1H), 4.24 (obsd ABX_3_, 2H, Δ*v* = 5.1 Hz, |*J*_AB_| = 10.8 Hz, |*J*_AX_| = |*J*_BX_| = 7.1 Hz), 2.86–2.51 (m, 3H), 2.07–1.87 (m, 2H), 1.28 (t, *J* = 7.1 Hz, 3H), 0.13 (s, 9H); ^13^C{^1^H} NMR (75 MHz, CDCl_3_) δ 165.8, 141.4, 128.5, 128.5, 126.1, 66.4, 60.9, 38.1, 32.0, 14.6, −0.1.; IR (neat): 2089, 1688, 1251 cm^–1^. HRMS (ESI^+^) *m*/*z*: [M + Na]^+^ calculated for C_16_H_24_N_2_O_3_SiNa^+^ 343.1449, observed 343.1447, Δ = 0.6 ppm.

#### Ethyl 2-Diazo-3-((trimethylsilyl)oxy)dodecanoate (**5i**)

Prepared using the GP, with *n*-decanal (145.3 mg, 949 μmol, 0.175 mL, 1.0 equiv) and TMAP solution (0.8 mL, 45.8 μmol, 0.05 equiv) to give, after 10 min reaction time, 277 mg (87%) of **5i** as a yellow oil; ^1^H NMR (300 MHz, CDCl_3_) δ 4.57 (dd, *J* = 7.3, 6.2 Hz, 1H), 4.23 (obsd ABX_3_, 2H, Δ*v* = 6.1 Hz, |*J*_AB_| = 10.8 Hz, |*J*_AX_| = |*J*_BX_| = 7.1 Hz),1.39–1.15 (m, 19H), 0.88 (t, *J* = 6.8 Hz, 3H), 0.10 (s, 9H); ^13^C{^1^H} NMR (75 MHz, CDCl_3_) δ 166.0, 66.9, 60.8, 36.4, 32.0, 29.7, 29.7, 29.4, 29.4, 25.7, 22.8, 14.7, 14.2, −0.1; IR (neat, ATR): 2088, 1692, 1251 cm^–1^. HRMS (ESI^+^) *m*/*z*: [M + Na]^+^ calculated for C_17_H_34_N_2_O_3_SiNa^+^ 365.2231, observed 365.2238, Δ = −1.9 ppm.

#### Ethyl 2-Diazo-5-methyl-3-((trimethylsilyl)oxy)hexanoate (**5j**)

Prepared using the GP, with isovaleraldehyde (80.3 mg, 932 μmol, 0.10 mL, 1.0 equiv) and TMAP solution (0.8 mL, 45.8 μmol, 0.05 equiv) to give, after 10 min reaction time, 218 mg (86%) of **5j** as a yellow oil; ^1^H NMR (300 MHz, CDCl_3_) δ 4.69 (dd, *J* = 7.8, 6.1 Hz, 1H), 4.22 (obsd ABX_3_, 2H, Δ*v* = 7.8 Hz, |*J*_AB_| = 10.8 Hz, |*J*_AX_| = |*J*_BX_| = 7.1 Hz), 1.75–1.49 (m, 2H), 1.49–1.34 (m, 1H), 1.27 (t, *J* = 7.1 Hz, 3H), 0.92 (obsd d, *J* = 6.5 Hz, 3H), 0.91 (obsd d, *J* = 6.5 Hz, 3H), 0.12 (s, 9H); ^13^C{^1^H} NMR (75 MHz, CDCl_3_) δ 165.9, 77.6, 77.2, 76.7, 65.4, 60.8, 45.3, 24.8, 23.0, 22.3, 14.7, −0.1; IR (neat, ATR): 2088, 1691, 1251 cm^–1^. HRMS (ESI^+^) *m*/*z*: [M + Na]^+^ calculated for C_12_H_24_N_2_O_3_SiNa^+^ 295.1449, observed 295.1437, Δ = 4.1 ppm.

#### Ethyl 2-Diazo-4,4-dimethyl-3-((tfrimethylsilyl)oxy)pentanoate (**5k**)

Prepared using the GP, with pivalaldehyde (80 mg, 921 μmol, 0.1 mL, 1.0 equiv) and TMAP solution (0.8 mL, 45.8 μmol, 0.05 equiv) to give, after 10 min reaction time, 215 mg (86%) of **5k** as a yellow oil; ^1^H NMR (300 MHz, CDCl_3_) δ 4.21 (obsd ABX_3_, 2H, Δ*v* = 7.5 Hz, |*J*_AB_| = 10.8 Hz, |*J*_AX_| = |*J*_BX_| = 7.1 Hz), 4.18 (s, 1H 1.26 (t, *J* = 7.1 Hz, 3H), 0.89 (s, 9H), 0.10 (s, 9H); ^13^C{^1^H} NMR (75 MHz, CDCl_3_) δ 166.5, 73.4, 60.7, 38.8, 25.7, 14.7, −0.5; IR (neat, ATR): 2087, 1691, 1252 cm^–1^. HRMS (ESI^+^) *m*/*z*: [M + Na]^+^ calculated for C_12_H_24_N_2_O_3_SiNa^+^ 295.1449, observed 295.1445, Δ = 1.4 ppm.

#### Ethyl 2-Diazo-3-(furan-2-yl)-3-((trimethylsilyl)oxy)propanoate (**5l**)

Prepared using the GP, with furfural (87.0 mg, 905 μmol, 0.075 mL) and TMAP solution (0.8 mL, 45.8 μmol, 0.05 equiv) to give, after 25 min reaction time, 255 mg (quant) of **5l** as a yellow oil; ^1^H NMR (300 MHz, CDCl_3_) δ 7.34 (dd, *J* = 1.8, 0.9 Hz, 1H), 6.32 (dd, *J* = 3.3, 1.8 Hz, 1H), 6.28 (dt, *J* = 3.2, 0.9 Hz, 1H), 5.74 (d, *J* = 0.8 Hz, 1H), 4.22 (q, *J* = 7.1 Hz, 2H), 1.26 (t, *J* = 7.1 Hz, 3H), 0.12 (s, 9H); ^13^C{^1^H} NMR (75 MHz, CDCl_3_) δ 165.3, 153.4, 142.6, 110.3, 107.0, 63.7, 61.1, 14.6, −0.2; IR (neat, ATR): 2095, 1690, 1252 cm^–1^. HRMS HRMS (ESI^+^) *m*/*z*: [M + Na]^+^ calculated for C_12_H_18_N_2_O_4_SiNa^+^ 305.0929, observed 305.0920, Δ = 2.9 ppm.

#### Ethyl 2-Diazo-3-((trimethylsilyl)oxy)-3-(5-(((trimethylsilyl)oxy)methyl)furan-2-yl)propanoate (**5m**)

Prepared using the GP, with 5-(hydroxymethyl)furan-2-carbaldehyde (380 mg, 1 mmol, 1.0 equiv), BSA (3 equiv, 610 mg, 3 mmol, 0.733 mL), and TMAP solution (0.8 mL, 45.8 μmol, 0.05 equiv) to give, after 30 min reaction time, 380 mg (98%) of **5l** as a yellow oil. ^1^H NMR (300 MHz, CDCl_3_) δ 6.21 (d, *J* = 3.2 Hz, 1H), 6.18 (d, *J* = 3.2 Hz, 1H), 5.72 (d, *J* = 0.8 Hz, 2H), 4.57 (s, 1H), 4.25 (q, *J* = 7.1 Hz, 2H), 1.28 (t, *J* = 7.1 Hz, 3H), 0.15 (s, 9H), 0.12 (s, 9H). ^13^C{^1^H} NMR (75 MHz, CDCl_3_) δ 165.4, 154.1, 153.0, 108.4, 107.8, 63.8, 61.1, 57.6, 14.7, −0.1, −0.3. IR (neat, ATR): 2097, 1693, 1250 cm^–1^. HRMS (ESI^+^) *m*/*z*: [M + Na]^+^ calculated for C_16_H_28_N_2_O_5_Si_2_Na^+^ 407.1429, observed 407.1422, Δ = 1.7 ppm.

Another batch of **5m** was prepared on a larger scale, using 630 mg (5.0 mmol) of 5-(hydroxymethyl)furan-2-carbaldehyde, 3.67 mL (3 equiv, 15 mmol) of BSA, and TMAP solution (44 mg, 230.0 μmol, 0.05 equiv in 6 mL of MeCN) after 30 min to afford **5m** (1.76 g, 92%) as a yellow oil. The ^1^H NMR spectrum of the resulting **5m** fully matched the data obtained in the small scale batch.

#### *tert-*Butyl (*R*)*-*3-((*R*)-2-Diazo-3-ethoxy-3-oxo-1-((trimethylsilyl)oxy)propyl)-1-oxa-4-azaspiro[4.5]decane-4-carboxylate (**5n**)

Prepared using the GP, with *tert*-butyl (*R*)-3-formyl-1-oxa-4-azaspiro[4.5]decane-4-carboxylate (248.9 mg, 920 μmol, 1.0 equiv) and TMAP solution (0.8 mL, 45.8 μmol, 0.05 equiv) to give, after 15 min reaction time, 397 mg (92% mass balance) of slightly impure **5n** as a yellow oil. The crude product was purified by CombiFlash chromatography (hexane:EtOAc = 100:0 to 80:20) to give 265 mg (63%) of **5n** as a thick yellow oil; ^1^H NMR (500 MHz, CD_3_CN, major isomer) δ 4.60 (d, *J* = 7.9 Hz, 1H), 4.18 (obsd ABX_3_, 2H, Δ*v* = 5.4 Hz, |*J*_AB_| = 10.9 Hz, |*J*_AX_| = |*J*_BX_| = 7.2 Hz), 4.02–3.96 (m, 1H), 3.90 (qd, *J* = 9.2, 3.3 Hz, 2H), 2.37–2.11 (m, 2H), 1.70–1.48 (m, 8H), 1.45 (s, 9H), 1.23 (t, *J* = 7.1 Hz, 3H), 0.15(s, 9H). A diagnostic signal corresponding to minor (*R*,*S*)-isomer is observed at δ 4.98 (d, *J* = 4.8 Hz, 0.09 H). ^13^C{^1^H} NMR (126 MHz, CD_3_CN, 328 K) δ 166.5, 153.9, 96.5, 81.1, 68.3, 65.6, 61.8, 61.2, 36.8, 31.8, 28.8, 26.0, 24.48, 24,46, 15.1, −0.2. IR (neat, ATR): 2099, 1251 cm^–1^. HRMS (ESI^+^) *m*/*z*: [M + Na]^+^ calculated for C_21_H_37_N_3_O_6_SiNa+ 478.2344, observed 478.2345, Δ = −0.2 ppm. [α]_D_^20^ = −15.4 (*c* = 1.0, CH_2_Cl_2_).

Another batch of **5n** was prepared using the GP, but the reaction was carried out at 0 °C (30 min reaction time) to give **5n** (350 mg, 82%) with a similar 10:1 diastereomeric purity. The enantiomeric purity of **5n** was determined from the corresponding desilylated derivatives **6** (see below).

#### *tert-*Butyl (*R*)-3-((*R*)-2-Diazo-3-ethoxy-1-hydroxy-3-oxopropyl)-1-oxa-4-azaspiro[4.5]decane-4-carboxylate (**6**)

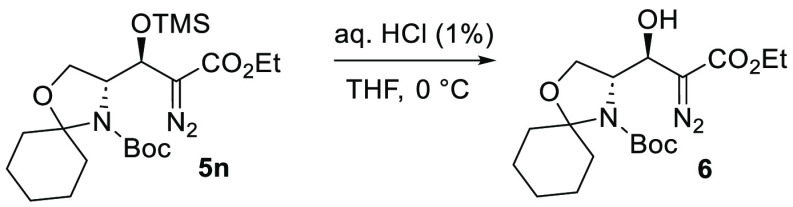



To a cooled solution of **5n** (30 mg, 66 μmol, 1.0 equiv) in THF (1 mL), aq 1% HCl was added dropwise (480 μL, 2 equiv). The resulting mixture was stirred for 30 min at 0 °C. The reaction was monitored by TLC (hexane:EtOAc = 95:5 to 80:20). DCM (15 mL) was added, and the mixture was washed with water (3 × 15 mL). The organic phase was dried over Na_2_SO_4_, filtered, and concentrated. The crude mixture was purified by CombiFlash chromatography (hexane:EtOAc = 95:5 to 80:20) to give **6** (21 mg, 84%) as a yellow oil. [α]_D_^20^ = +23.3 (*c* = 0.2, CH_2_Cl_2_); ^1^H NMR (300 MHz, CD_3_CN, 323 K) δ 4.51 (t, *J* = 6.1 Hz, 1H), 4.18 (q, *J* = 7.1 Hz, 2H), 4.15–4.05 (m, 1H), 4.03–3.87 (m, 2H), 2.34–2.06 (m, 2H), 1.74–1.47 (m, 6H), 1.46 (s, 9H), 1.23 (t, *J* = 7.1 Hz, 3H), 1.32–1.09 (m, 1H). ^13^C{^1^H} NMR (75 MHz, CD_3_CN, 328 K) 167.0, 155.0, 97.0, 81.8, 68.4, 65.8, 61.7, 61.5 (low intensity), 36.5, 31.5, 28.8, 26.0, 24.5, 24.4, 15.0. IR (neat, ATR): bs 3432, 2098 cm^–1^. HRMS (ESI^+^) *m*/*z*: [M + Na]^+^ calculated for C_18_H_29_N_3_O_6_Na^+^ 406.1949, observed 406.1958, Δ = −2.2 ppm. HPLC (CHIRALCEL OZ-H, *c* = 1 mg/mL, *v* = 1 mL/min, 22 °C): *t*_R_ = 46.5 min (*R*,*S*)-isomer; *t*_R_ = 52.5 min (*R*,*R*)-isomer, *t*_R_ = 36.3 min (*S*,*S*)-isomer, er = 97:3, dr = 91:9.

Note 1: The desilylation and purification protocol results may enrich the diastereomeric ratio of the compound from ca. 91:9 to ca. 97:3, depending on the rigorousness of the purification.

Note 2: The same procedure was applied to the batch of **6** obtained at 0 °C.

### Stereochemical Assignments, Derivatization of **5n**, and Preparation of Racemic Samples

#### *tert*-Butyl (*R*)-3-((*S*)-3-Ethoxy-3-oxo-1-((trimethylsilyl)oxy)propyl)-1-oxa-4-azaspiro[4.5]decane-4-carboxylate (**7**)

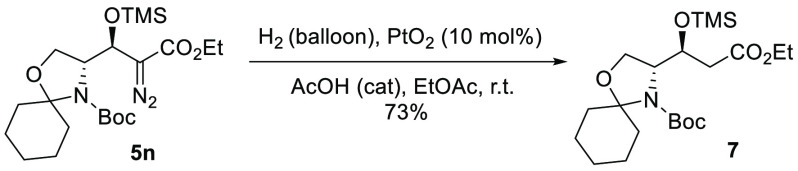



The reduction reaction was carried out using the following modified literature procedure.^17^ To a degassed solution of **5n** (160 mg, 0.35 mmol, 1 equiv) in EtOAc (3 mL) were added platinum dioxide (8 mg, 0.035 mmol, 0.1 equiv) and a catalytic amount (a drop) of acetic acid at room temperature. The flask was purged with argon and then with hydrogen gas, and the heterogeneous reaction mixture was stirred under hydrogen atmosphere (balloon) for 4 h. The reaction was monitored by TLC (hexanes:EtOAc = 95:5, anisaldehyde stain). The reaction vessel was purged with argon, and the mixture was filtered through neutral Celite, concentrated in vacuo, and purified by silica gel chromatography (hexane:EtOAc = 95:5, anisaldehyde stain) to afford **7** 105 mg (73%) as a colorless oil. ^1^H NMR (300 MHz, CD_3_CN) δ 4.44 (m, 1H), 4.09 (q, *J* = 7.2 Hz, 2H), 3.97–3.89 (m, 1H), 3.88–3.76 (m, 2H), 2.53–2.28 (m, 2H), 2.27–2.13 (m, 2H), 1.57 (m, 8H), 1.46 (s, 9H), 1.22 (t, *J* = 7.1 Hz, 3H), 0.08 (s, 9H). ^13^C{^1^H} NMR (75 MHz, CD_3_CN) δ 172.2, 153.7, 96.3, 80.8, 70.1, 63.9, 62.2, 61.3, 41.6, 36.1, 28.7, 25.8, 24.2, 14.5, 0.5. IR (neat, ATR): 1736, 1692, 1250 cm^–1^. HRMS (ESI^+^) *m*/*z*: [M + Na]^+^ calculated for C_21_H_39_NO_6_SiNa^+^ 452.2439, observed 452.2439, Δ = 0.0 ppm. [α]_D_^20^ = +13.3 (*c* = 1.0, CH_2_Cl_2_).

#### *tert*-Butyl 3-((*S*)-3-Ethoxy-1-hydroxy-3-oxopropyl)-1-oxa-4-azaspiro[4.5]decane-4-carboxylate (**8**)

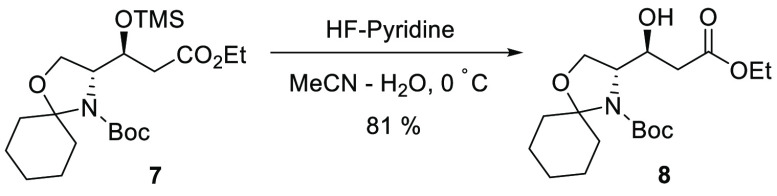



To a cooled solution of **7** (45 mg, 0.104 mmol, 1 equiv) in MeCN–H_2_O (3 mL, 95:5 vol) was added HF–pyridine solution (1.9 μL, 0.2 equiv) by one portion at 0 °C and stirred for 30 min at 0 °C. The reaction was monitored by TLC (hexanes:EtOAc = 95:5, KMnO_4_ stain). H_2_O (5 mL) was added, and the resulting mixture was extracted with DCM (10 mL). The organic layer was washed by satd NaHCO_3_ (5 mL), dried over Na_2_SO_4_, and concentrated under high vacuum to give crude **8** (30 mg, 81%) as a colorless oil, which used further without purification. ^1^H NMR (300 MHz, CD_3_CN) δ 4.17–4.01 (m, 1H), 4.12 (q, *J* = 7.1 Hz, 2H), 4.02–3.78 (m, 3H), 2.46 (dd, *J* = 15.5, 3.4 Hz, 1H), 2.40–2.16 (m, 3H), 1.75–1.51 (m, 3H), 1.78–1.38 (m, 15H), 1.34–1.08 (m, 2H), 1.23 (t, *J* = 7.1 Hz, 3H). ^13^C{^1^H} NMR (75 MHz, CD_3_CN) δ 172.8, 154.7, 96.3, 81.2, 70.3, 64.9, 62.1, 61.2, 40.0, 36.2, 28.6, 25.7, 24.3, 14.5. HRMS (ESI^+^) *m*/*z*: [M+K]^+^ calculated for C_18_H_31_NO_6_K^+^ 396.1783, observed 396.1784, Δ = −0.3 ppm.

#### Ethyl 2-((4*S*,5*R*)-5-((*tert*-Butoxycarbonyl)amino)-2,2-dimethyl-1,3-dioxan-4-yl)acetate (**9a**) and Ethyl 2-((2*S*,3*R*)-3-((*tert*-Butoxycarbonyl)amino)-1,5-dioxaspiro[5.5]undecan-2-yl)acetate (**9b**)

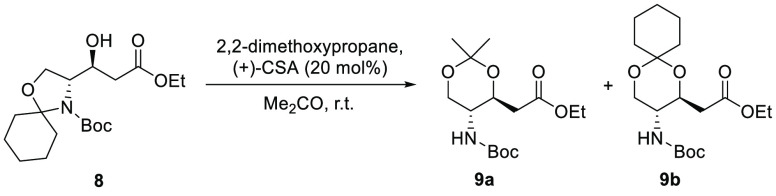



To a solution of **8** (31 mg, 0.08 mmol, 1 equiv) and 2,2-dimethoxypropane (106 μL, 0.8 mmol, 10 equiv) in acetone (2 mL) was added (+)-camphorsulfonic acid (4 mg, 0.02 mmol, 0.2 equiv) in one portion. The reaction mixture was stirred for 48 h at rt. The reaction was monitored by TLC (hexane:EtOAc = 90:10, anisaldehyde stain). The mixture was diluted with EtOAc (10 mL), and the organic layer was washed with NaHCO_3_, water and brine (10 mL each), dried over Na_2_SO_4_, and concentrated in vacuo to give a residue which was purified by silica gel chromatography (hexane:EtOAc = from 90:10 to 80:20) to afford **9a** (20 mg, 73% yield) and **9b** (4 mg, 13% yield).

**9a**: ^1^H NMR (300 MHz, CDCl_3_) δ 4.44 (bs, 1H), 4.15 (app. qd, *J* = 7.1, 1.4 Hz, 2H), 4.06 (ddd, *J* = 9.7, 8.2, 3.9 Hz, 1H), 3.99–3.85 (m, 1H), 3.66–3.46 (m, 2H), 2.67 (dd, *J* = 16.0, 3.9 Hz, 1H), 2.50 (dd, *J* = 16.1, 8.3 Hz, 1H), 1.45 (s, 3H), 1.43 (s, 9H), 1.37 (s, 3H), 1.26 (t, *J* = 7.1 Hz, 3H). ^13^C{^1^H} NMR (75 MHz, CDCl_3_) δ 171.5, 155.3, 99.2, 80.1, 70.0, 63.6, 60.8, 49.4, 38.8, 28.5, 19.7, 14.3. IR (CHCl_3_ soln, ATR): 3345, 1713 cm^–1^. HRMS (ESI^+^) *m*/*z*: [M + K]^+^ calculated for C_15_H_27_NO_6_K^+^ 356.1470, observed 356.1467, Δ = 0.8 ppm. [α]_D_^20^ = −11.6 (*c* = 1.6, CHCl_3_).

**9b**: ^1^H NMR (500 MHz, CDCl_3_) δ 4.40 (bs, 1H), 4.14 (obsd ABX_3_, 2H, Δ*v* = 14.8 Hz, |*J*_AB_| = 11.7 Hz, |*J*_AX_| = |*J*_BX_| = 7.1 Hz), 4.06 (td, *J* = 9.3, 3.5 Hz, 1H), 3.95–3.85 (m, 1H), 3.64–3.53 (m, 2H), 2.66 (dd, J = 15.8, 3.5 Hz, 1H) 2.51 (dd, *J* = 15.8, 9.1 Hz, 1H), 1.72–1.37 (m, 19H), 1.27 (t, *J* = 7.1 Hz, 3H). ^13^C{^1^H} NMR (101 MHz, CDCl_3_) δ 171.7, 155.2, 99.2, 80.1, 69.5, 62.8, 60.8, 49.3, 38.9, 37.3, 28.5, 25.7, 22.8, 22.5, 14.4. IR (CHCl_3_ soln, ATR): 1710, 1214 cm^–1^. HRMS (ESI^+^) *m*/*z*: [M + K]^+^ calculated for C_18_H_31_NO_6_K^+^ 396.1783, observed 396.1788, Δ = −1.3 ppm. [α]_D_^20^= −13.8 (*c* = 0.4, CHCl_3_).

#### (±)*-tert*-Butyl 3-(2-diazo-3-ethoxy-3-oxo-1-((trimethylsilyl)oxy)propyl)-1-oxa-4-azaspiro[4.5]decane-4-carboxylate ((±)-**5n**)

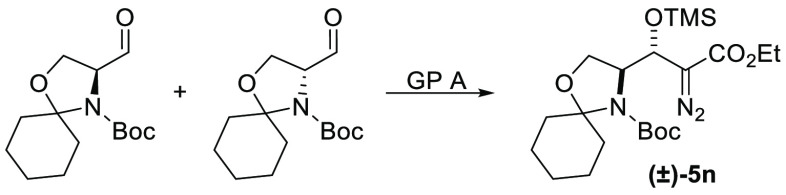



Following the GP, a mixture of (*R*)*-tert*-butyl 3-formyl-1-oxa-4-azaspiro[4.5]decane-4-carboxylate (124.5 mg, 460 μmol, 0.5 equiv) and (*S*)*-tert*-butyl 3-formyl-1-oxa-4-azaspiro[4.5]decane-4-carboxylate (124.5 mg, 460 μmol, 0.5 equiv) was subjected to the diazoaldol reaction to give, after 15 min reaction time and purification (CombiFlash chromatography, hexane:EtOAc = 100:0 to 95:5), a racemic mixture of (±)-**5n**, 368 mg (87%) as a yellow crystals. ^1^H NMR (300 MHz, CD_3_CN, 323 K, major diastereomer) δ 4.54 (d, *J* = 8.3 Hz, 1H), 4.16 (q, *J* = 7.1 Hz, 2H), 4.01–3.93 (m, 1H), 3.92–3.88 (m, 2H), 2.30–2.05 (m, 2H), 1.73–1.05 (m, 8H), 1.43 (s, 9H), 1.21 (t, *J* = 7.1 Hz, 3H), 0.12 (s, 9H). ^13^C{^1^H} NMR (75 MHz, CD_3_CN, 323 K, major diastereomer) δ 166.5, 153.8, 96.5, 81.1, 68.2, 65.6, 61.8, 61.2, 36.7, 31.8, 28.8, 26.0, 24.4(8), 24,4(7) 15.1, −0.2. IR (neat, ATR): 2110, 1251 cm^–1^. HRMS (ESI^+^) *m*/*z*: [M + Na]^+^ calculated for C_21_H_37_N_3_O_6_SiNa^+^ 478.2344, observed 478.2338, Δ = 1.3 ppm.

Another batch of (±)-**5n** was prepared following the GP with the exception that the reaction temperature was 0 °C (30 min reaction time). NMR spectra of this batch correspond to those obtained for the batch obtained at rt.

#### (±)*-tert*-Butyl 3-(2-Diazo-3-ethoxy-1-hydroxy-3-oxopropyl)-1-oxa-4-azaspiro[4.5]decane-4-carboxylate ((±)-**6**)

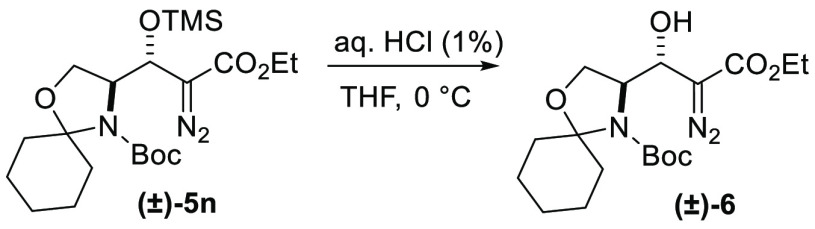



Following the deprotection procedure for **5n** using (±)-**5n** (100 mg, 0.22 mmol), compound (±)-**6** 73 mg (87%) was obtained as a yellow oil. ^1^H NMR (300 MHz, CD_3_CN, 323 K, major diastereomer) δ 4.48 (t, *J* = 6.3 Hz, 1H), 4.25–4.03 (m, 3H), 4.01–3.78 (m, 2H), 2.33–1.99 (m, 2H), 1.72–1.49 (m, 8H), 1.44 (s, 9H), 1.21 (t, *J* = 7.1 Hz, 3H). ^13^C{^1^H} NMR (75 MHz, CD_3_CN, 323 K, major diastereomer) δ 167.0, 154.9, 96.9, 81.8, 68.4, 65.8, 61.7(9), 61.6(6), 36.4, 31.5, 28.8, 25.9, 24.5, 24.4, 15.0. IR (neat, ATR): bs 3430, 2098 cm^–1^. HRMS (ESI^+^) *m*/*z*: [M + Na]^+^ calculated for C_18_H_29_N_3_O_6_Na^+^ 406.1949, observed 406.1944, Δ = 1.2 ppm. HPLC (CHIRALCEL OZ-H, *c* = 1 mg/mL, *v* = 1 mL/min, 22 °C) *t*_R_ = 25.6 min (*S*,*R*)-isomer, *t*_R_ = 39.5 min (*R*,*S*)-isomer, *t*_R_ = 45.8 min (*R*,*R*)-isomer, *t*_R_ = 31.1 min (*S*,*S*)-isomer. er = 1:1, dr = 91:9.

## Data Availability

The data underlying this study are available in the published article and its Supporting Information.

## Supporting Information Available

Copies of NMR spectra, HPLC chromatograms, and details of control and competition experiments (PDF)
FAIR data, including the primary NMR FID files, for compounds **5a**–**n**, **6**–**8**, **9a**, **9b**, (±)-**5n**, (±)-**6** (ZIP)
