# Supplementary material for: Carboxylate Catalysis: A Catalytic O-Silylative Aldol Reaction of Aldehydes and Ethyl Diazoacetate
Source: J Org Chem. 2023 Sep 28;88(20):14396–403. doi: 10.1021/acs.joc.3c01304 (PMC10594658; doi:10.1021/acs.joc.3c01304)

Supporting Information

Carboxylate Catalysis: a Catalytic *O*-Silylative Aldol reaction of Aldehydes and Ethyl Diazoacetate

Riuttamäki, Saara E.; Bannykh, Anton; Pihko, Petri M.*
Department of Chemistry and NanoScience Center, P.O.B. 35, FI-40014 University of Jyväskylä, FINLAND

Petri.Pihko@jyu.fi

Contents

1 General Information 6

2 NMR Spectra, HPLC Chromatograms, and Control Experiments 6

2.1.1 Ethyl 2-diazo-3-phenyl-3-((trimethylsilyl)oxy)propanoate (**5a**), ^{1}H NMR spectrum 6

2.1.2 Ethyl 2-diazo-3-phenyl-3-((trimethylsilyl)oxy)propanoate (**5a**), ^{13}C{^{1}H} NMR spectrum 7

2.1.3 Ethyl 2-diazo-3-(4-(trifluoromethyl)phenyl)-3-((trimethylsilyl)oxy)propanoate (**5b**), ^{1}H NMR spectrum 8

2.1.4 Ethyl 2-diazo-3-(4-(trifluoromethyl)phenyl)-3-((trimethylsilyl)oxy)propanoate (**5b**), ^{13}C{^{1}H} NMR spectrum 9

2.1.5 Ethyl 2-diazo-3-(4-methoxyphenyl)-3-((trimethylsilyl)oxy)propanoate (**5c**), ^{1}H NMR spectrum 10

2.1.6 Ethyl 2-diazo-3-(4-methoxyphenyl)-3-((trimethylsilyl)oxy)propanoate, (**5c**), ^{13}C{^{1}H} NMR spectrum 11

2.1.7 Ethyl 2-diazo-3-(p-tolyl)-3-((trimethylsilyl)oxy)propanoate (**5d**), ^{1}H NMR spectrum 12

2.1.8 Ethyl 2-diazo-3-(p-tolyl)-3-((trimethylsilyl)oxy)propanoate (**5d**), ^{13}C{^{1}H} NMR spectrum 13

2.1.9 Ethyl 3-(3-bromophenyl)-2-diazo-3-((trimethylsilyl)oxy)propanoate (**5e**), ^{1}H NMR spectrum 14

2.1.10 Ethyl 3-(3-bromophenyl)-2-diazo-3-((trimethylsilyl)oxy)propanoate (**5e**), ^{13}C{^{1}H} NMR spectrum 15

2.1.11 Ethyl 3-(4-chlorophenyl)-2-diazo-3-((trimethylsilyl)oxy)propanoate (**5f**), ^{1}H NMR spectrum 16

2.1.12 Ethyl 3-(4-chlorophenyl)-2-diazo-3-((trimethylsilyl)oxy)propanoate (**5f**), ^{13}C{^{1}H} NMR spectrum 17

2.1.13 Ethyl 2-diazo-3-((trimethylsilyl)oxy)-3-(4-((trimethylsilyl)oxy)phenyl)propanoate (**5g**), ^{1}H NMR spectrum 18

2.1.14 Ethyl 2-diazo-3-((trimethylsilyl)oxy)-3-(4-((trimethylsilyl)oxy)phenyl)propanoate (**5g**), ^{13}C{^{1}H} NMR spectrum 19

2.1.15 Ethyl 2-diazo-5-phenyl-3-((trimethylsilyl)oxy)pentanoate (**5h**), ^{1}H NMR spectrum 20

2.1.16 Ethyl 2-diazo-5-phenyl-3-((trimethylsilyl)oxy)pentanoate (**5h**), $^{13}C\{^1H\}$ NMR spectrum ... 21

2.1.17 Ethyl 2-diazo-3-((trimethylsilyl)oxy)dodecanoate (**5i**), ^1H NMR spectrum ... 22

2.1.18 Ethyl 2-diazo-3-((trimethylsilyl)oxy)dodecanoate (**5i**), $^{13}C\{^1H\}$ NMR spectrum ... 23

2.1.19 Ethyl 2-diazo-5-methyl-3-((trimethylsilyl)oxy)hexanoate (**5j**), ^1H NMR spectrum ... 24

2.1.20 Ethyl 2-diazo-5-methyl-3-((trimethylsilyl)oxy)hexanoate (**5j**), $^{13}C\{^1H\}$ NMR spectrum ... 25

2.1.21 Ethyl 2-diazo-4,4-dimethyl-3-((trimethylsilyl)oxy)pentanoate (**5k**), ^1H NMR spectrum ... 26

2.1.22 Ethyl 2-diazo-4,4-dimethyl-3-((trimethylsilyl)oxy)pentanoate (**5k**), $^{13}C\{^1H\}$ NMR spectrum ... 27

2.1.23 Ethyl 2-diazo-3-(furan-2-yl)-3-((trimethylsilyl)oxy)propanoate (**5l**), ^1H NMR spectrum ... 28

2.1.24 Ethyl 2-diazo-3-(furan-2-yl)-3-((trimethylsilyl)oxy)propanoate (**5l**), $^{13}C\{^1H\}$ NMR spectrum ... 29

2.1.25 *Ethyl 2-diazo-3-((trimethylsilyl)oxy)-3-(5-(((trimethylsilyl)oxy)methyl)furan-2-yl)propanoate* (**5m**), ^1H NMR spectrum ... 30

2.1.26 *Ethyl 2-diazo-3-((trimethylsilyl)oxy)-3-(5-(((trimethylsilyl)oxy)methyl)furan-2-yl)propanoate* (**5m**), $^{13}C\{^1H\}$ NMR spectrum ... 31

2.1.27 *tert*-Butyl (*R*)-3-((*S*)-2-diazo-3-ethoxy-3-oxo-1-((trimethylsilyl)oxy)propyl)-1-oxa-4-azaspiro[4.5]decane-4-carboxylate (**5n**), ^1H NMR spectrum ... 32

2.1.28 *tert*-Butyl (*R*)-3-((*S*)-2-diazo-3-ethoxy-3-oxo-1-((trimethylsilyl)oxy)propyl)-1-oxa-4-azaspiro[4.5]decane-4-carboxylate (**5n**), $^{13}C\{^1H\}$ NMR spectrum ... 33

2.1.29 *tert*-Butyl (*R*)-3-((*R*)-2-diazo-3-ethoxy-1-hydroxy-3-oxopropyl)-1-oxa-4-azaspiro[4.5]decane-4-carboxylate (**6**), ^1H NMR spectrum ... 34

2.1.30 *tert*-Butyl (*R*)-3-((*R*)-2-diazo-3-ethoxy-1-hydroxy-3-oxopropyl)-1-oxa-4-azaspiro[4.5]decane-4-carboxylate (**6**), $^{13}C\{^1H\}$ NMR spectrum ... 35

2.1.31 *tert*-Butyl (*R*)-3-((*S*)-3-ethoxy-3-oxo-1-((trimethylsilyl)oxy)propyl)-1-oxa-4-azaspiro[4.5]decane-4-carboxylate (**7**), ^1H NMR spectrum ... 36

2.1.32 *tert*-Butyl (*R*)-3-((*S*)-3-ethoxy-3-oxo-1-((trimethylsilyl)oxy)propyl)-1-oxa-4-azaspiro[4.5]decane-4-carboxylate (**7**), $^{13}C\{^{1}H\}$ NMR spectrum 37

2.1.33 *tert*-Butyl (*R*)-3-((*S*)-3-ethoxy-1-hydroxy-3-oxopropyl)-1-oxa-4-azaspiro[4.5]decane-4-carboxylate (**8**), ^{1}H NMR spectrum 38

2.1.34 *tert*-Butyl (*R*)-3-((*S*)-3-ethoxy-1-hydroxy-3-oxopropyl)-1-oxa-4-azaspiro[4.5]decane-4-carboxylate (**8**), $^{13}C\{^{1}H\}$ NMR spectrum 39

2.1.35 Ethyl 2-((*4S,5R*)-5-((*tert*-butoxycarbonyl)amino)-2,2-dimethyl-1,3-dioxan-4-yl)acetate (**9a**), ^{1}H NMR spectrum 40

2.1.36 Ethyl 2-((*4S,5R*)-5-((*tert*-butoxycarbonyl)amino)-2,2-dimethyl-1,3-dioxan-4-yl)acetate (**9a**), $^{13}C\{^{1}H\}$ NMR spectrum 41

2.1.37 Ethyl 2-((*2S,3R*)-3-((*tert*-butoxycarbonyl)amino)-1,5-dioxaspiro[5.5]undecan-2-yl)acetate (**9b**), ^{1}H NMR spectrum 42

2.1.38 Ethyl 2-((*2S,3R*)-3-((*tert*-butoxycarbonyl)amino)-1,5-dioxaspiro[5.5]undecan-2-yl)acetate (**9b**), $^{13}C\{^{1}H\}$ NMR spectrum 43

2.1.39 *tert*-Butyl 3-(2-diazo-3-ethoxy-3-oxo-1-((trimethylsilyl)oxy)propyl)-1-oxa-4-azaspiro[4.5]decane-4-carboxylate (***(±)*-5n**), ^{1}H NMR spectrum 44

2.1.40 *tert*-Butyl 3-(2-diazo-3-ethoxy-3-oxo-1-((trimethylsilyl)oxy)propyl)-1-oxa-4-azaspiro[4.5]decane-4-carboxylate (***(±)*-5n**), $^{13}C\{^{1}H\}$ NMR spectrum 45

2.1.41 *tert*-Butyl 3-(2-diazo-3-ethoxy-1-hydroxy-3-oxopropyl)-1-oxa-4-azaspiro[4.5]decane-4-carboxylate (***(±)*-6**), ^{1}H NMR spectrum 46

2.1.42 *tert*-Butyl 3-(2-diazo-3-ethoxy-1-hydroxy-3-oxopropyl)-1-oxa-4-azaspiro[4.5]decane-4-carboxylate (***(±)*-6**), $^{13}C\{^{1}H\}$ NMR spectrum 47

2.2 HPLC measurements 48

2.2.1 HPLC chromatogram **(±)-6**, racemic mixture from a reaction carried out at RT (internal code OIC-441) 48

2.2.2 HPLC chromatogram (***R,R***)**-6** from a reaction carried out at 0 ^{0}C (internal code OIC-448) 49

2.2.3 HPLC chromatogram (***R,R***)**-6** from a reaction carried out at RT (internal code OIC-447) 50

2.3 ^{1}H NMR control experiments 51

2.3.1 Control experiment without TMAP (internal code OIC-443) 51

2.3.2 TMS- ethyl diazoacetate formation (internal code OIC-444) 52

2.3.3 Reaction rate determination with BSA (internal code OIC-451) 53

2.3.4 Reaction rate determination with BSTFA (internal code OIC-452) 54

2.4 ^{1}H NMR competition experiments 55

2.4.1 Competition experiment with **1a** and **1c** (internal code TFN-4-110) 55

2.4.2 Competition experiment with **1a** and **1d** (internal code TFN-4-112) 57

2.4.3 Competition experiment with **1a** and **1f** (internal code TFN-4-114) 59

2.5 ^{1}H NMR cross-over experiments 61

2.5.1 Cross-over experiment with **1b** and **4a** in the presence of BSA (internal code OIC-456) 61

2.5.2 Cross-over experiment with **1b** and **4a** without BSA (internal code OIC-457) 64

1 General Information

All reactions were carried out under an argon atmosphere in oven-dried glassware, unless otherwise noted. When needed, nonaqueous reagents were transferred under argon *via* syringe or cannula and dried prior to use. Dry deoxygenated MeCN and CH_2Cl_2 were obtained by passing deoxygenated solvents through activated alumina columns (MBraun SPS-800 Series solvent purification system). Other solvents and reagents were used as obtained from supplier, unless otherwise noted. Analytical TLC was performed using Merck silica gel F254 (230-400 mesh) plates and analyzed by UV light or by staining upon heating with $KMnO_4$ solution (1 g $KMnO_4$, 6.7 g K_2CO_3, 1.7 mL 1M NaOH, 100 mL H_2O). For gel chromatography, the flash chromatography technique was used, with Merck silica gel 60 (230-400 mesh) and p.a. grade solvents unless otherwise noted. For automated flash chromatography Teledyne Isco CombiFlash Rf 200 apparatus and RediSep® Silver columns were used.

The ^1H NMR and ^{13}C NMR spectra were recorded in either $CDCl_3$, CD_3CN or DMSO-*d3* on Bruker Avance 500, 400 or 300 MHz spectrometers. The chemical shifts are reported in ppm relative to $CHCl_3$ (δ 7.26), CHD_2CN (δ 1.94) or ^1H NMR. For the ^{13}C NMR spectra, the residual $CDCl_3$ (δ 77.16), CD_3CN (δ 118.26) and were used as the internal standards. IR spectra were recorded on a Tensor27 FT-IR spectrometer. Optical rotations were obtained with a Perkin-Elmer 343 polarimeter. High resolution mass spectrometric data were measured using Agilent 6560 IM-QTOF mass spectrometer. The enantiomeric (*er*) and diastereomeric (*dr*) ratio of the products were determined by HPLC using Waters 501 pump and Waters 486 detector in comparison to the corresponding racemic sample. CHIRALCEL® OZ-H from Daicel. Samples with 1mg/ml concentration in hexane were measured using 99/1 = Hexane/*i*-PrOH (IPA) as an eluent, flow rate of 1 ml/min, and injection volume of 15 μl was used in the measurements.

Note: In ^{13}C spectra carbon atom next to the diazo group was not observed due to quadrupolar relaxation.

2 NMR Spectra, HPLC Chromatograms, and Control Experiments

2.1.1 Ethyl 2-diazo-3-phenyl-3-((trimethylsilyl)oxy)propanoate (**5a**), ^{1}H NMR spectrum

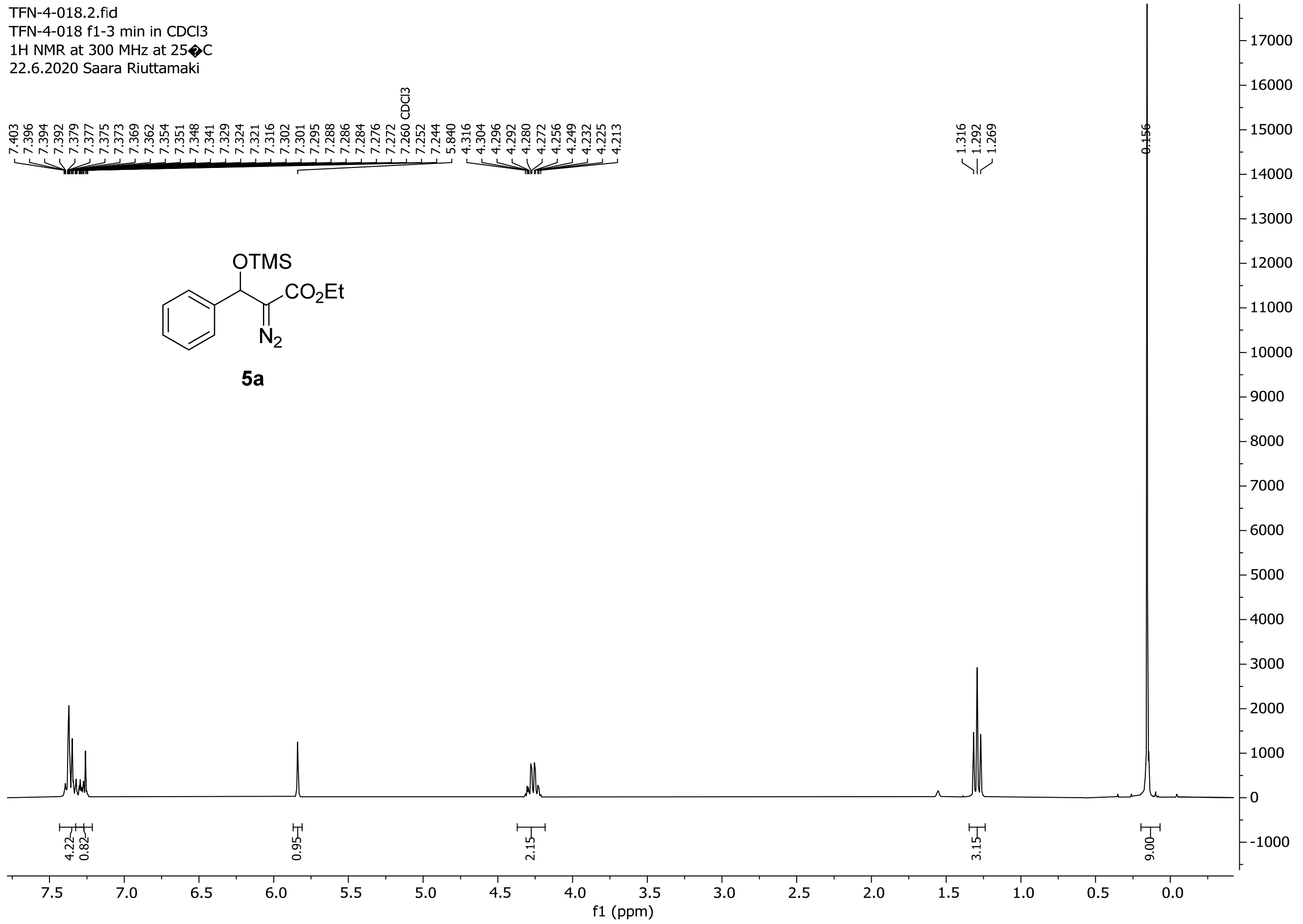

2.1.2 Ethyl 2-diazo-3-phenyl-3-((trimethylsilyl)oxy)propanoate (**5a**), $^{13}C\{^{1}H\}$ NMR spectrum

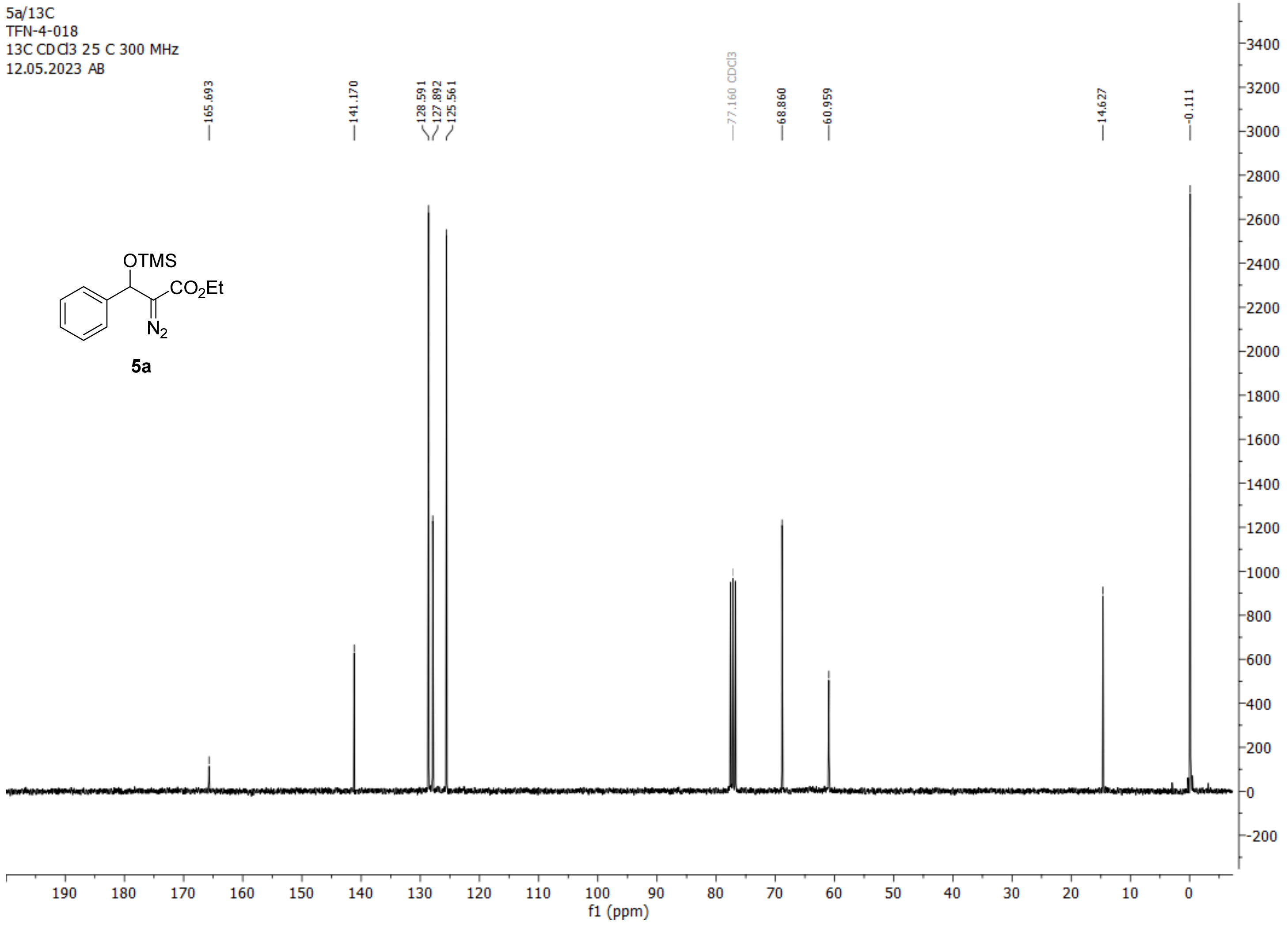

2.1.3 Ethyl 2-diazo-3-(4-(trifluoromethyl)phenyl)-3-((trimethylsilyl)oxy)propanoate (**5b**), ^{1}H NMR spectrum

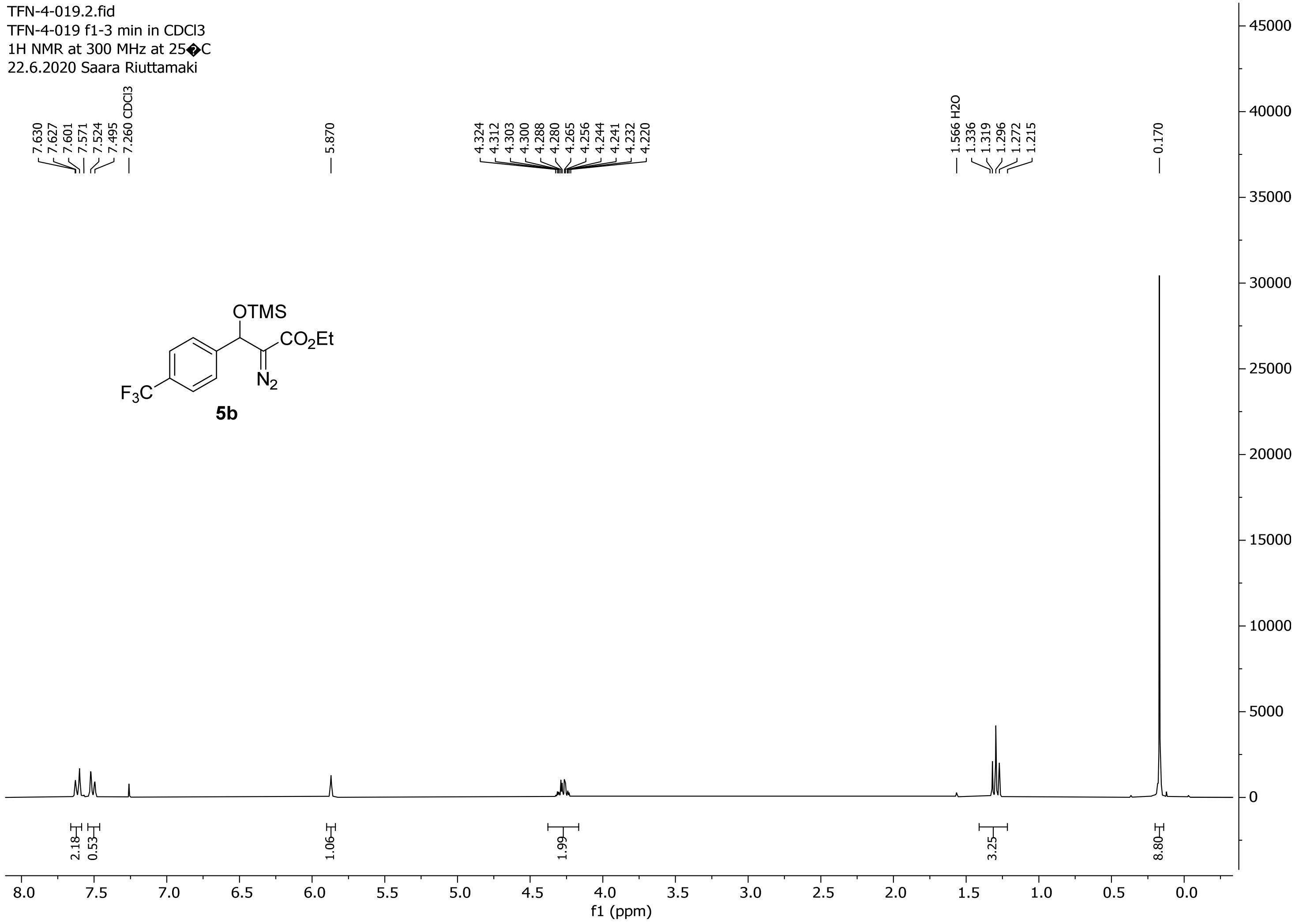

2.1.4 Ethyl 2-diazo-3-(4-(trifluoromethyl)phenyl)-3-((trimethylsilyl)oxy)pro panoate (**5b**), $^{13}C\{^{1}H\}$ NMR spectrum

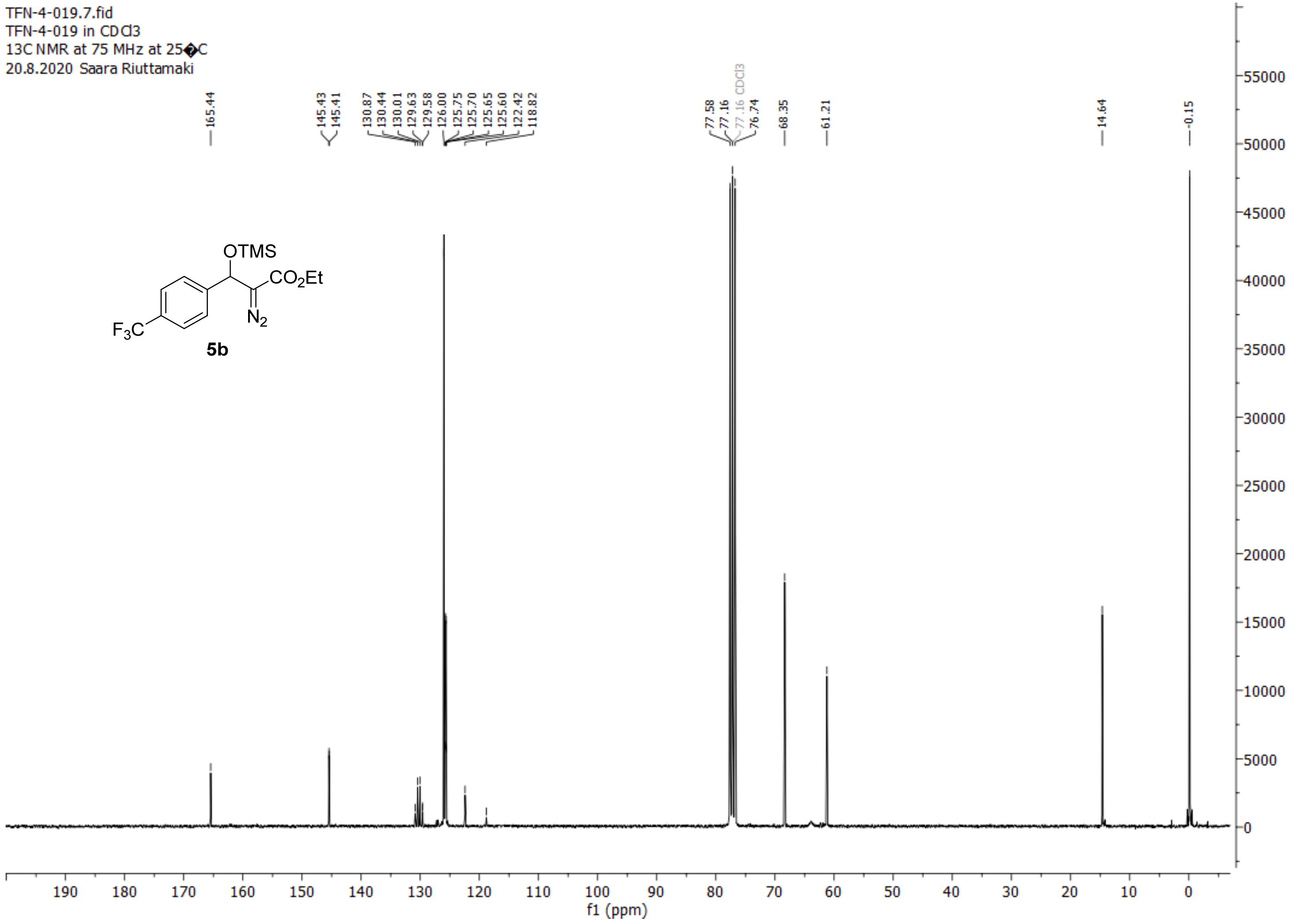

2.1.5 Ethyl 2-diazo-3-(4-methoxyphenyl)-3-((trimethylsilyl)oxy)propanoate (**5c**), ^{1}H NMR spectrum

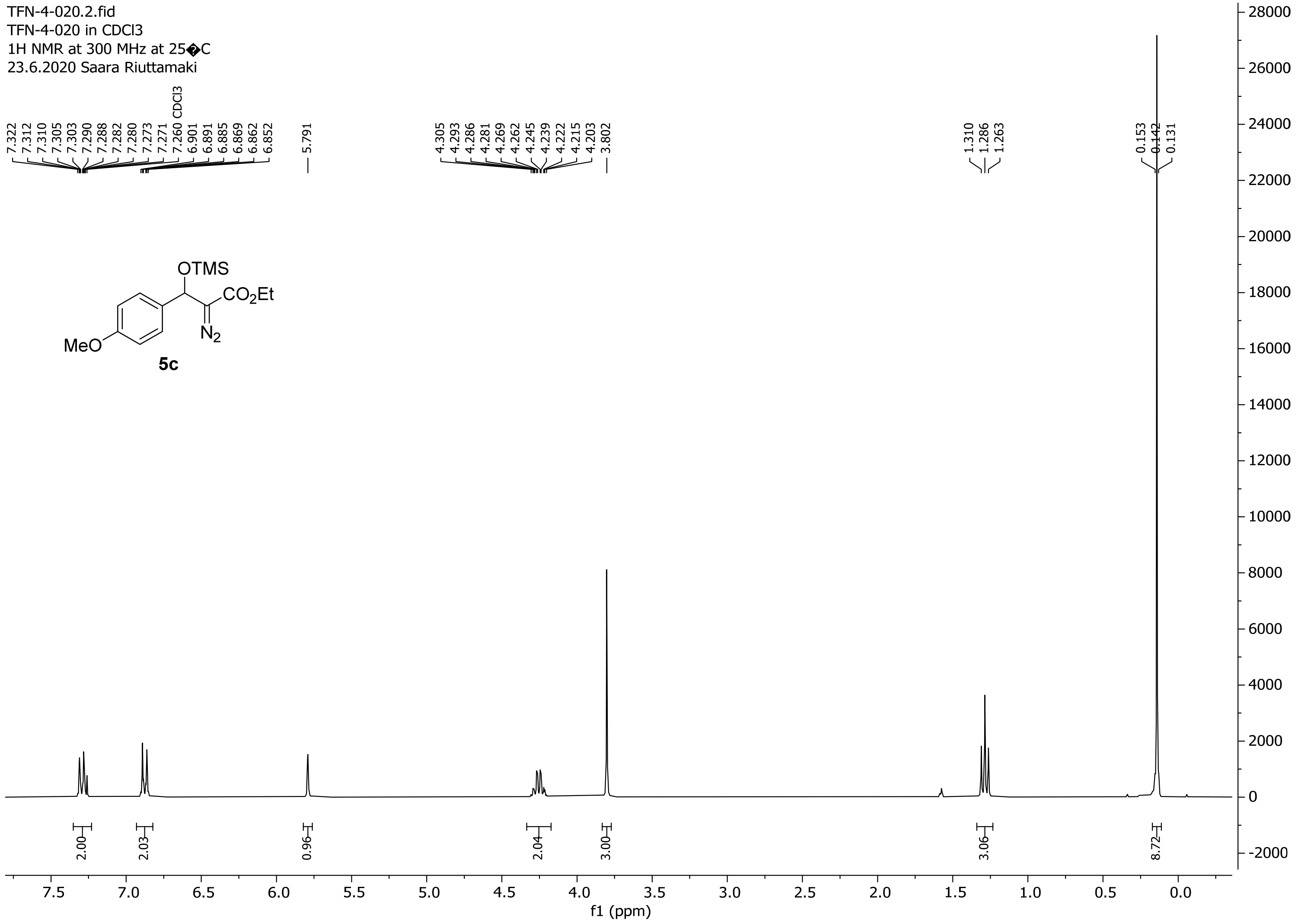

2.1.6 Ethyl 2-diazo-3-(4-methoxyphenyl)-3-((trimethylsilyl)oxy)propanoate, (**5c**), $^{13}C\{^{1}H\}$ NMR spectrum

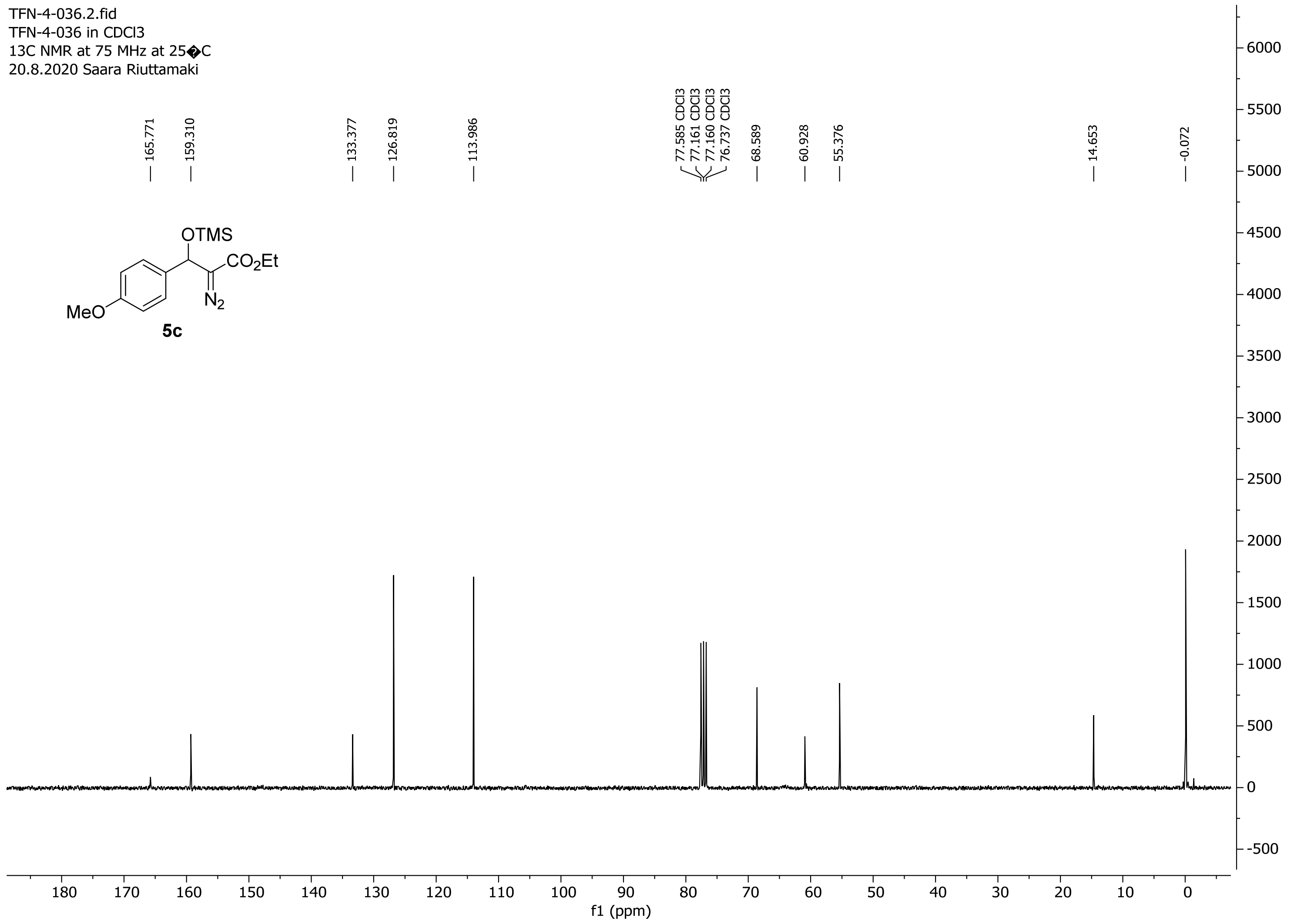

2.1.7 Ethyl 2-diazo-3-(p-tolyl)-3-((trimethylsilyl)oxy)propanoate (**5d**), ^{1}H NMR spectrum

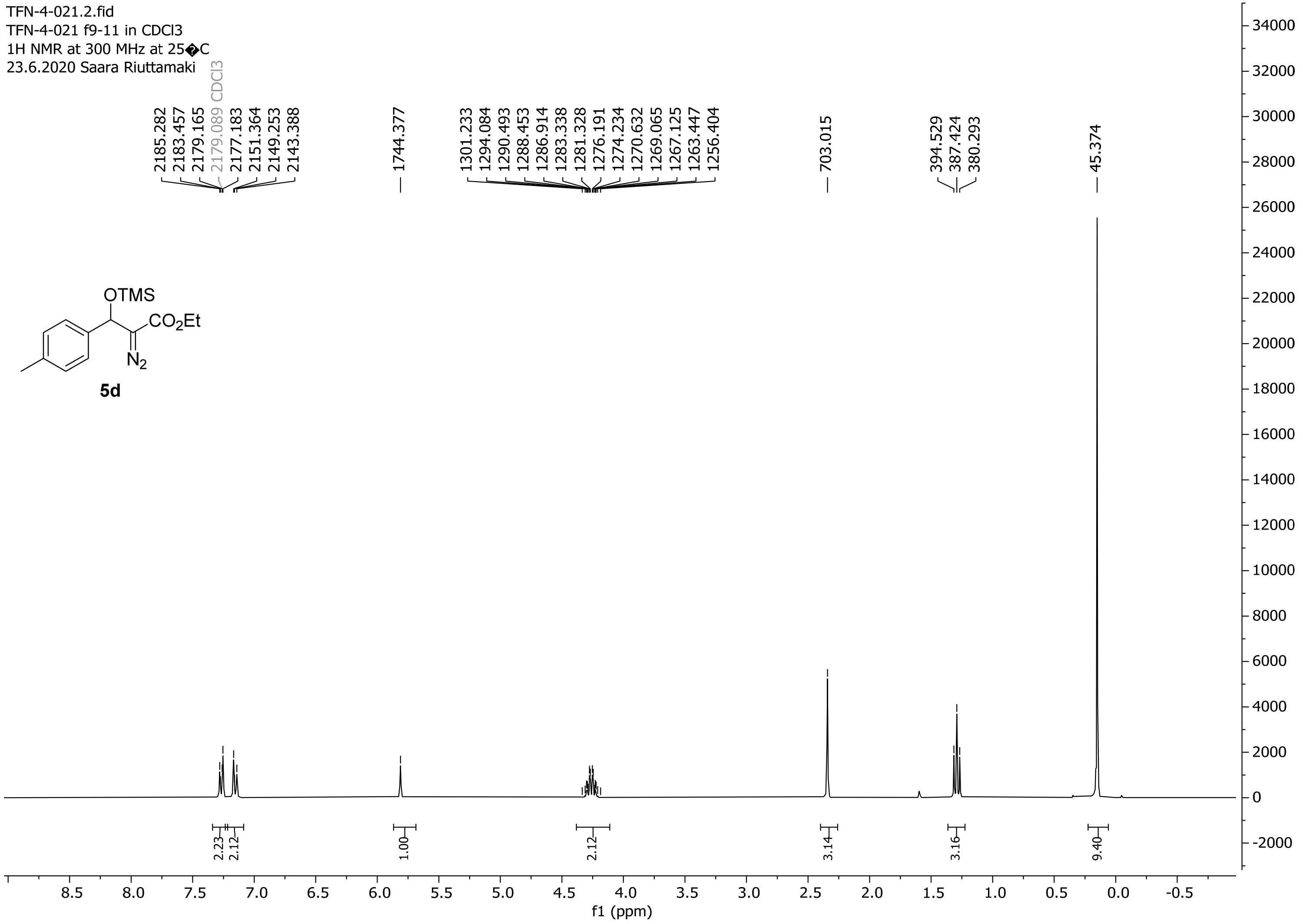

2.1.8 Ethyl 2-diazo-3-(p-tolyl)-3-((trimethylsilyl)oxy)propanoate (**5d**), $^{13}C\{^{1}H\}$ NMR spectrum

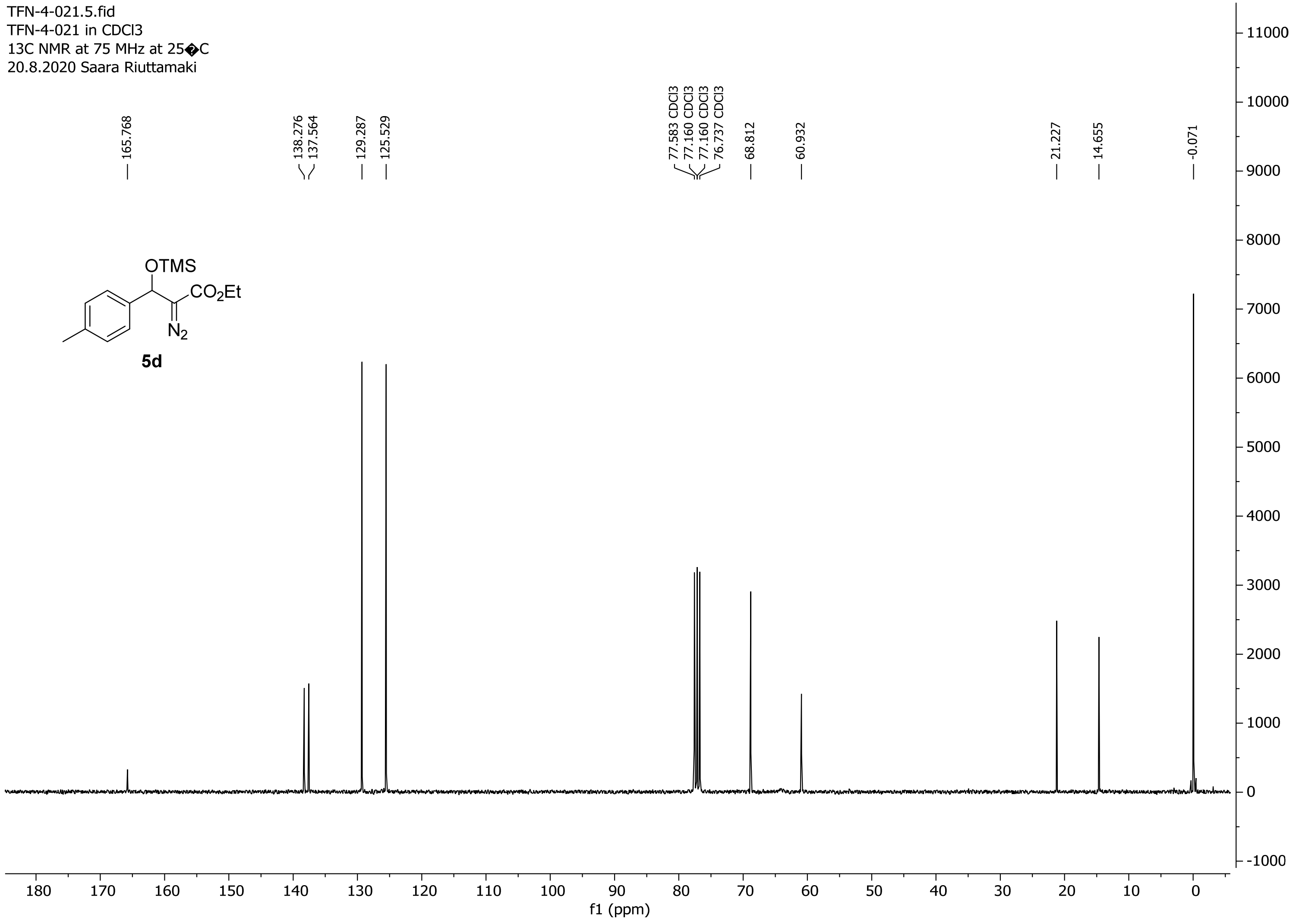

2.1.9 Ethyl 3-(3-bromophenyl)-2-diazo-3-((trimethylsilyl)oxy)propanoate (**5e**), ^{1}H NMR spectrum

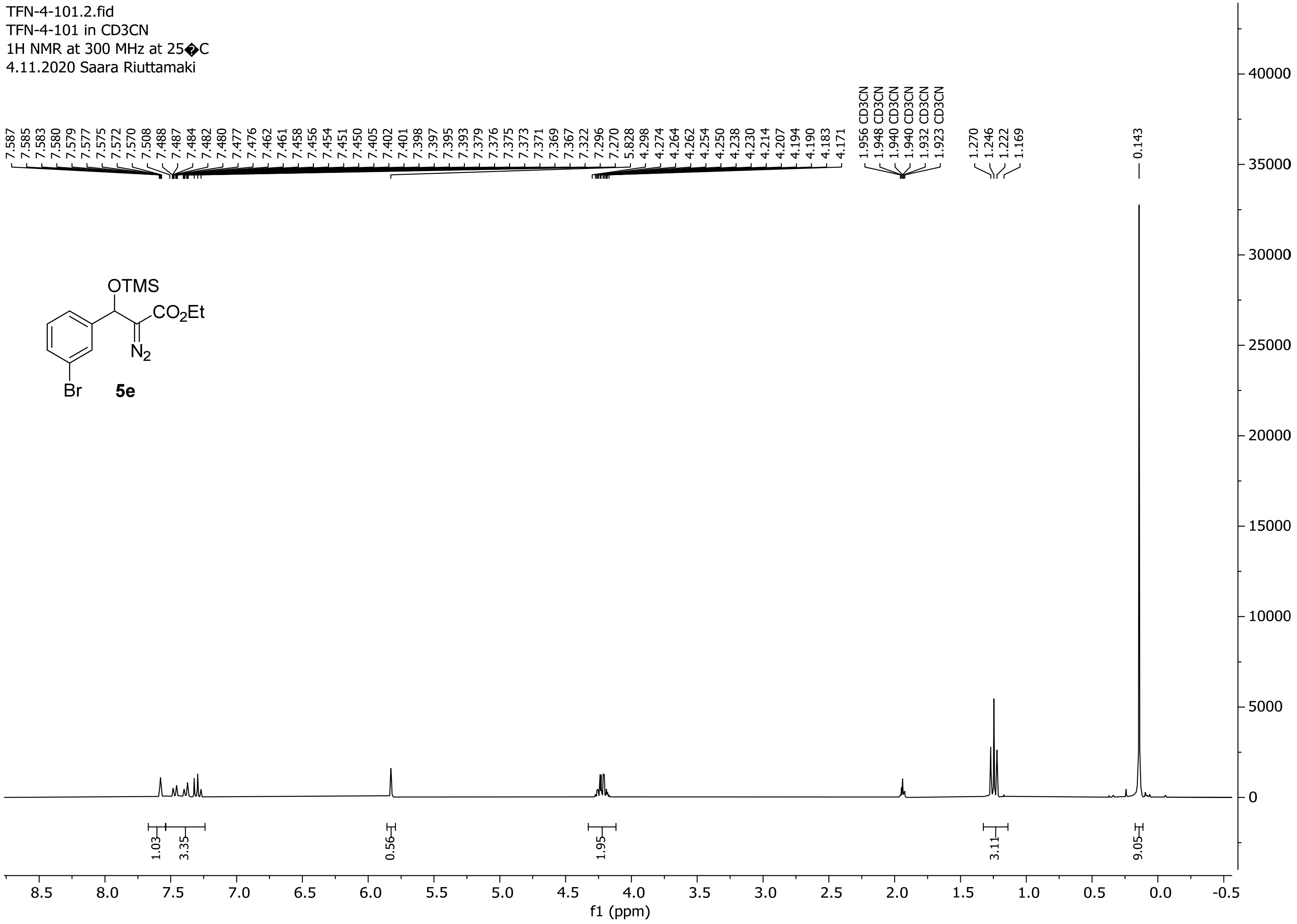

2.1.10 Ethyl 3-(3-bromophenyl)-2-diazo-3-((trimethylsilyl)oxy)propanoate (**5e**), $^{13}C\{^1H\}$ NMR spectrum

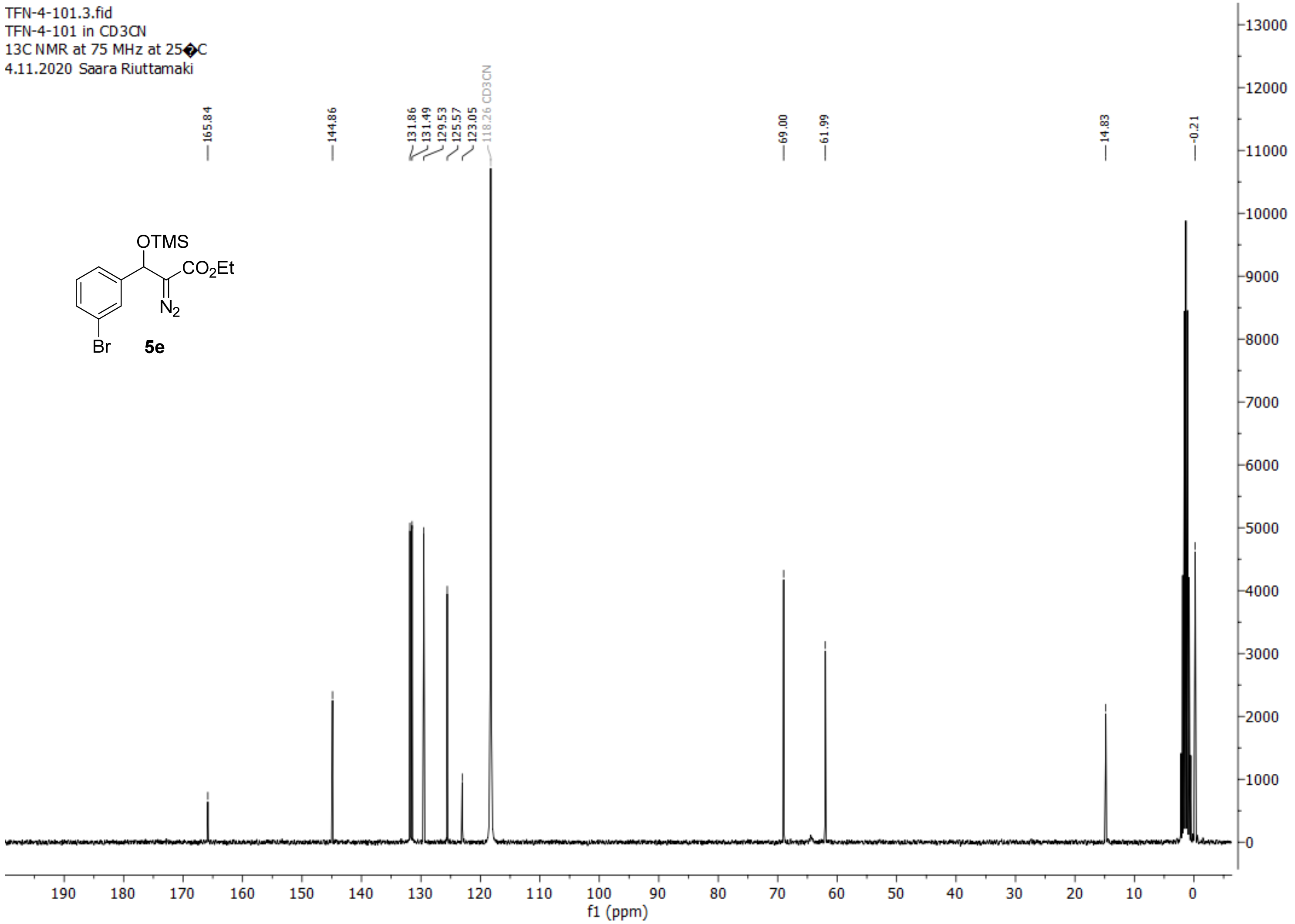

2.1.11 Ethyl 3-(4-chlorophenyl)-2-diazo-3-((trimethylsilyl)oxy)propanoate (**5f**), ^{1}H NMR spectrum

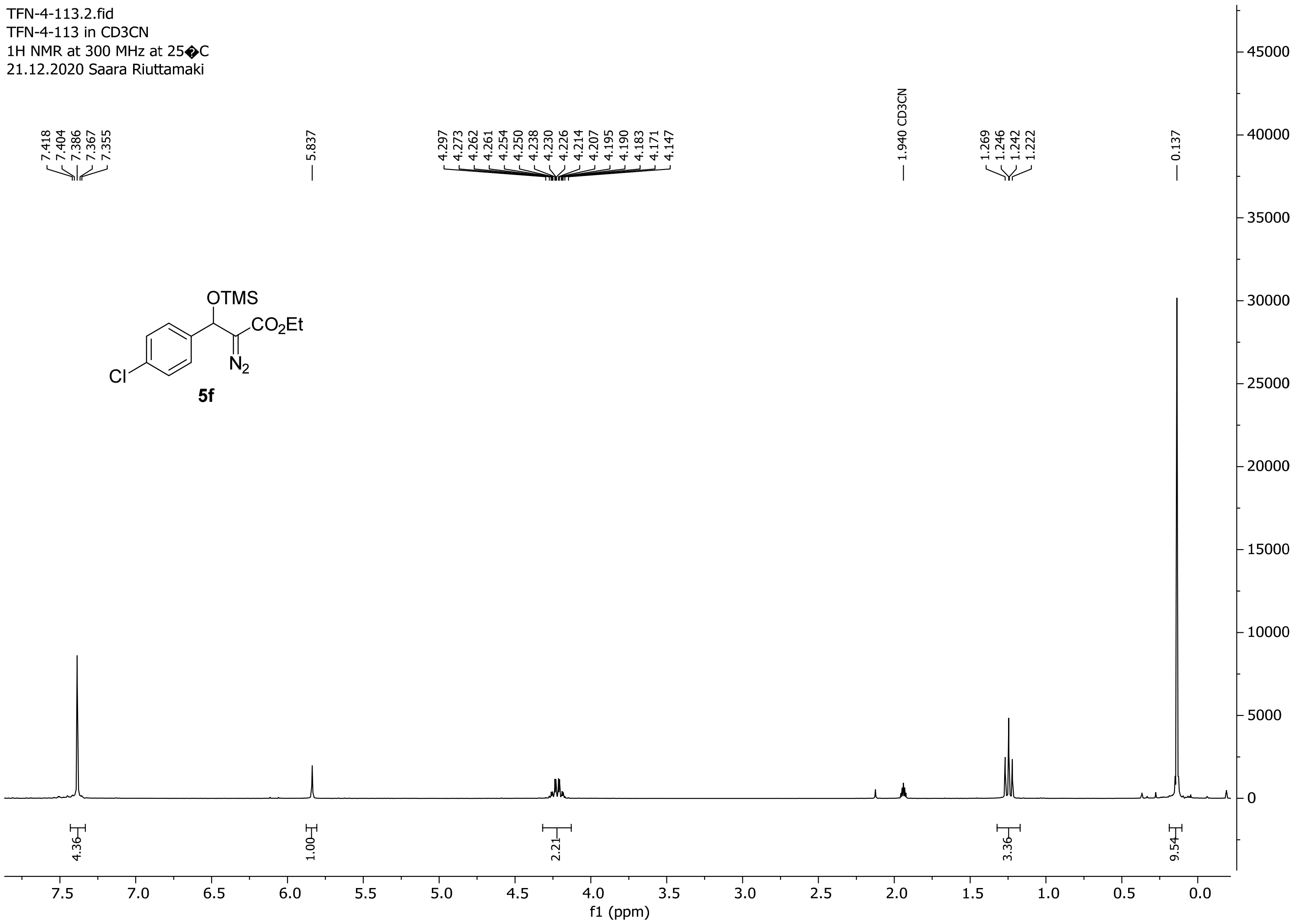

2.1.12 Ethyl 3-(4-chlorophenyl)-2-diazo-3-((trimethylsilyl)oxy)propanoate (**5f**), $^{13}C\{^{1}H\}$ NMR spectrum

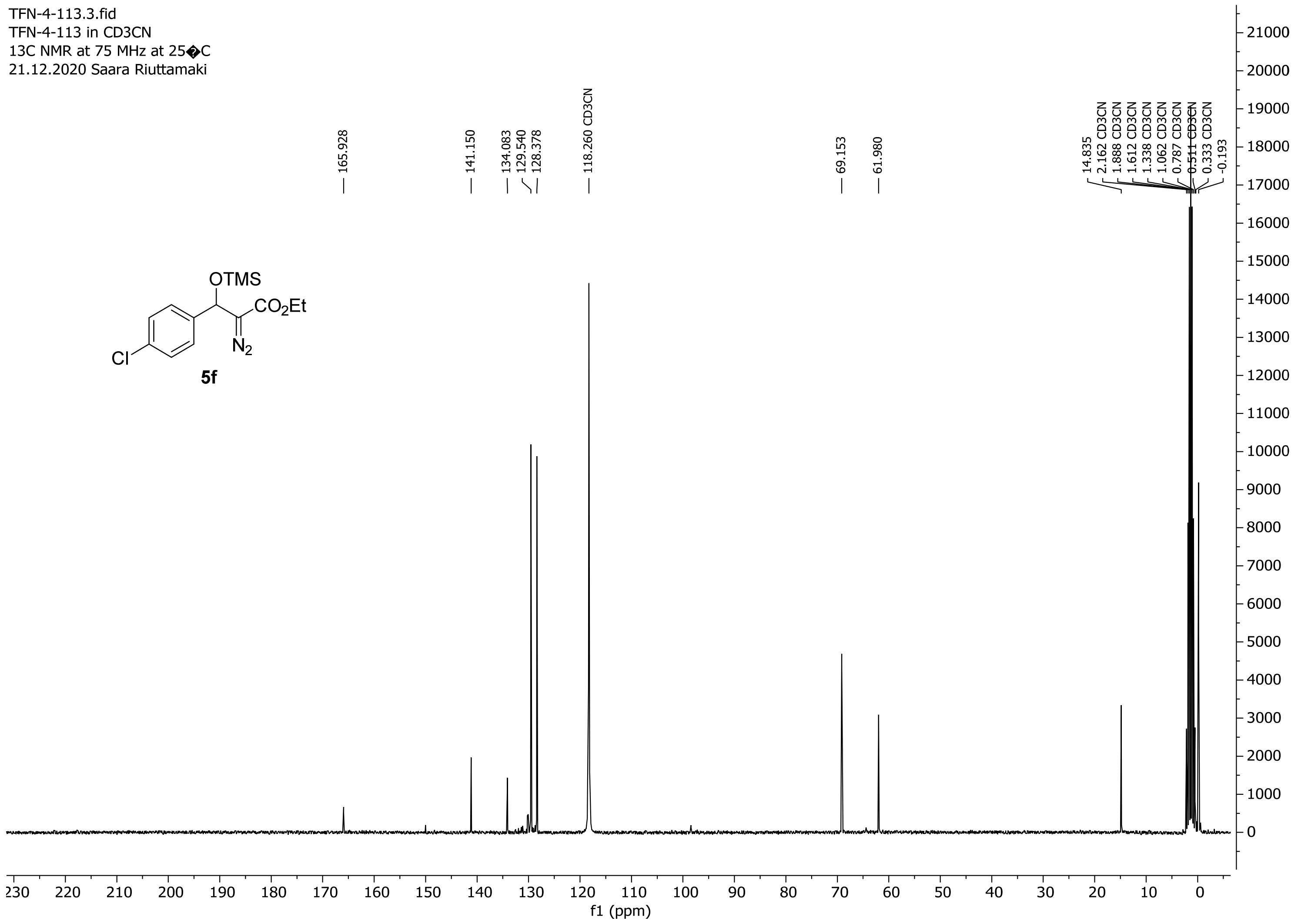

2.1.13 Ethyl 2-diazo-3-((trimethylsilyl)oxy)-3-(4-((trimethylsilyl)oxy)phenyl)propanoate (**5g**), ^{1}H NMR spectrum

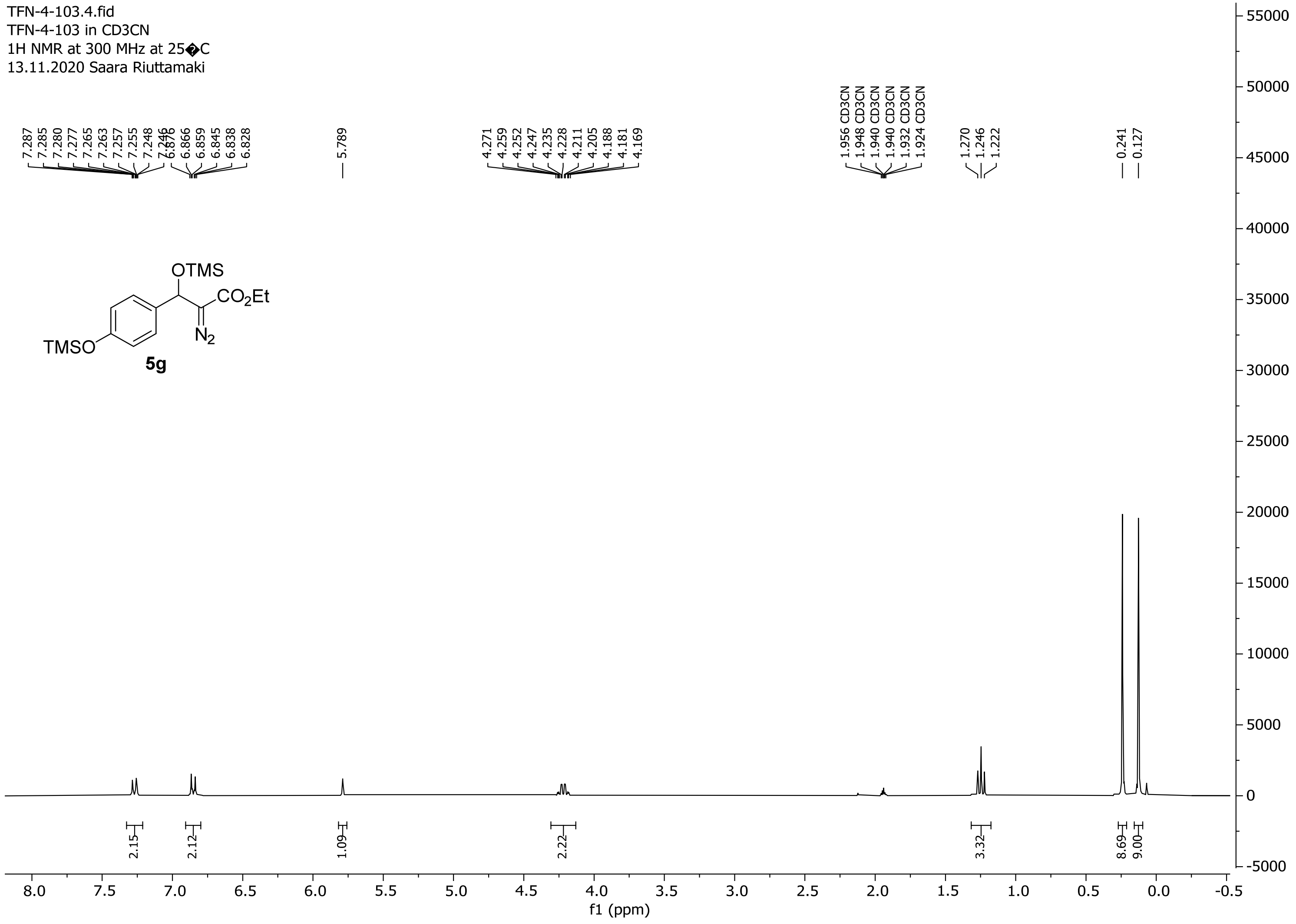

2.1.14 Ethyl 2-diazo-3-((trimethylsilyl)oxy)-3-(4-((trimethylsilyl)oxy)phenyl)propanoate (**5g**), $^{13}C\{^{1}H\}$ NMR spectrum

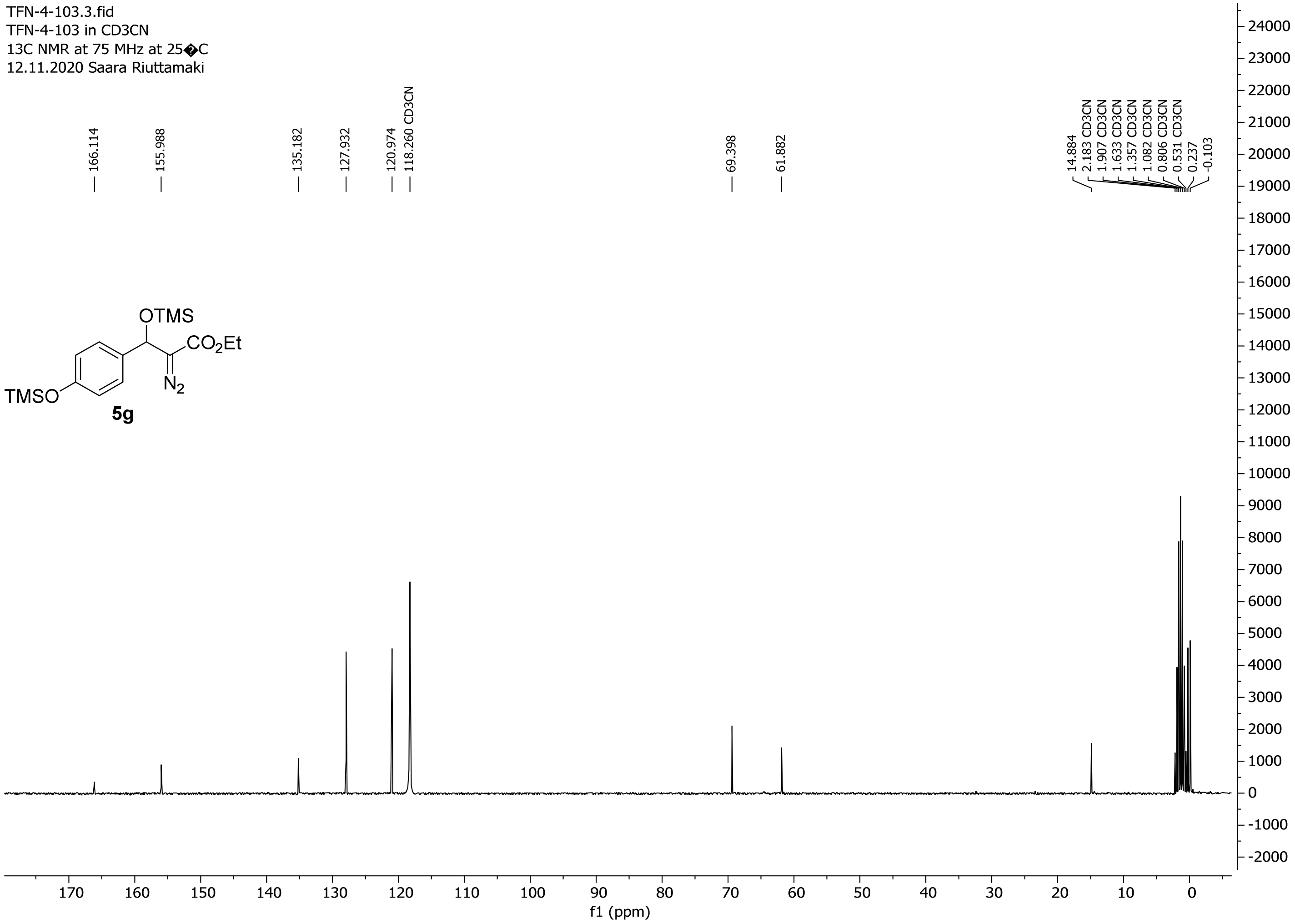

2.1.15 Ethyl 2-diazo-5-phenyl-3-((trimethylsilyl)oxy)pentanoate (**5h**), ^{1}H NMR spectrum

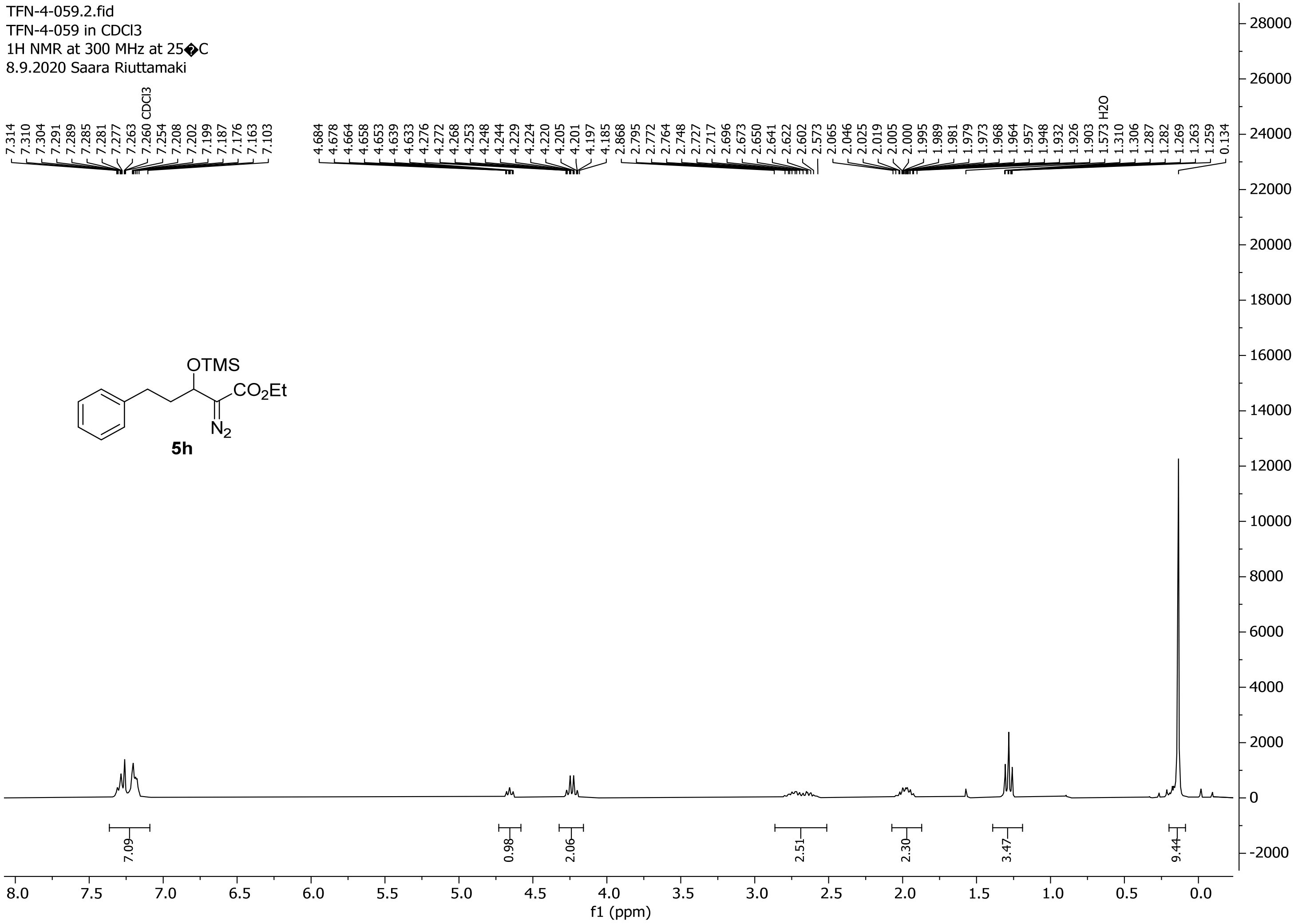

2.1.16 Ethyl 2-diazo-5-phenyl-3-((trimethylsilyl)oxy)pentanoate (**5h**), $^{13}C\{^1H\}$ NMR spectrum

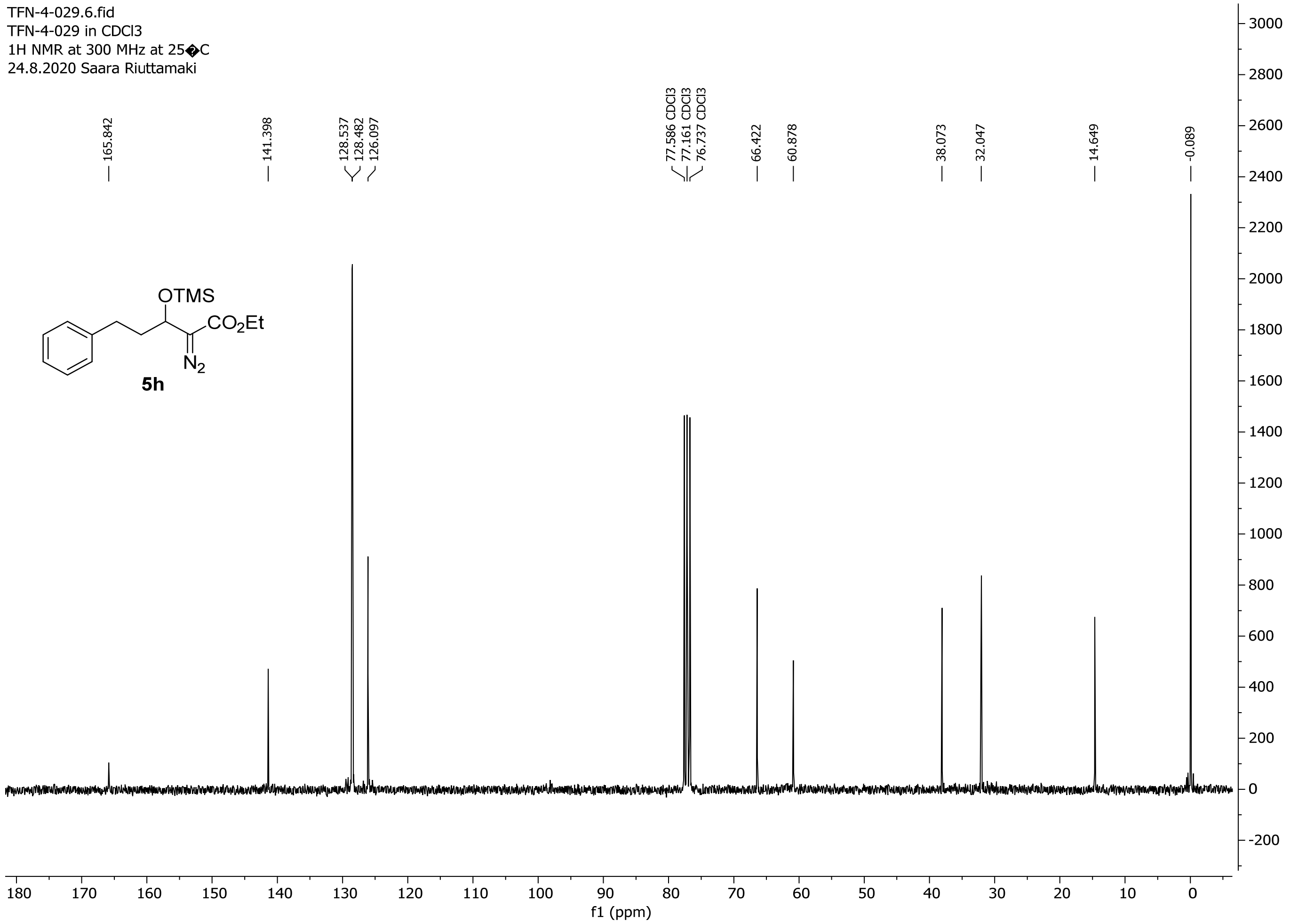

2.1.17 Ethyl 2-diazo-3-((trimethylsilyl)oxy)dodecanoate (**5i**), ^{1}H NMR spectrum

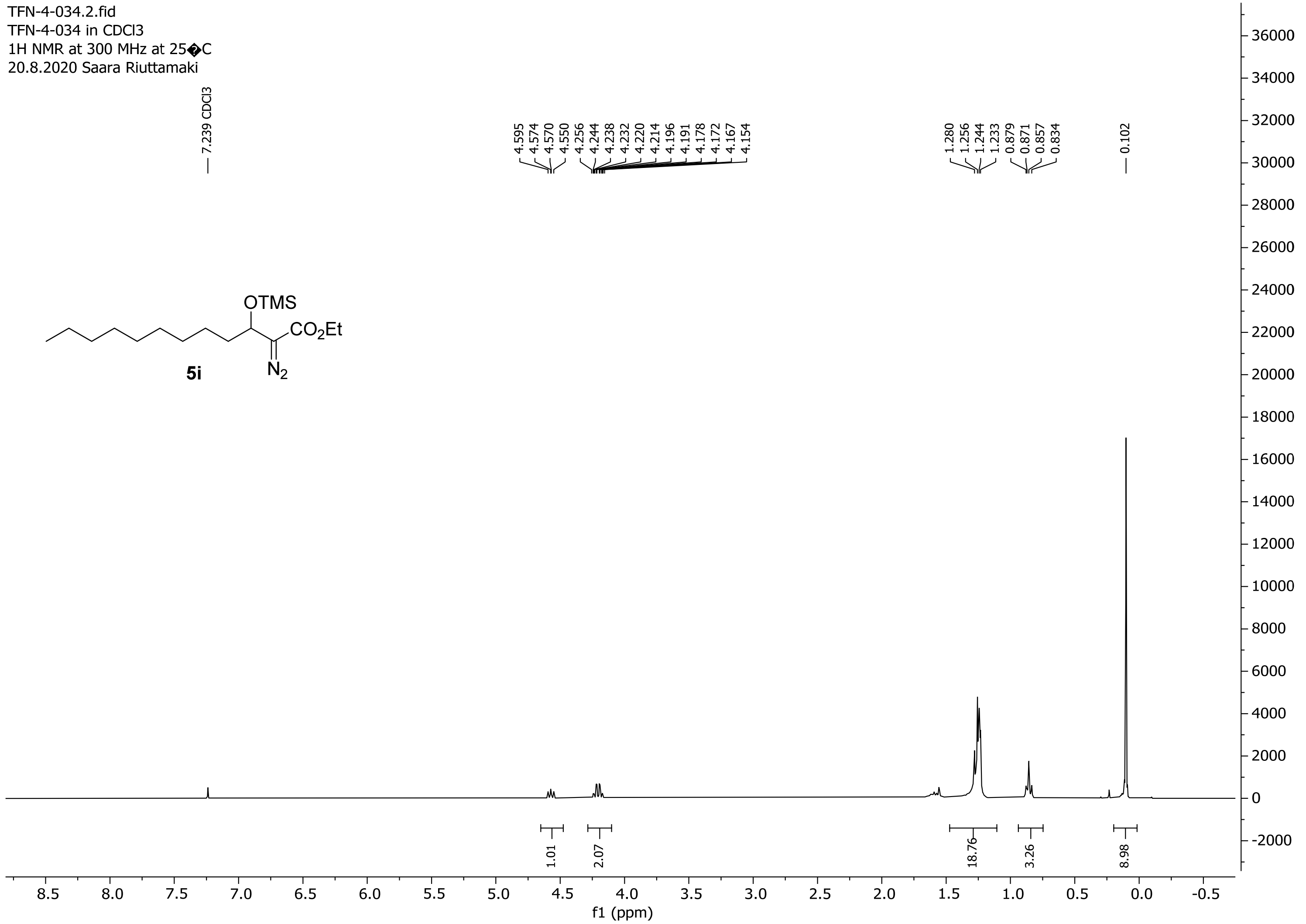

2.1.18 Ethyl 2-diazo-3-((trimethylsilyl)oxy)dodecanoate (**5i**), $^{13}C\{^{1}H\}$ NMR spectrum

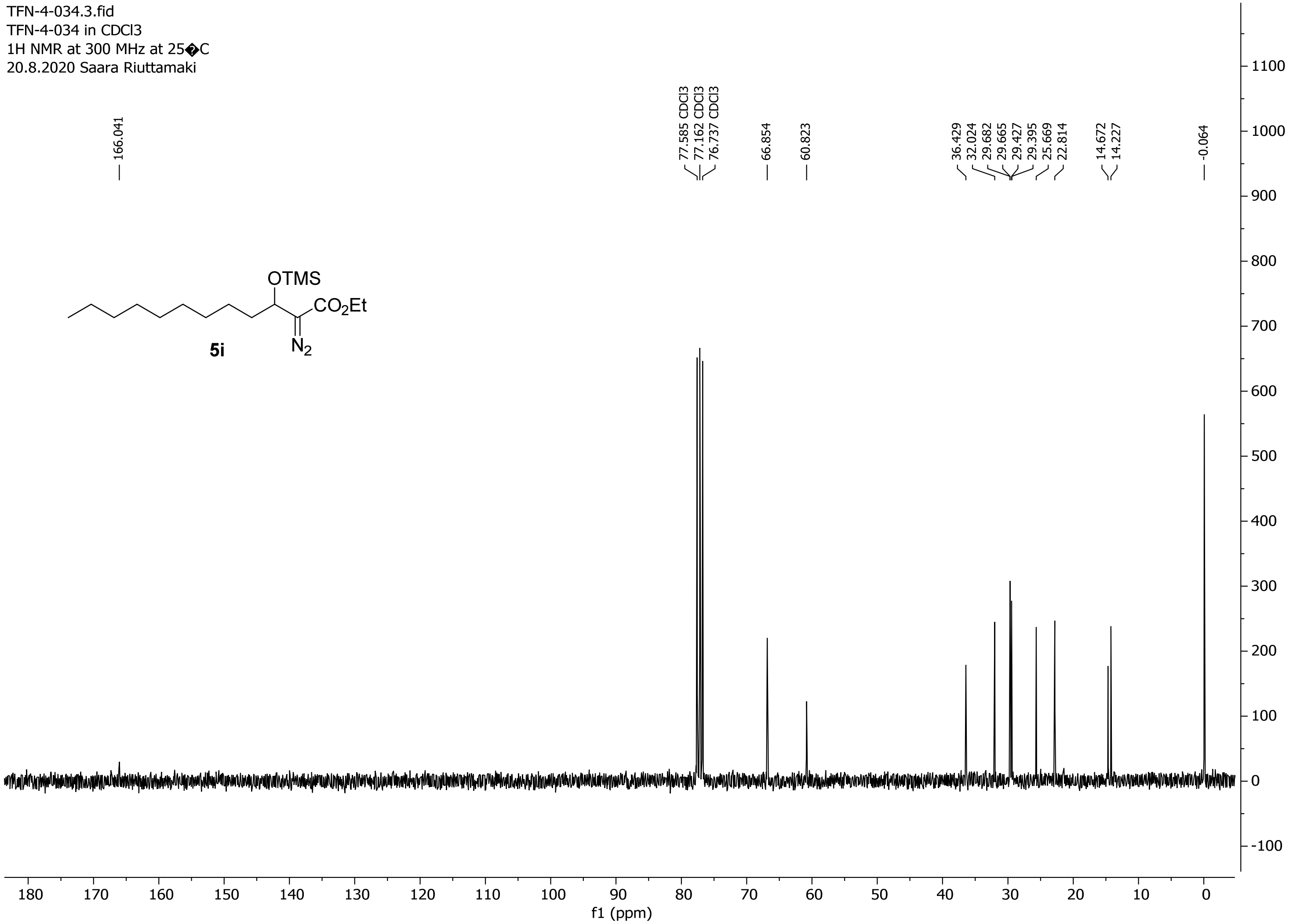

2.1.19 Ethyl 2-diazo-5-methyl-3-((trimethylsilyl)oxy)hexanoate (**5j**), ^{1}H NMR spectrum

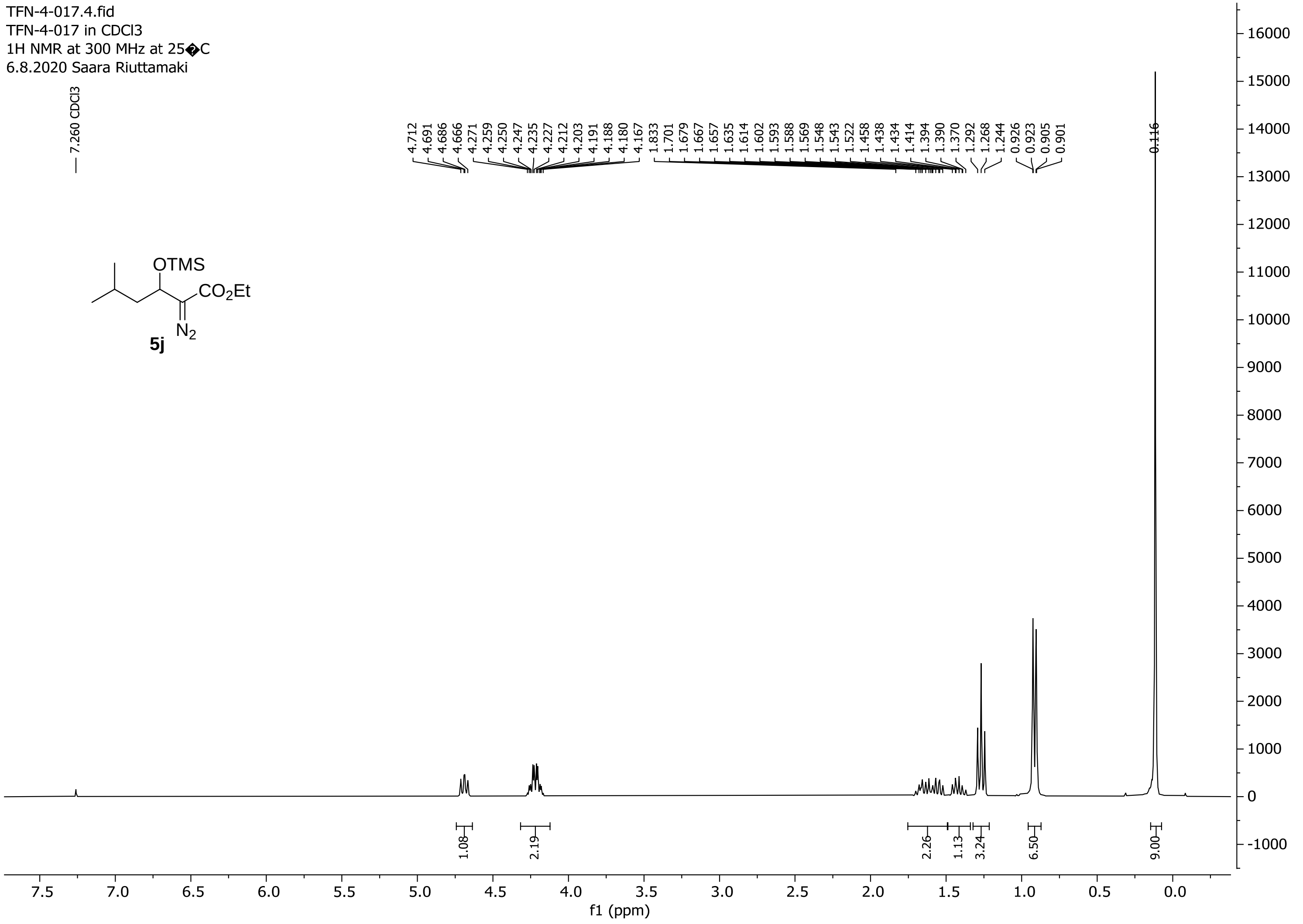

2.1.20 Ethyl 2-diazo-5-methyl-3-((trimethylsilyl)oxy)hexanoate (**5j**), $^{13}C\{^{1}H\}$ NMR spectrum

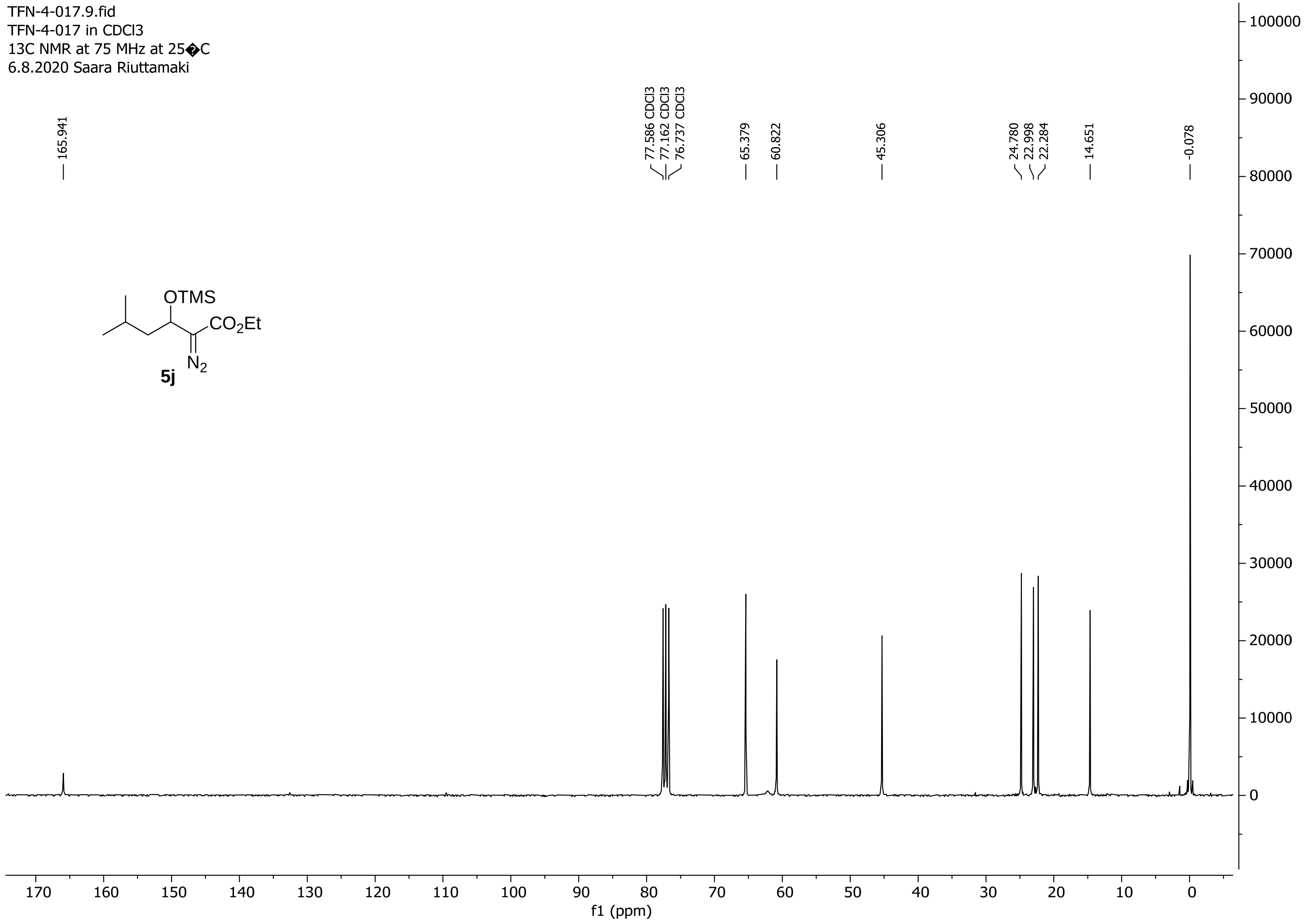

2.1.21 Ethyl 2-diazo-4,4-dimethyl-3-((trimethylsilyl)oxy)pentanoate (**5k**), ^{1}H NMR spectrum

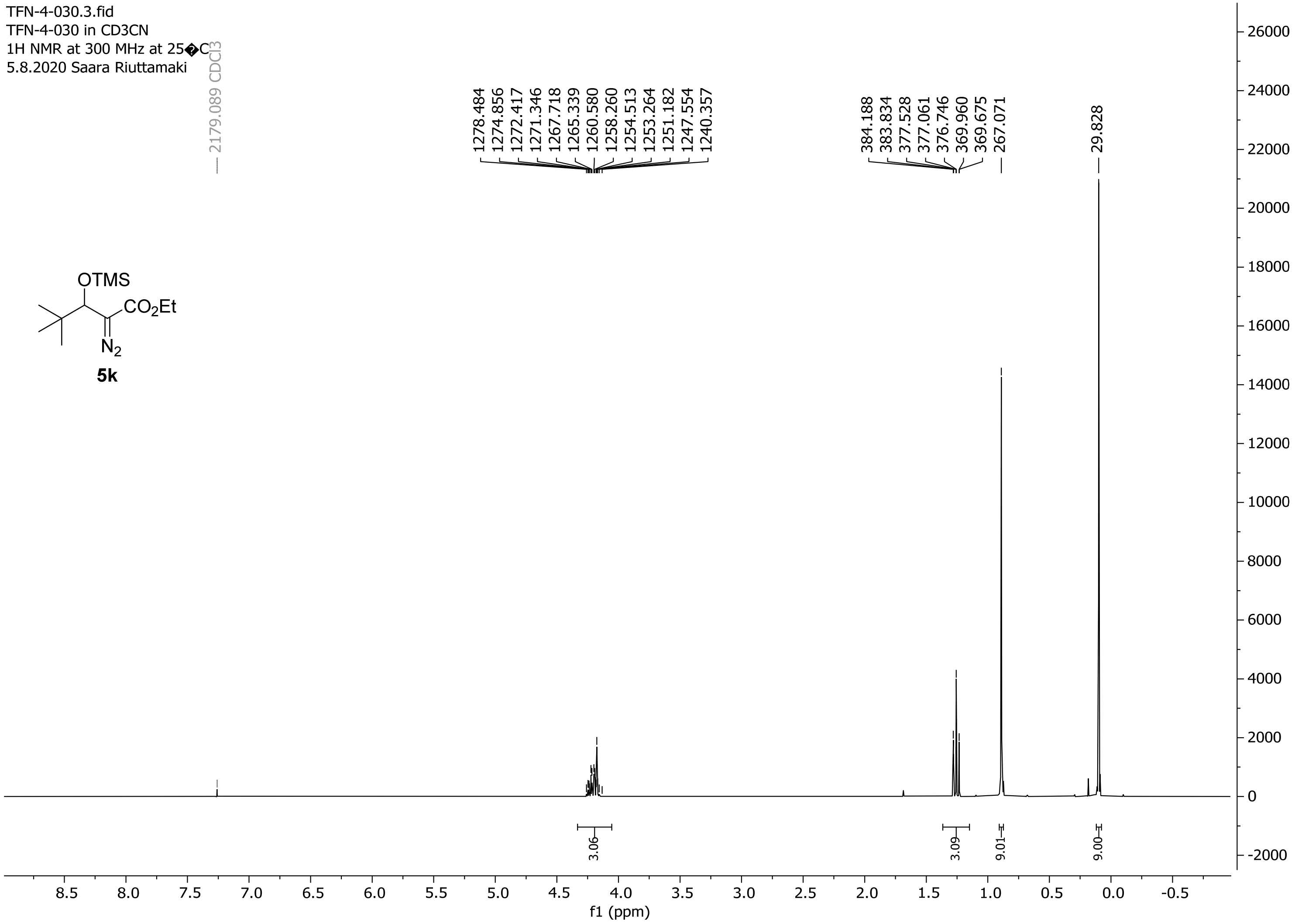

2.1.22 Ethyl 2-diazo-4,4-dimethyl-3-((trimethylsilyl)oxy)pentanoate (**5k**), $^{13}C\{^{1}H\}$ NMR spectrum

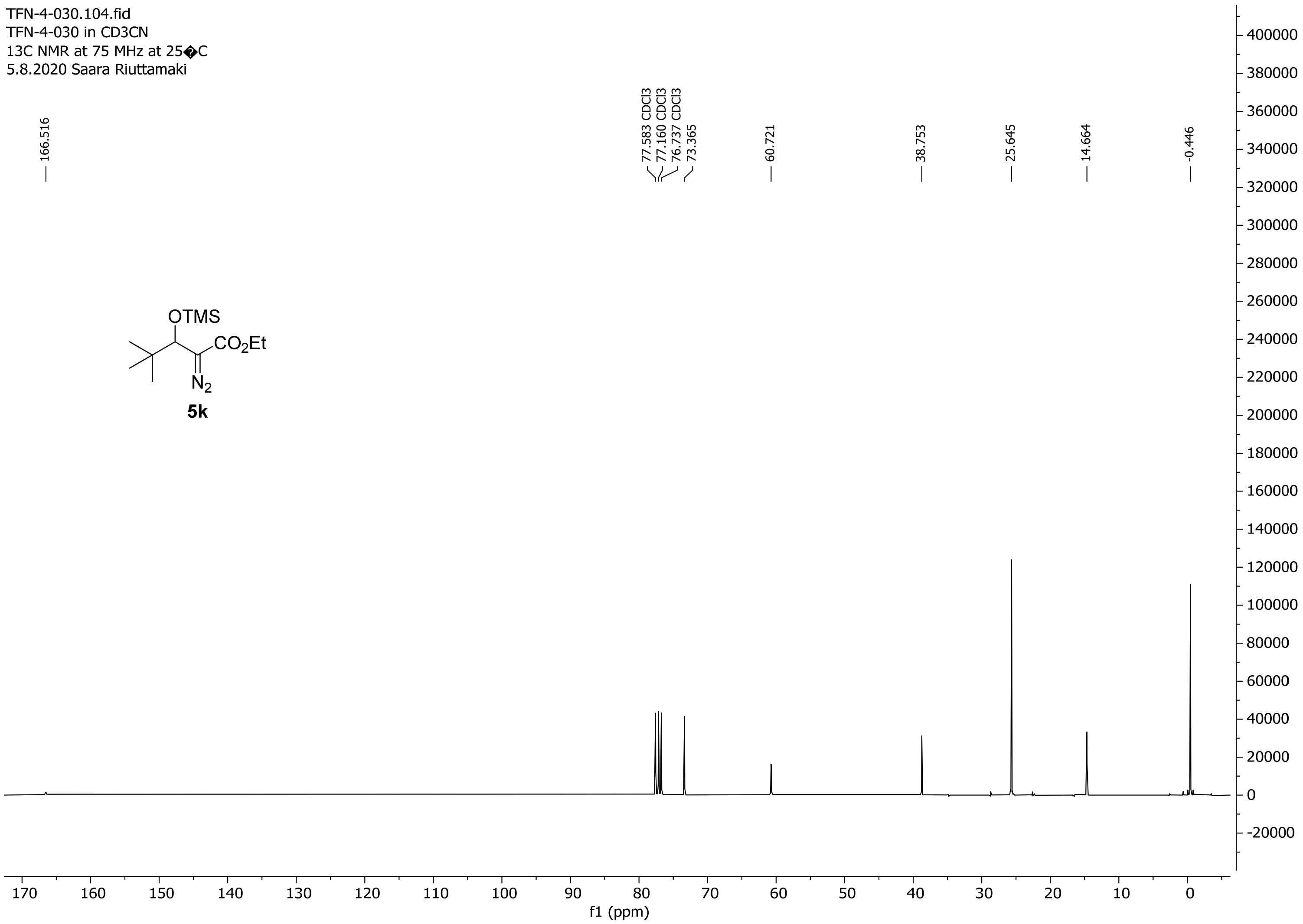

2.1.23 Ethyl 2-diazo-3-(furan-2-yl)-3-((trimethylsilyl)oxy)propanoate (**5l**), ^{1}H NMR spectrum

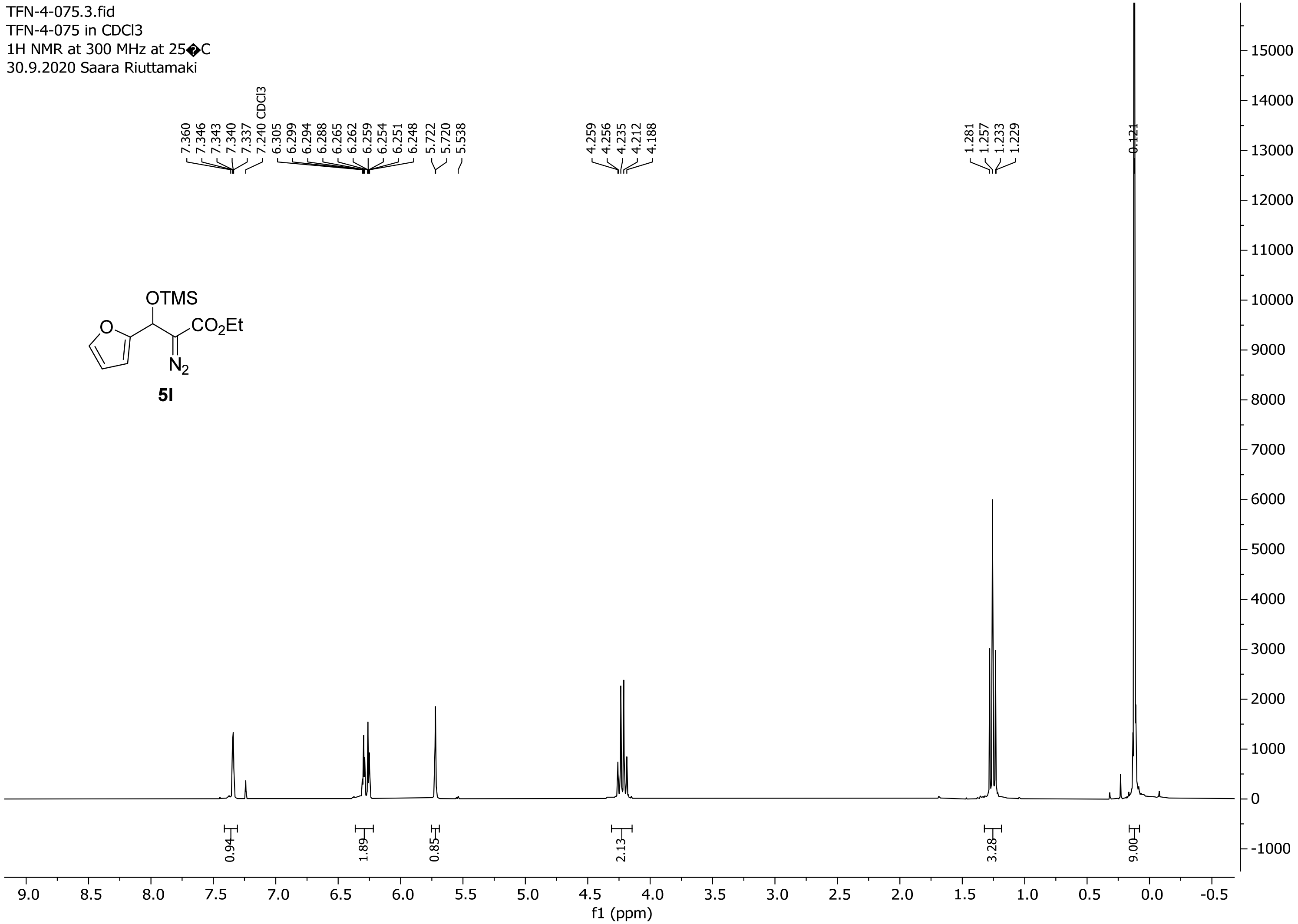

2.1.24 Ethyl 2-diazo-3-(furan-2-yl)-3-((trimethylsilyl)oxy)propanoate (**5l**), $^{13}C\{^1H\}$ NMR spectrum

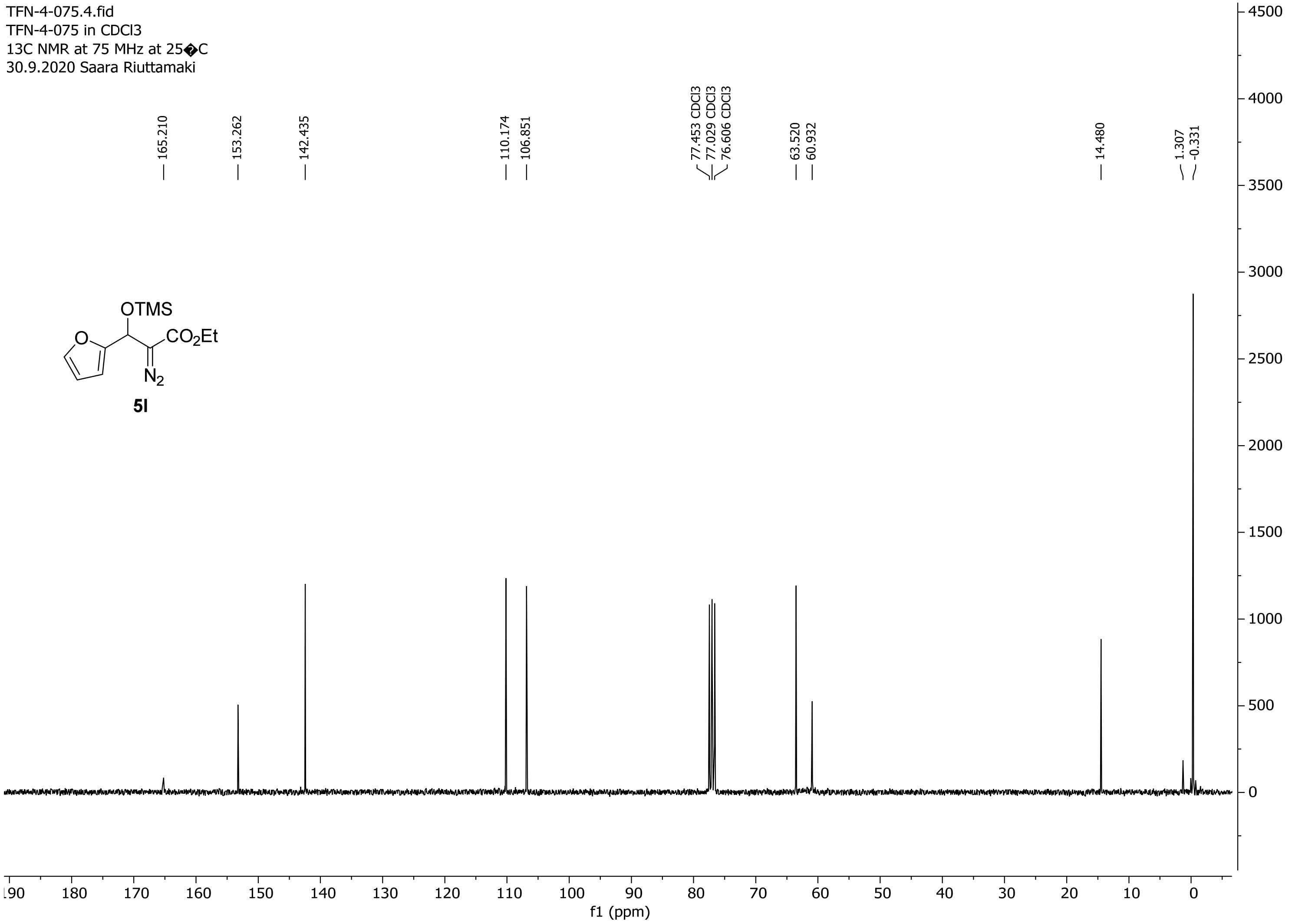

2.1.25 *Ethyl 2-diazo-3-((trimethylsilyl)oxy)-3-(5-(((trimethylsilyl)oxy)methyl)furan-2-yl)propanoate* (**5m**), ^{1}H NMR spectrum

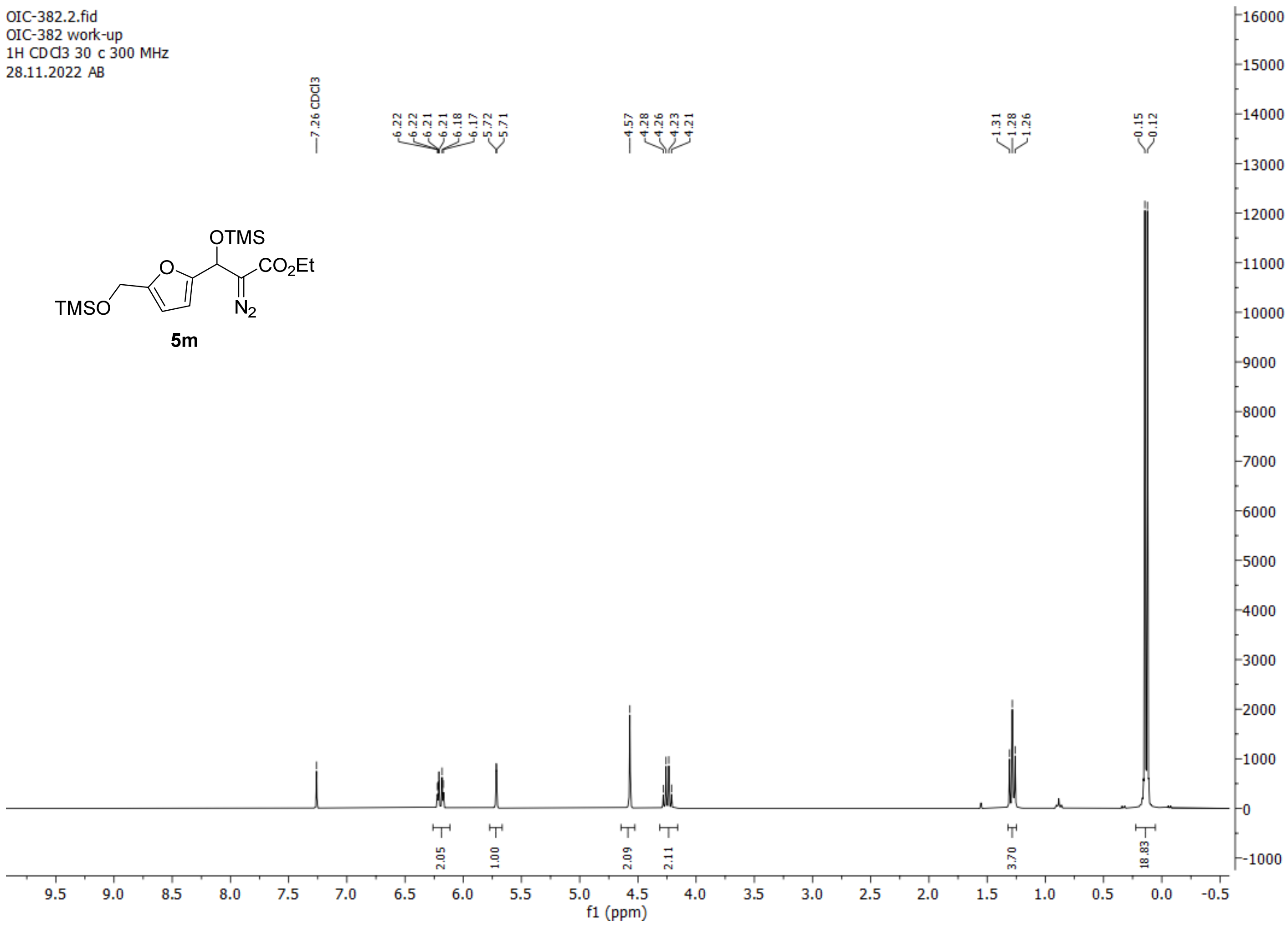

2.1.26 *Ethyl 2-diazo-3-((trimethylsilyl)oxy)-3-(5-(((trimethylsilyl)oxy)methyl)furan-2-yl)propanoate* (**5m**), $^{13}C\{^{1}H\}$ NMR spectrum

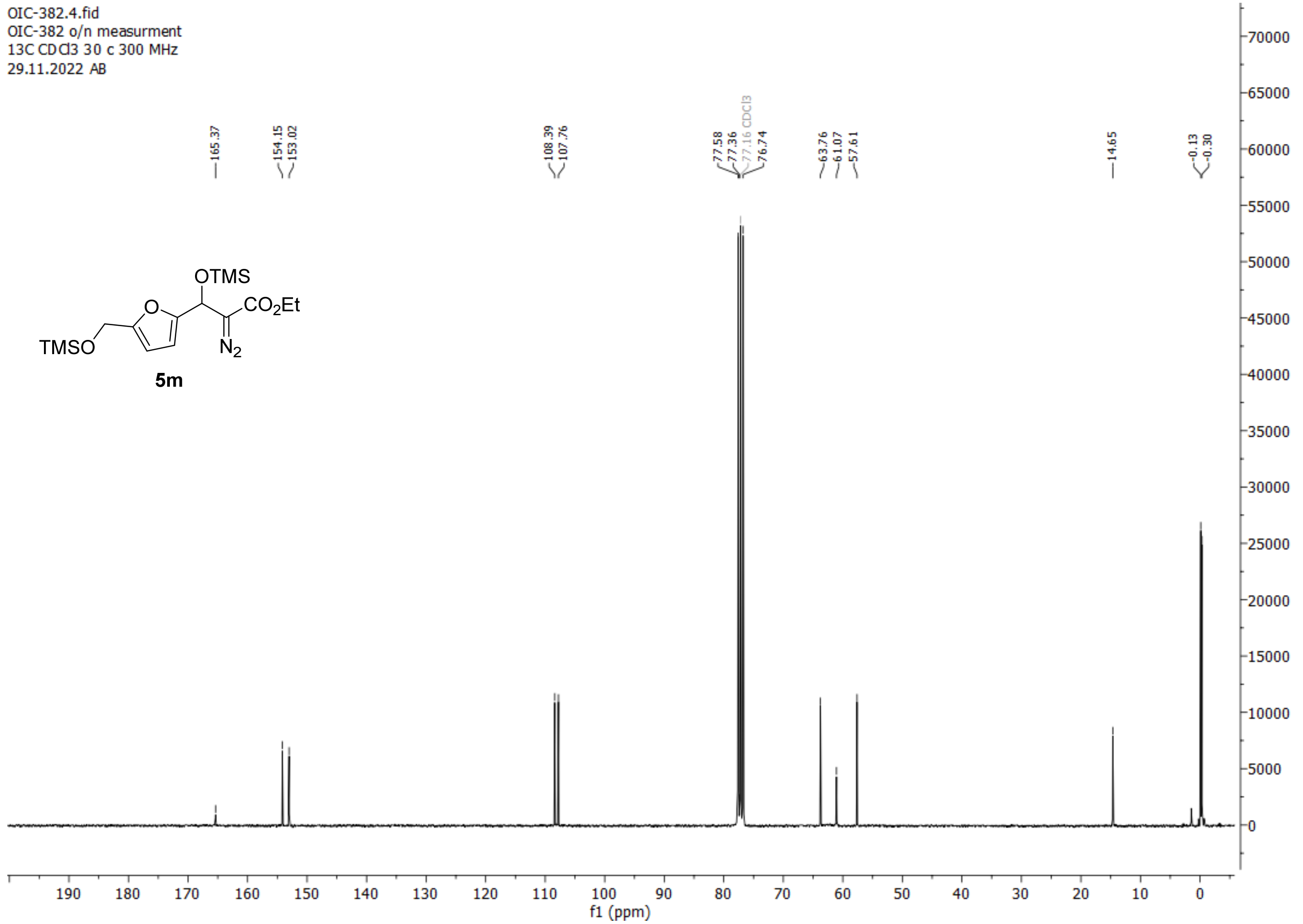

2.1.27 *tert*-Butyl (*R*)-3-((*S*)-2-diazo-3-ethoxy-3-oxo-1-((trimethylsilyl)oxy)propyl)-1-oxa-4-azaspiro[4.5]decane-4-carboxylate (**5n**), ^{1}H NMR spectrum

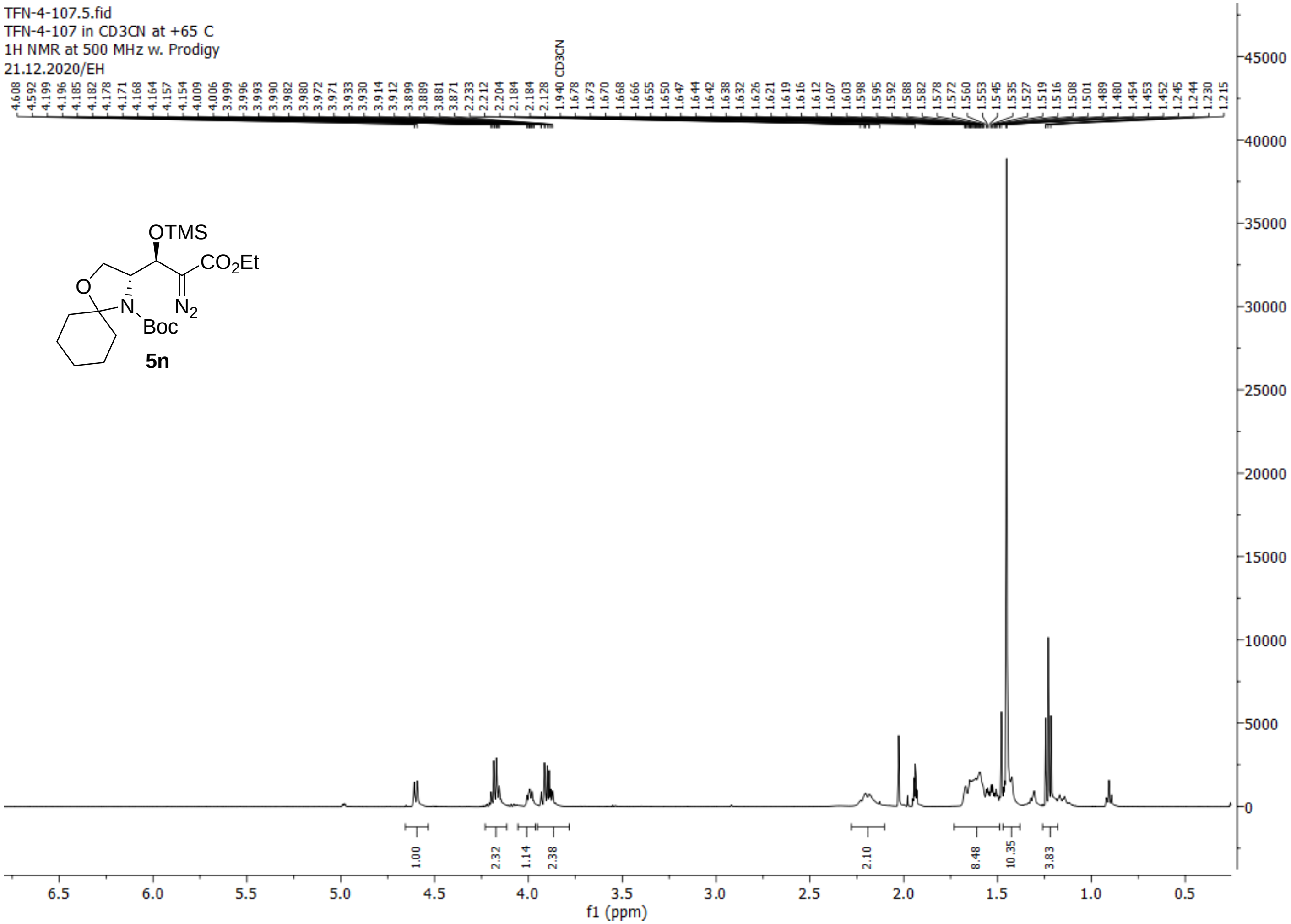

2.1.28 *tert*-Butyl (*R*)-3-((*S*)-2-diazo-3-ethoxy-3-oxo-1-((trimethylsilyl)oxy)propyl)-1-oxa-4-azaspiro[4.5]decane-4-carboxylate (**5n**), $^{13}C\{^{1}H\}$ NMR spectrum

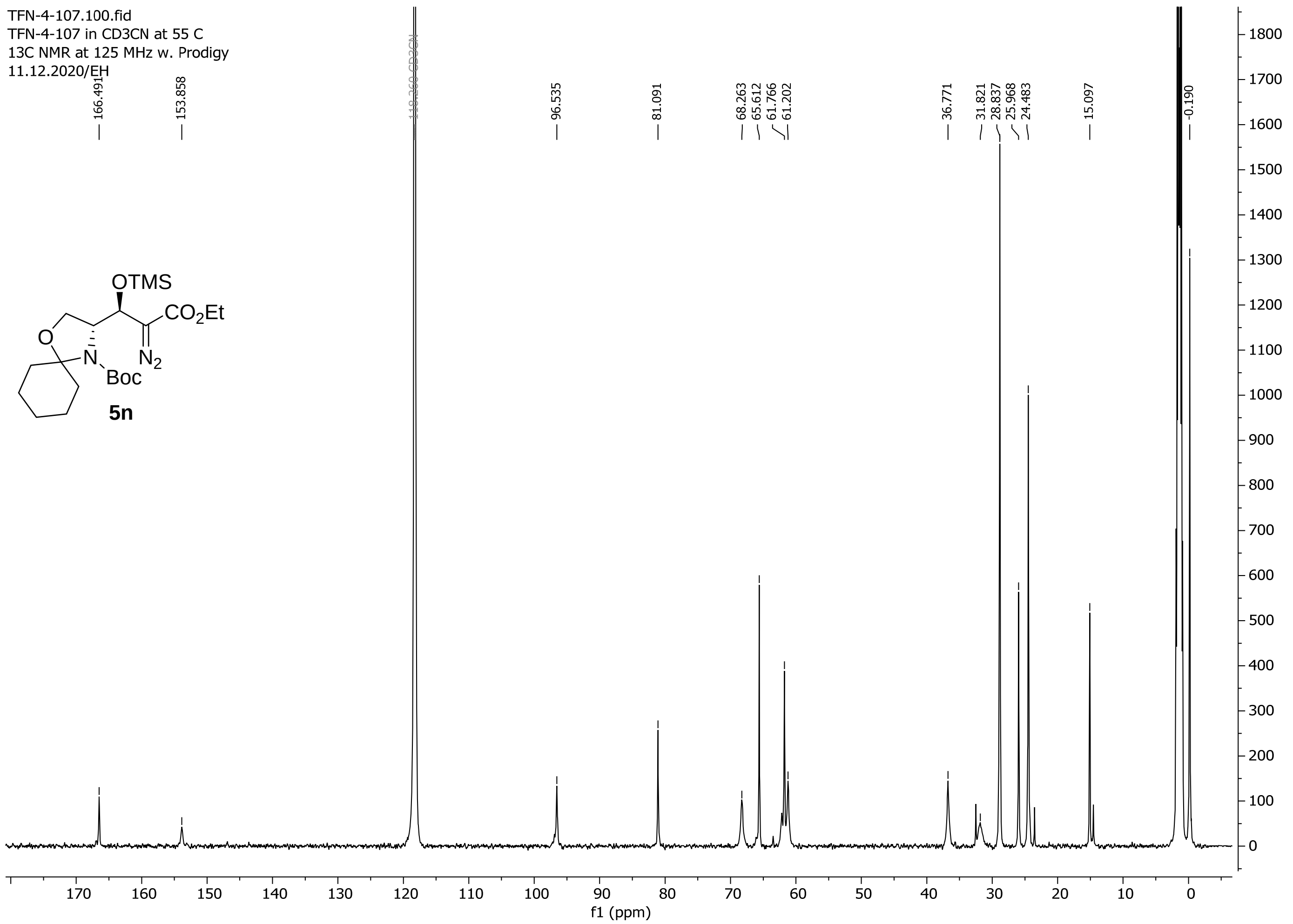

2.1.29 *tert*-Butyl (*R*)-3-((*R*)-2-diazo-3-ethoxy-1-hydroxy-3-oxopropyl)-1-oxa-4-azaspiro[4.5]decane-4-carboxylate (**6**), ^{1}H NMR spectrum

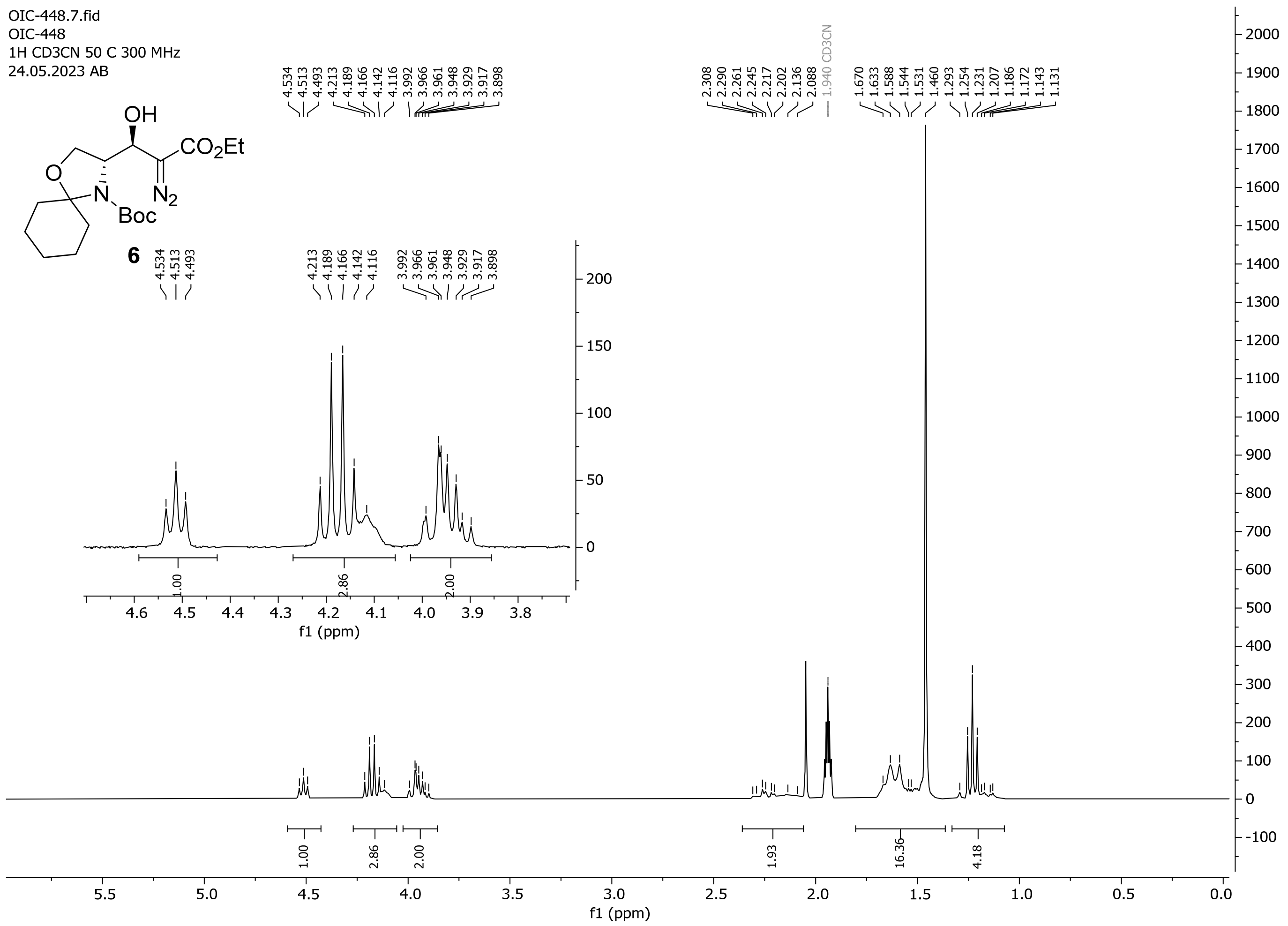

2.1.30 *tert*-Butyl (*R*)-3-((*R*)-2-diazo-3-ethoxy-1-hydroxy-3-oxopropyl)-1-oxa-4-azaspiro[4.5]decane-4-carboxylate (**6**), $^{13}C\{^{1}H\}$ NMR spectrum

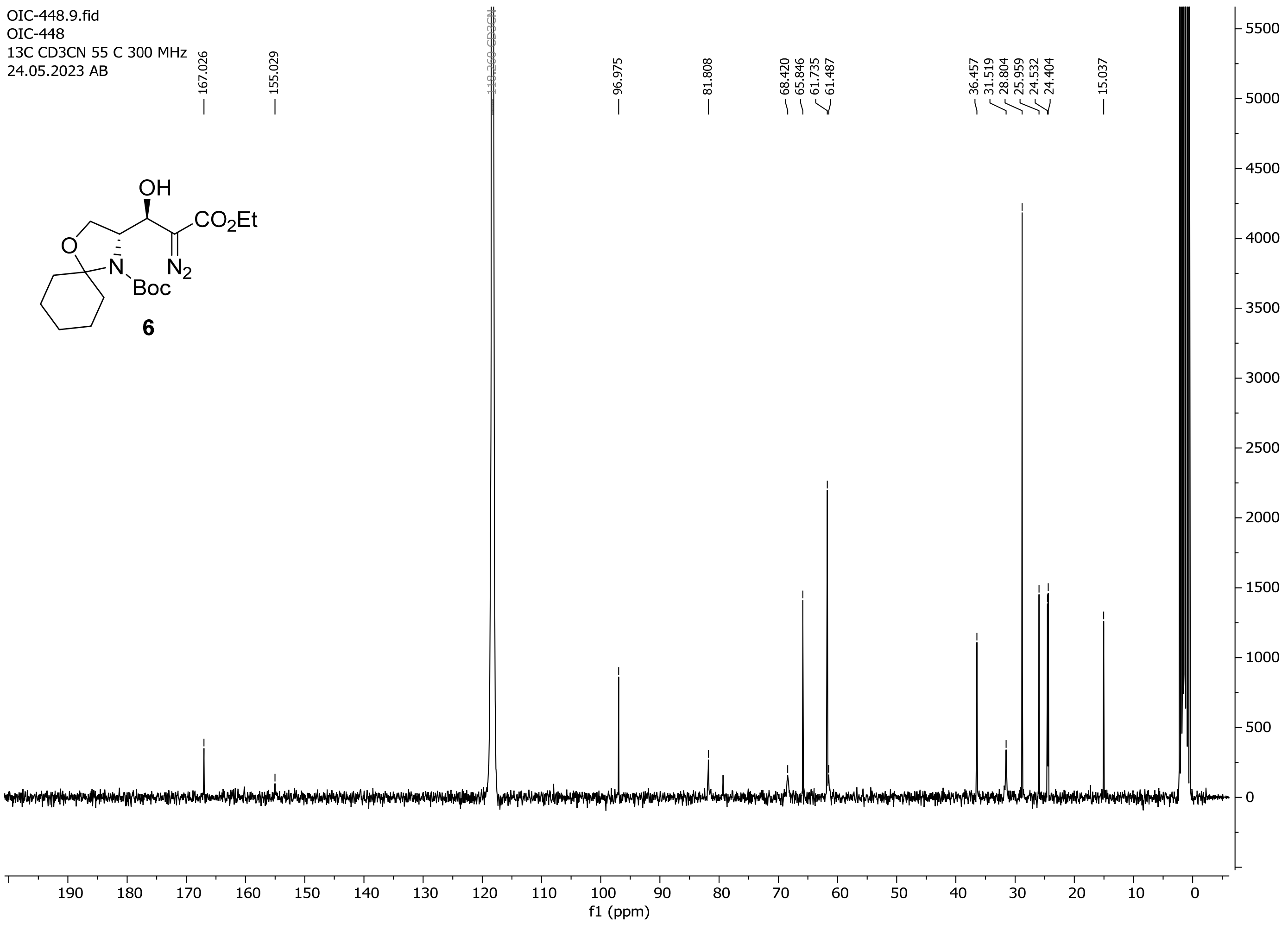

2.1.31 *tert*-Butyl (*R*)-3-((*S*)-3-ethoxy-3-oxo-1-((trimethylsilyl)oxy)propyl)-1-oxa-4-azaspiro[4.5]decane-4-carboxylate (**7**), ^{1}H NMR spectrum

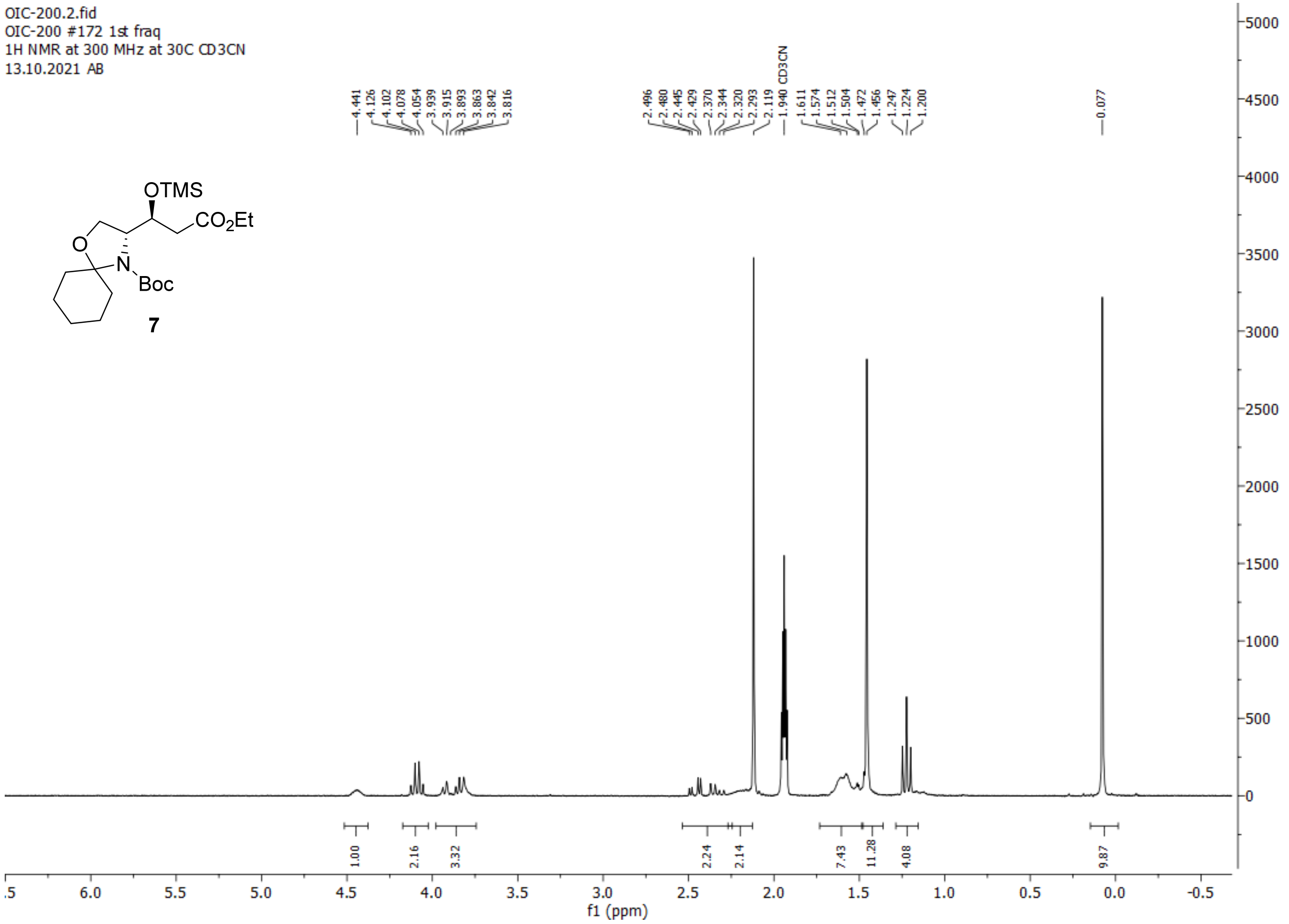

2.1.32 *tert*-Butyl (*R*)-3-((*S*)-3-ethoxy-3-oxo-1-((trimethylsilyl)oxy)propyl)-1-oxa-4-azaspiro[4.5]decane-4-carboxylate (**7**), $^{13}C\{^{1}H\}$ NMR spectrum

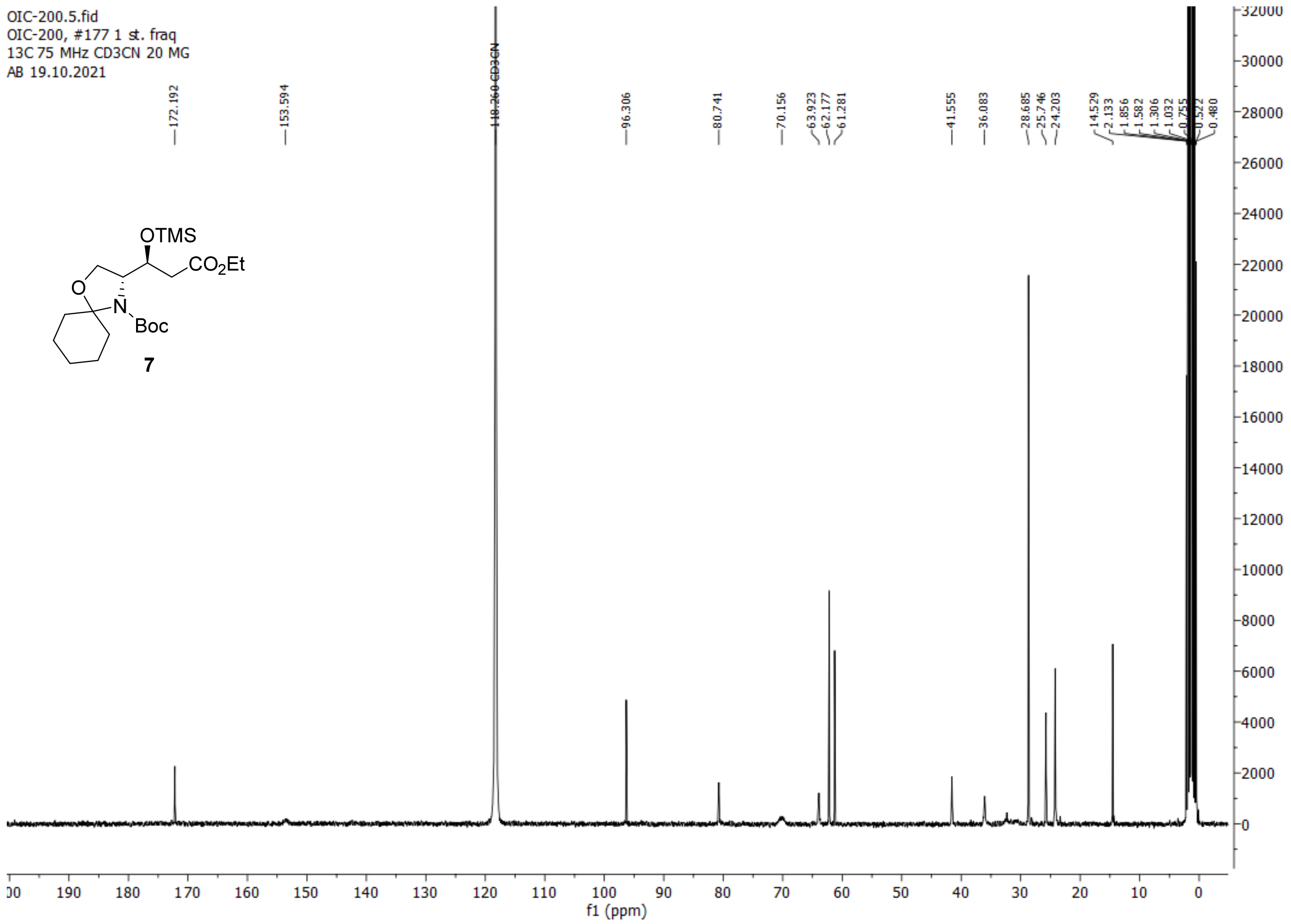

2.1.33 *tert*-Butyl (*R*)-3-((*S*)-3-ethoxy-1-hydroxy-3-oxopropyl)-1-oxa-4-azaspiro[4.5]decane-4-carboxylate (**8**), ^{1}H NMR spectrum

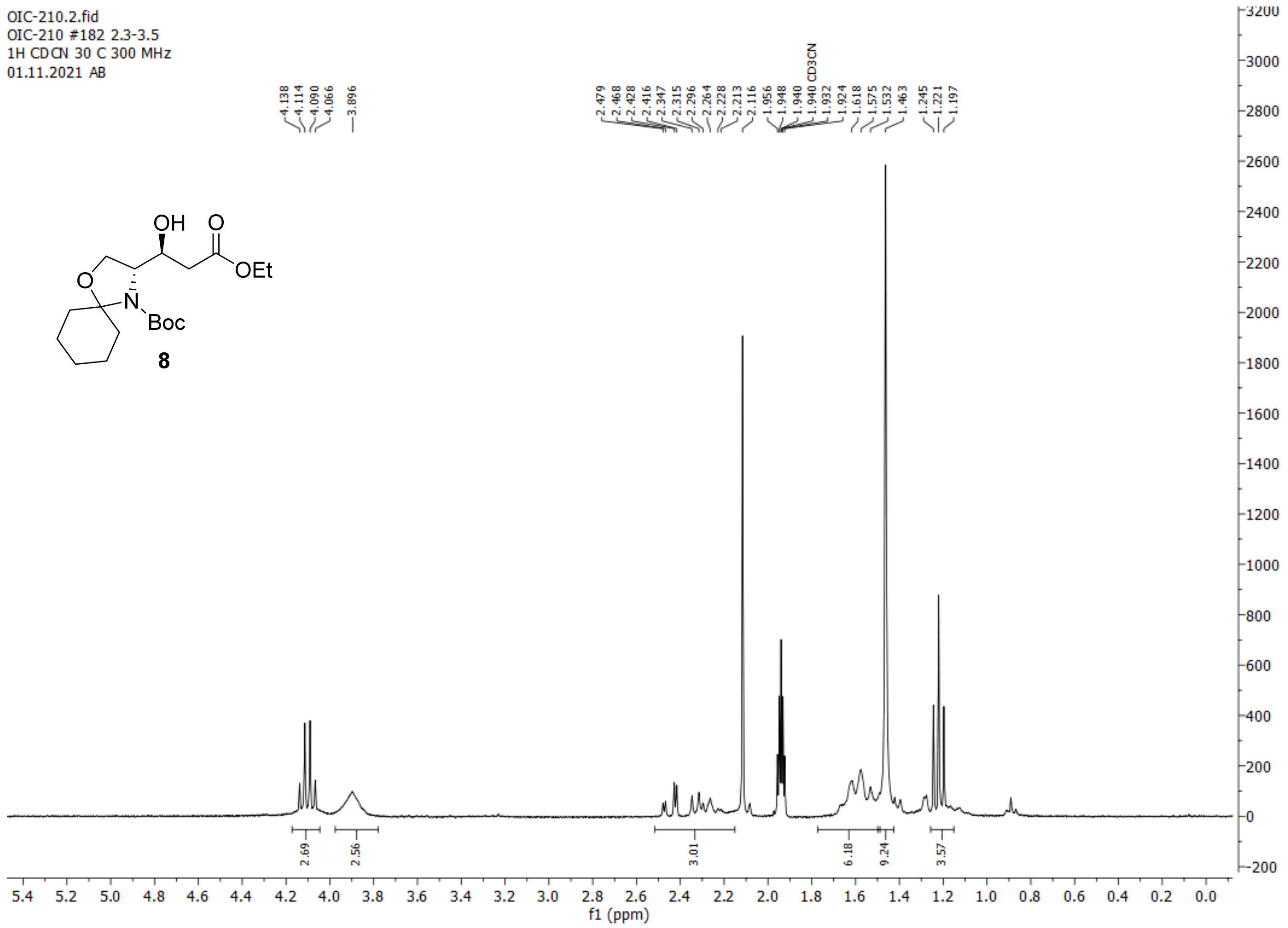

2.1.34 *tert*-Butyl (*R*)-3-((*S*)-3-ethoxy-1-hydroxy-3-oxopropyl)-1-oxa-4-azaspiro[4.5]decane-4-carboxylate (**8**), $^{13}C\{^1H\}$ NMR spectrum

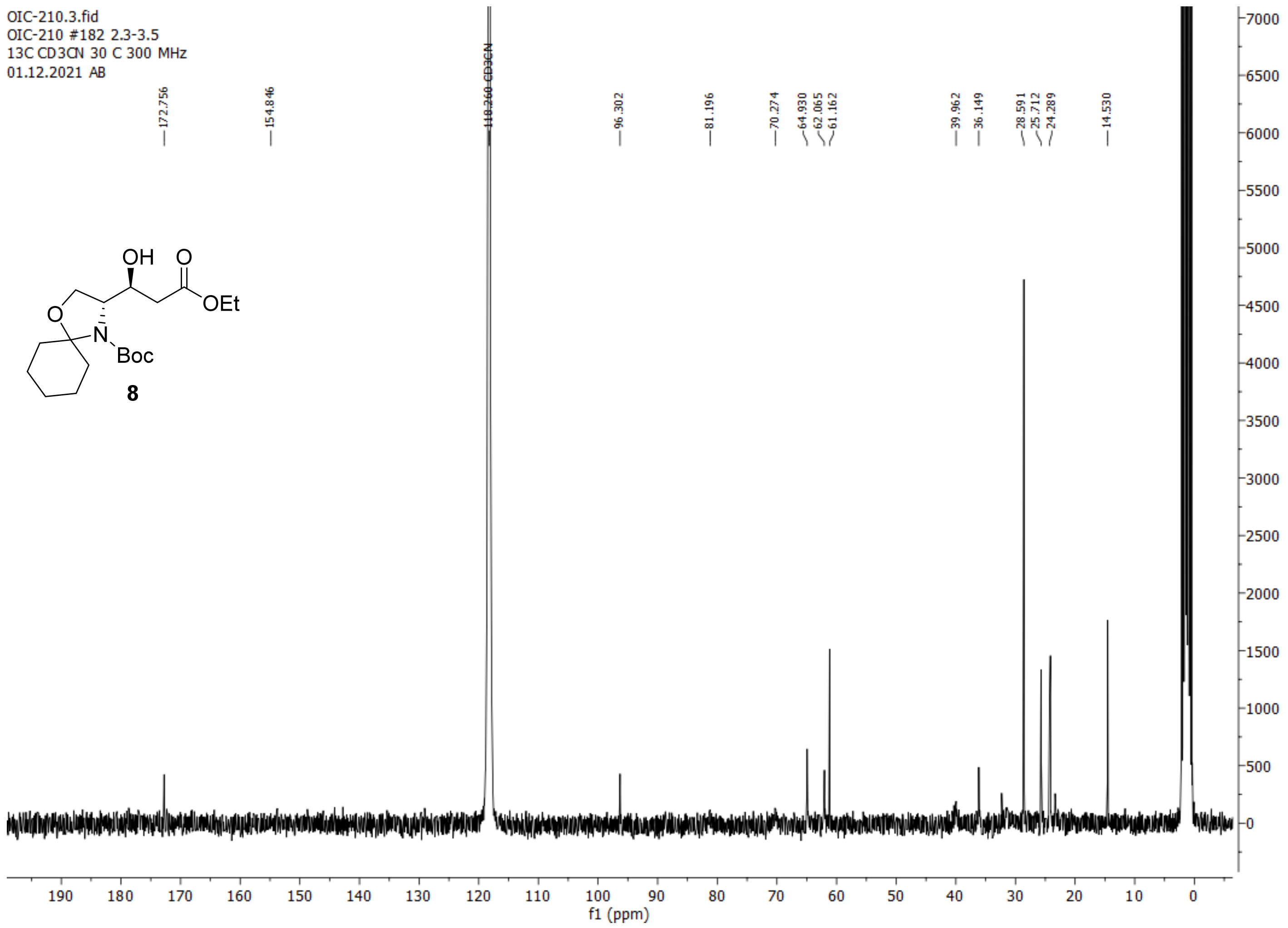

2.1.35 Ethyl 2-((*4S,5R*)-5-((*tert*-butoxycarbonyl)amino)-2,2-dimethyl-1,3-dioxan-4-yl)acetate (**9a**), ^{1}H NMR spectrum

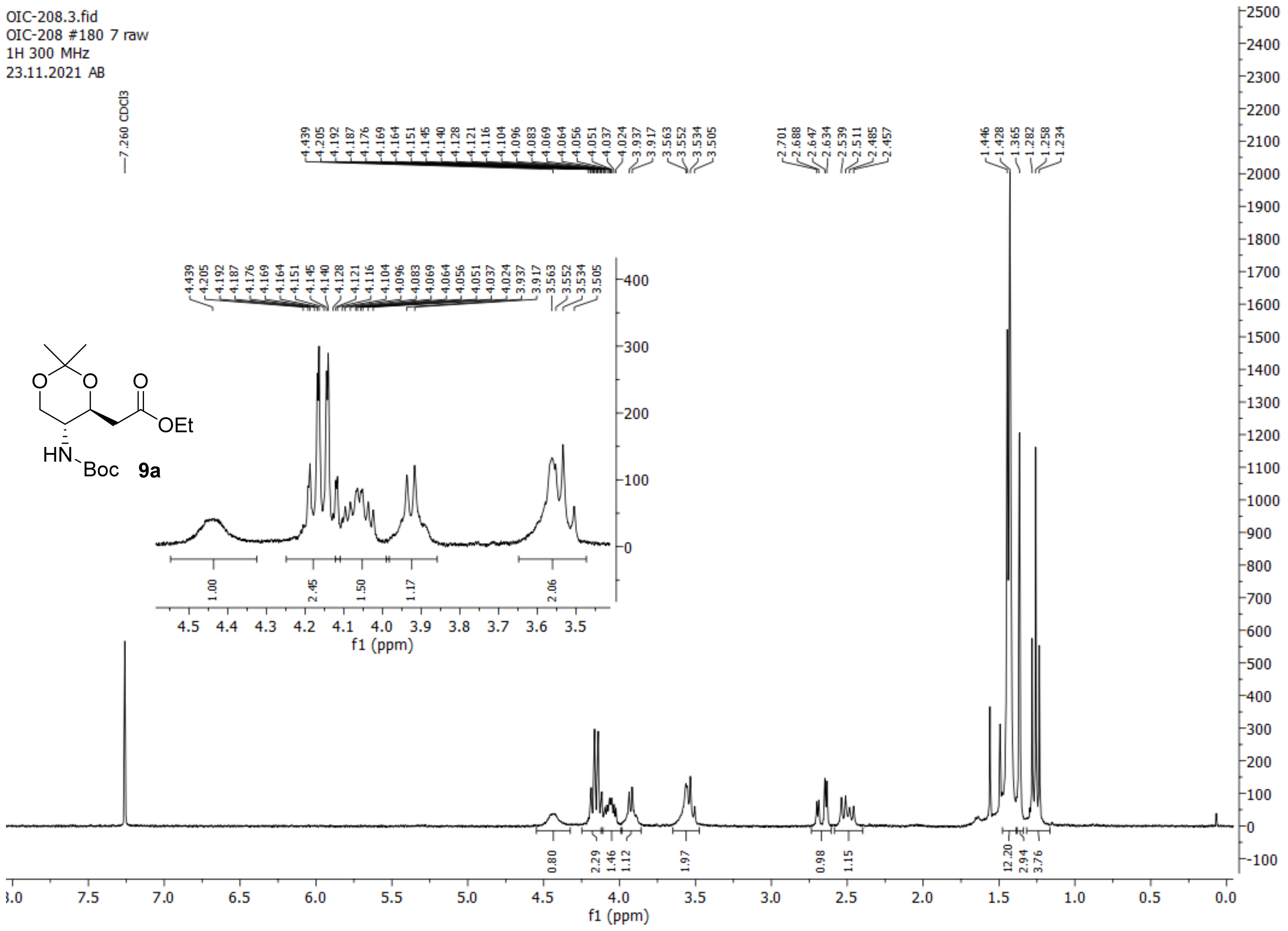

2.1.36 Ethyl 2-((*4S,5R*)-5-((*tert*-butoxycarbonyl)amino)-2,2-dimethyl-1,3-dioxan-4-yl)acetate (**9a**), $^{13}C\{^1H\}$ NMR spectrum

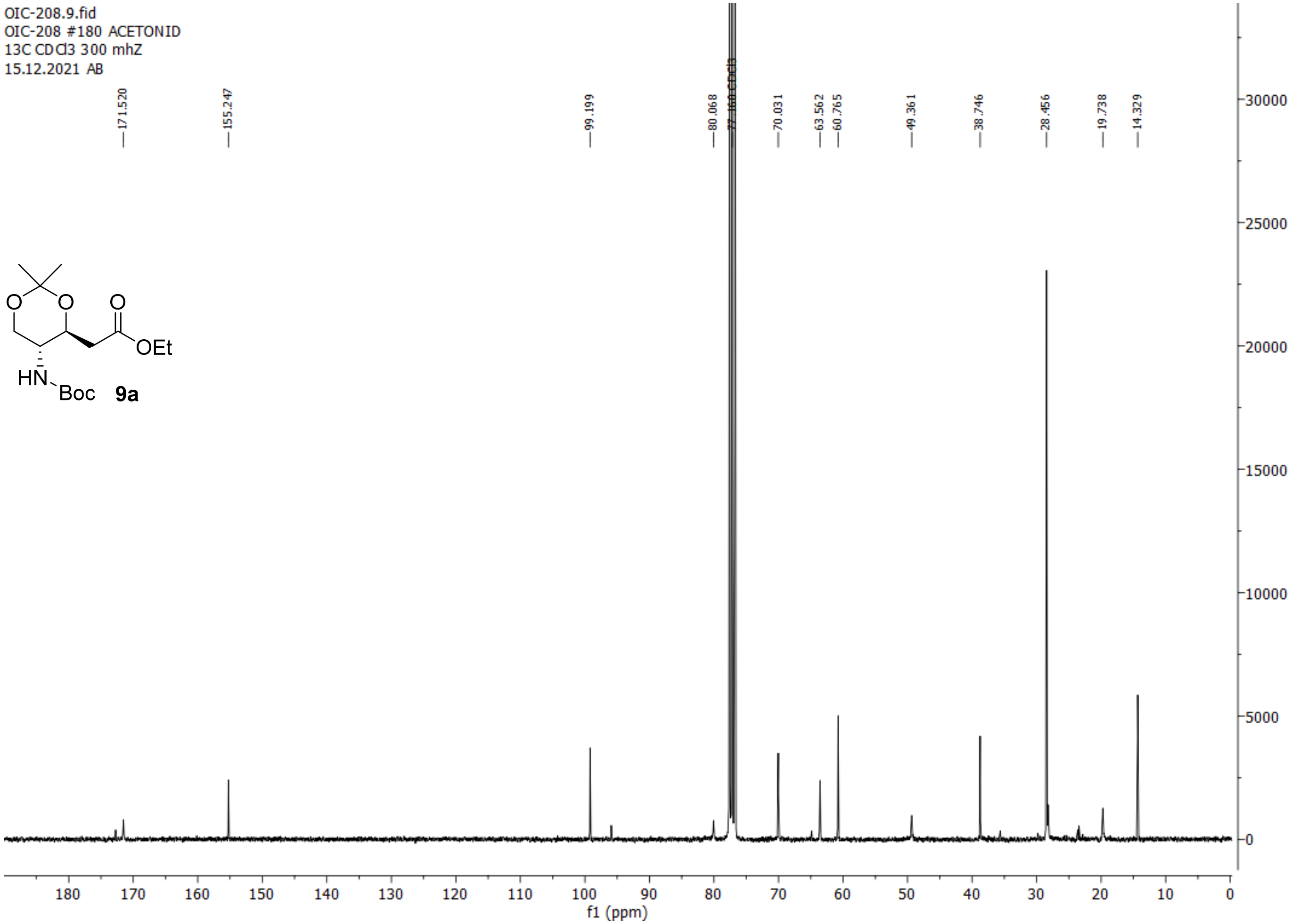

2.1.37 Ethyl 2-((*2S,3R*)-3-((*tert*-butoxycarbonyl)amino)-1,5-dioxaspiro[5.5]undecan-2-yl)acetate (**9b**), ^{1}H NMR spectrum

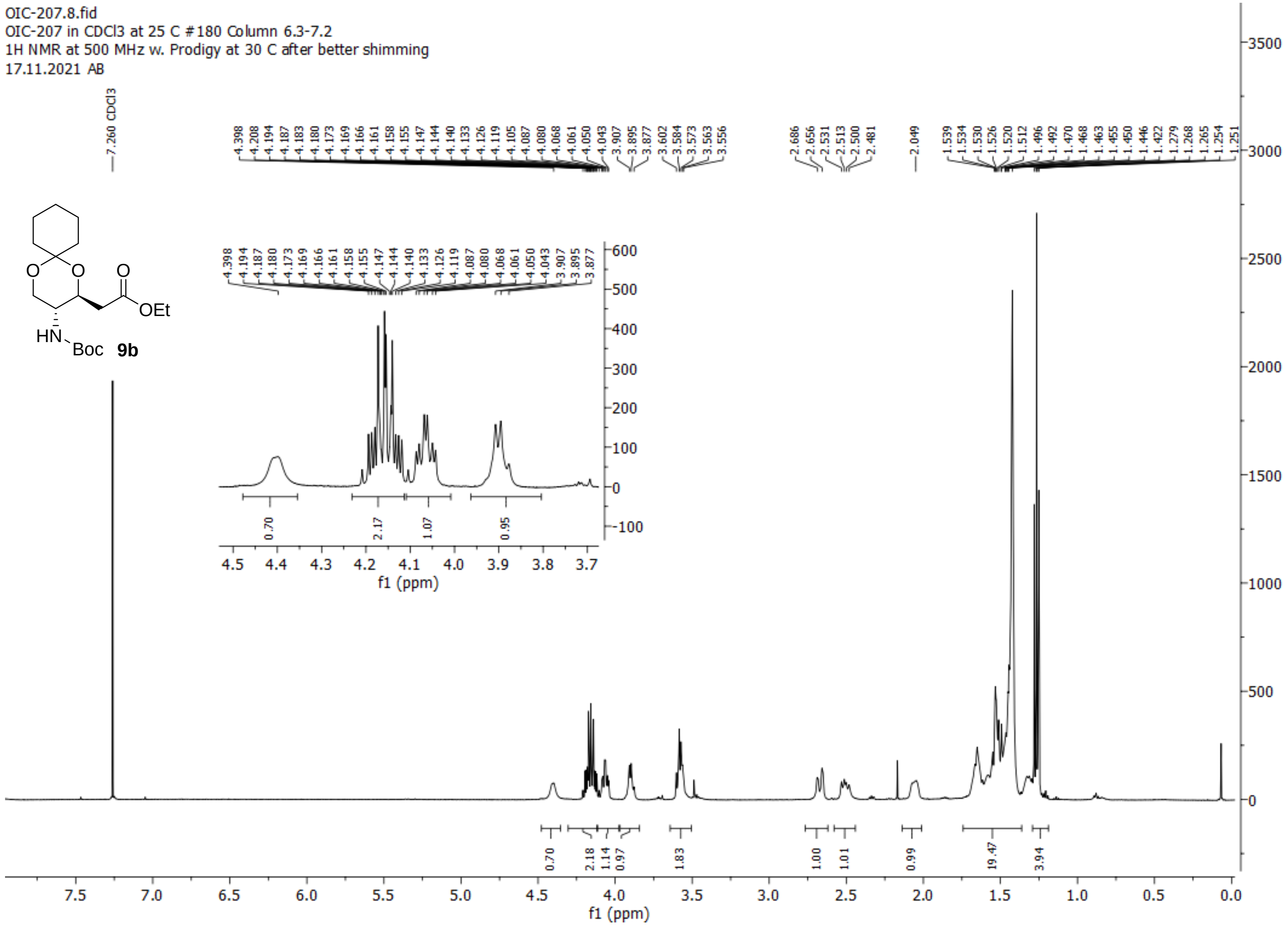

2.1.38 Ethyl 2-((*2S,3R*)-3-((*tert*-butoxycarbonyl)amino)-1,5-dioxaspiro[5.5]undecan-2-yl)acetate (**9b**), $^{13}C\{^{1}H\}$ NMR spectrum

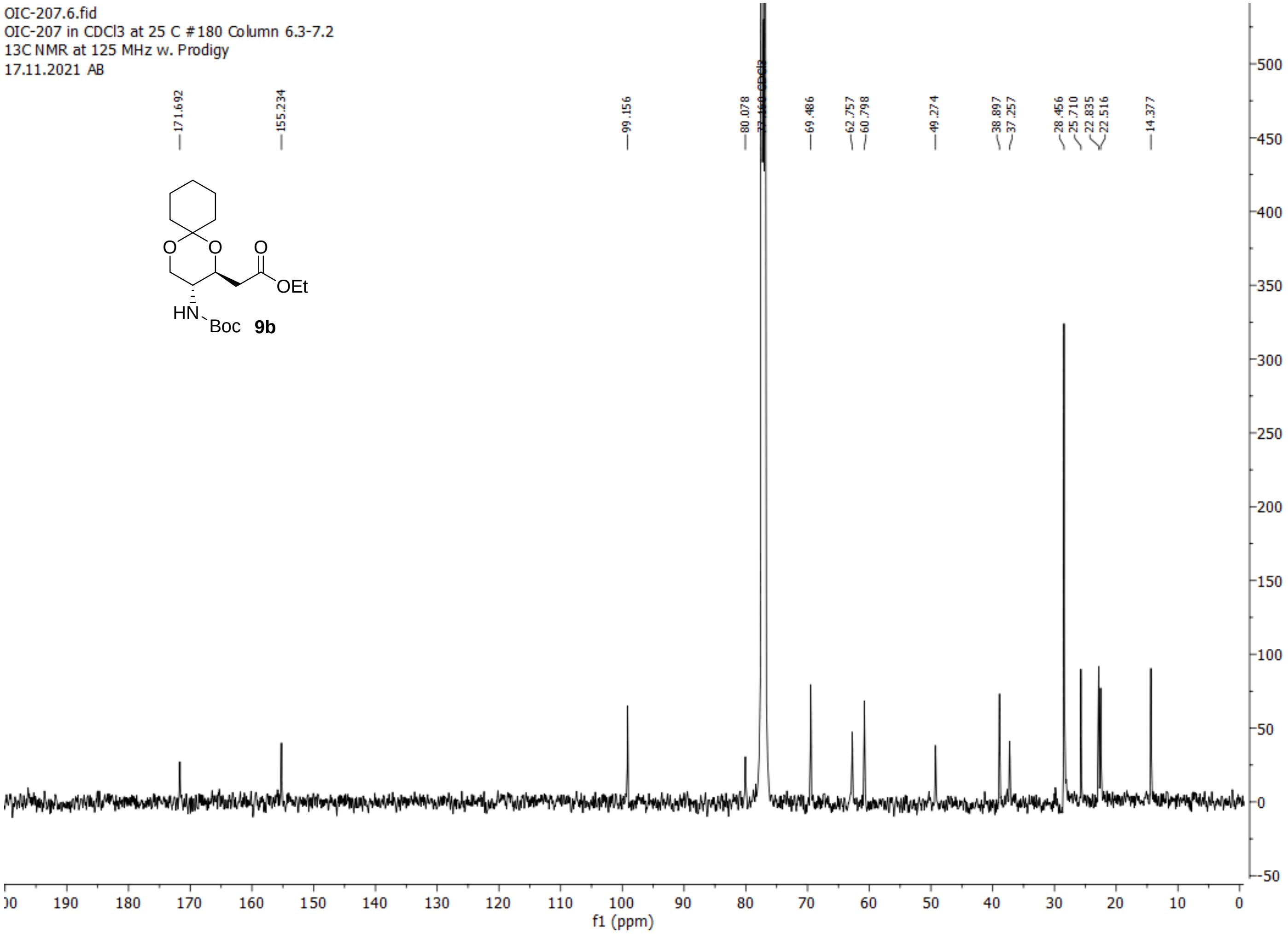

2.1.39 *tert*-Butyl 3-(2-diazo-3-ethoxy-3-oxo-1-((trimethylsilyl)oxy)propyl)-1-oxa-4-azaspiro[4.5]decane-4-carboxylate (***(±)*-5n**), ^{1}H NMR spectrum

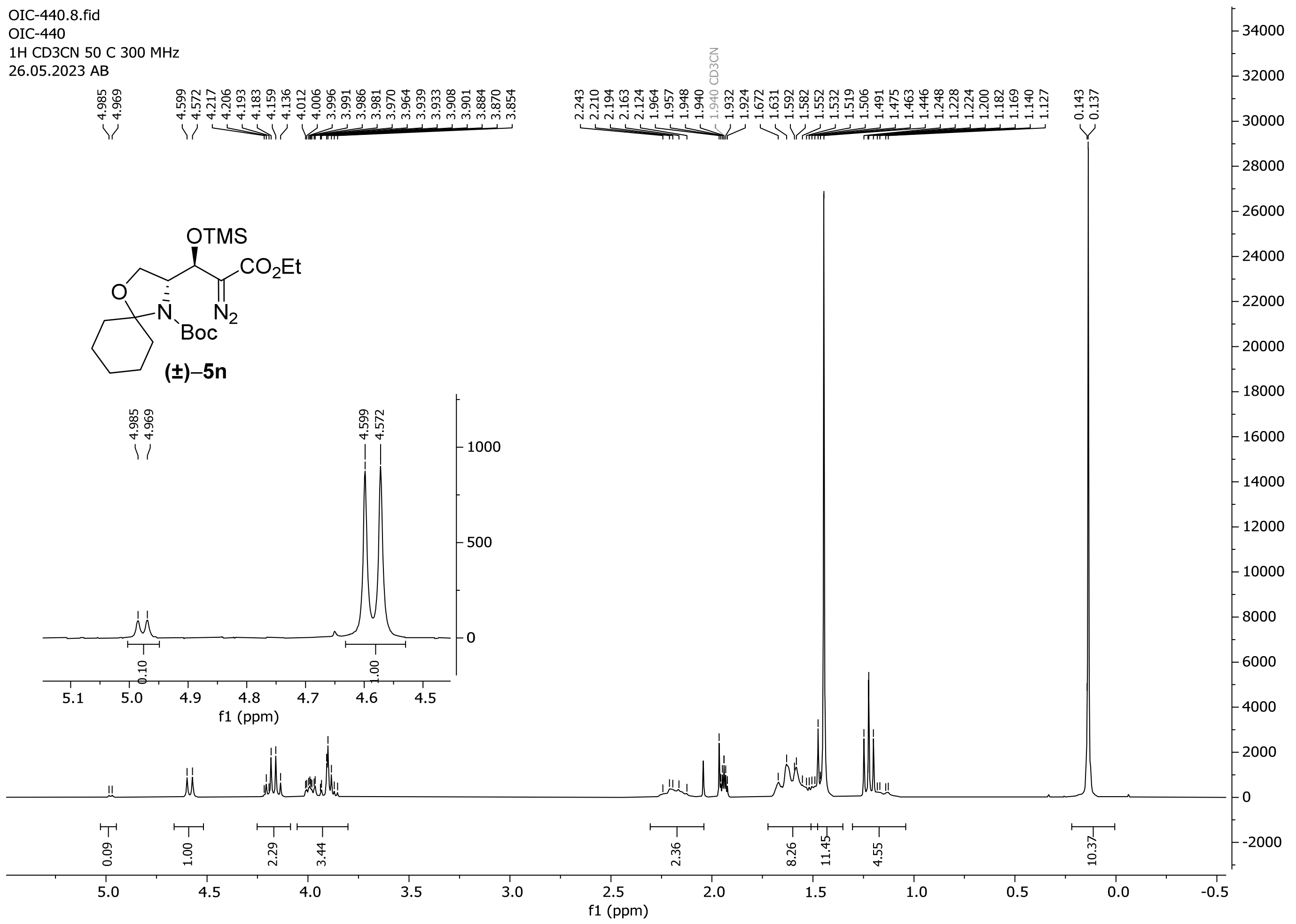

2.1.40 *tert*-Butyl 3-(2-diazo-3-ethoxy-3-oxo-1-((trimethylsilyl)oxy)propyl)-1-oxa-4-azaspiro[4.5]decane-4-carboxylate (***(±)*-5n**), $^{13}C\{^{1}H\}$ NMR spectrum

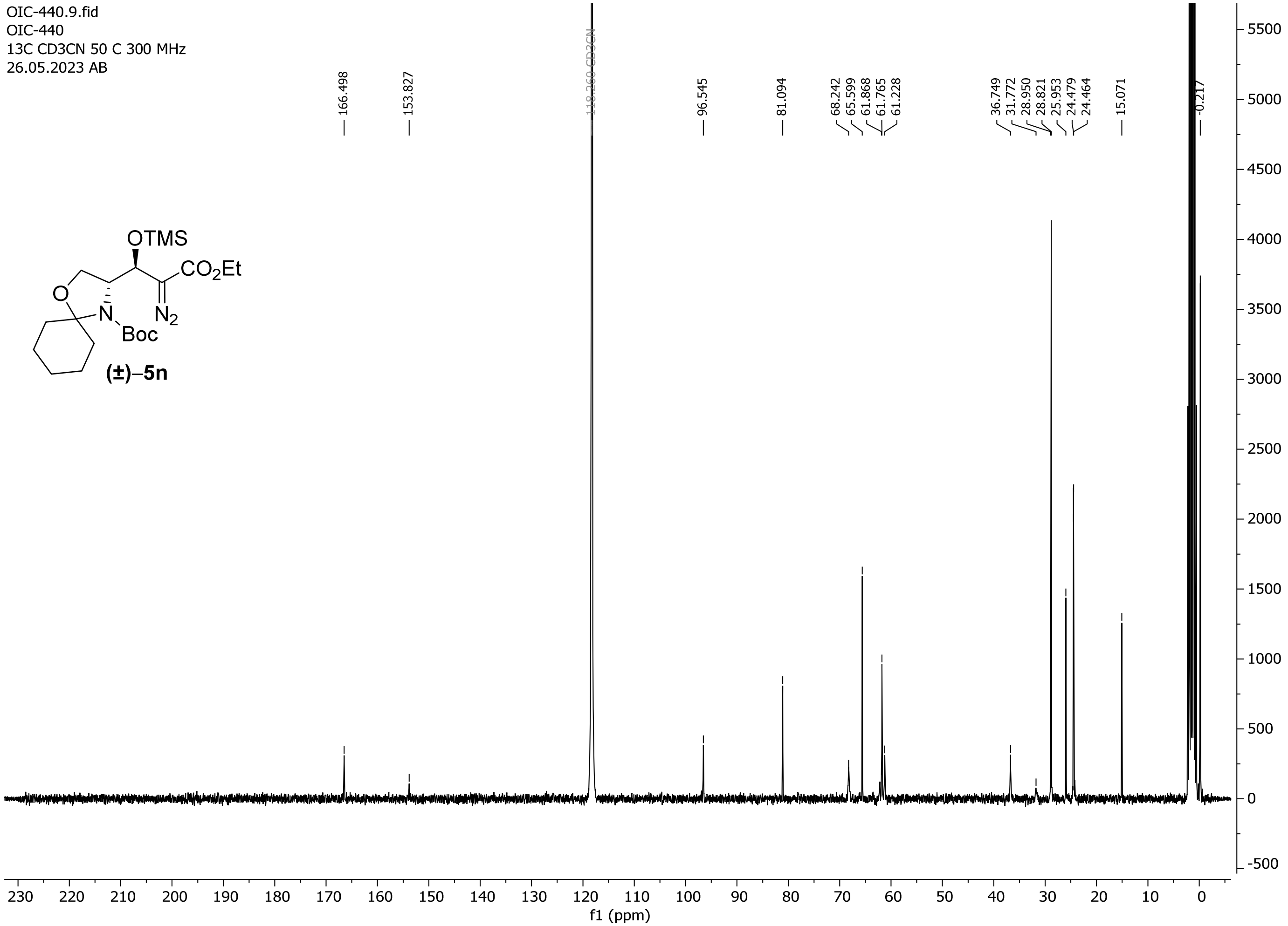

2.1.41 *tert*-Butyl 3-(2-diazo-3-ethoxy-1-hydroxy-3-oxopropyl)-1-oxa-4-azaspiro[4.5]decane-4-carboxylate (***(±)*-6**), ^{1}H NMR spectrum

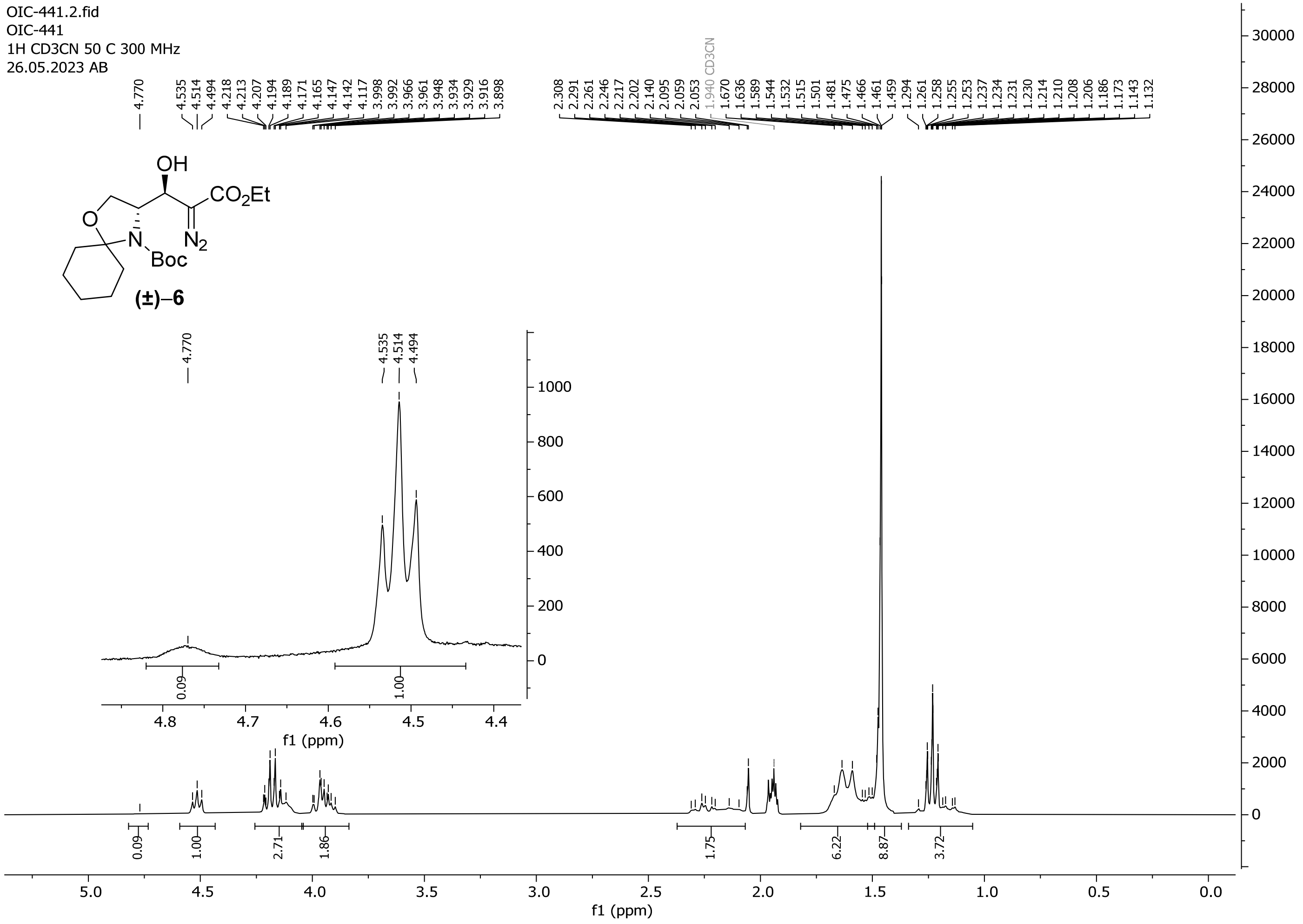

2.1.42 *tert*-Butyl 3-(2-diazo-3-ethoxy-1-hydroxy-3-oxopropyl)-1-oxa-4-azaspiro[4.5]decane-4-carboxylate (***(±)*-6**), $^{13}C\{^{1}H\}$ NMR spectrum

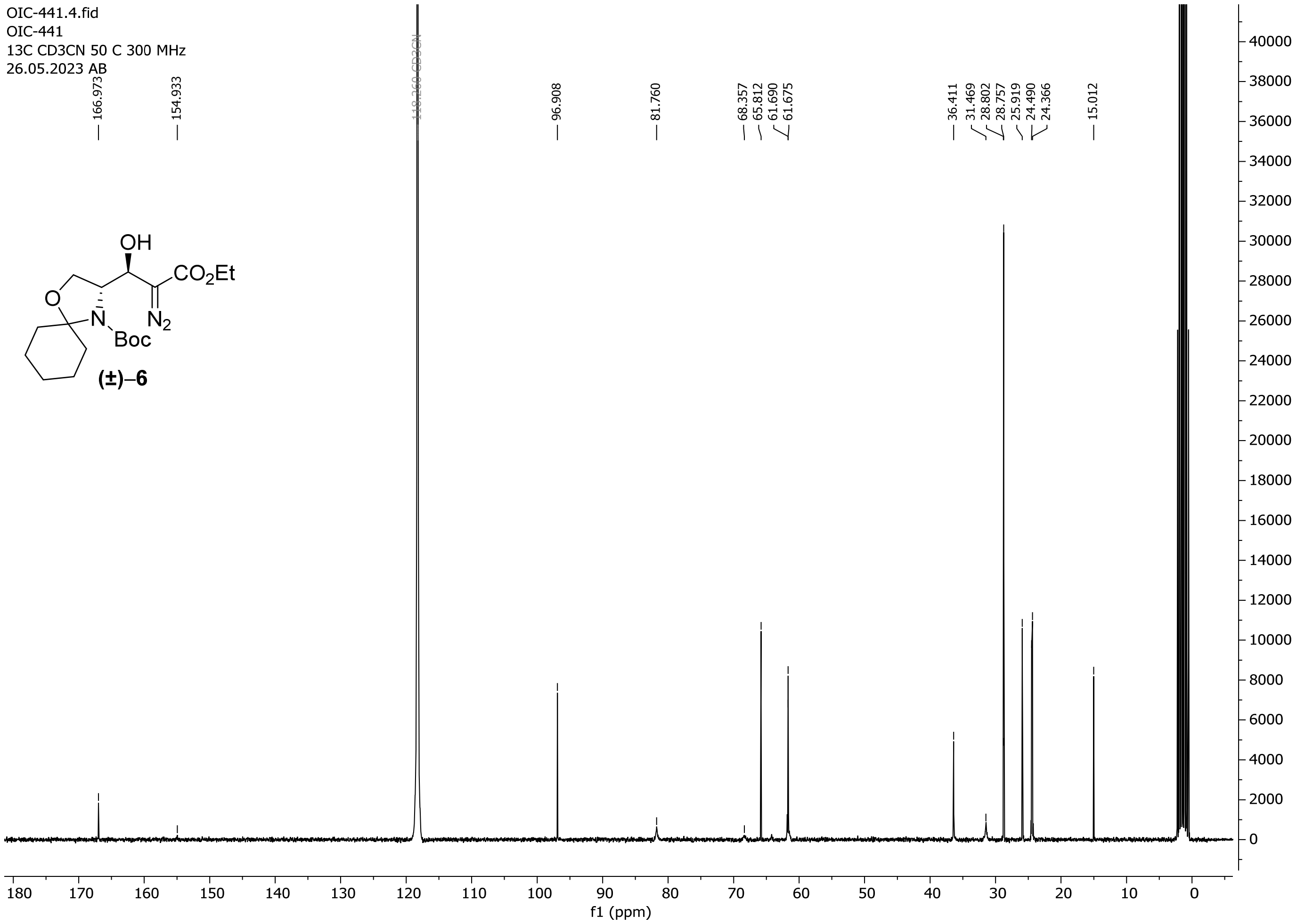

2.2 HPLC measurements

2.2.1 HPLC chromatogram **(±)-6**, racemic mixture from a reaction carried out at RT (internal code OIC-441)

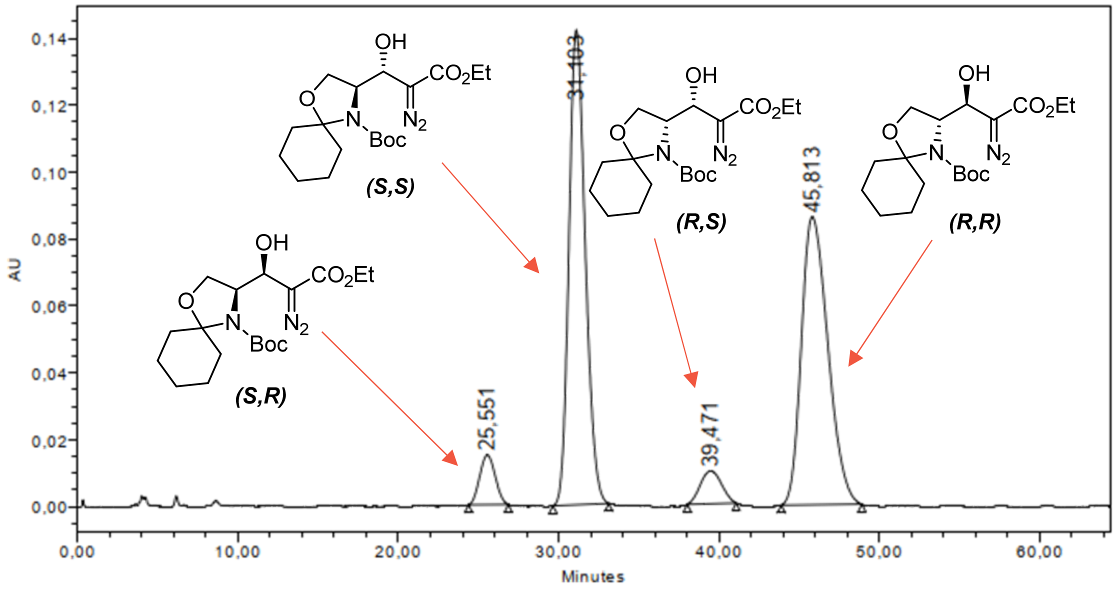

	RT	Area	% Area	Height
1	25,551	995913	4,56	14831
2	31,103	10040212	46,00	141975
3	39,471	903087	4,14	9833
4	45,813	9886390	45,30	86112

2.2.2 HPLC chromatogram (***R,R***)-**6** from a reaction carried out at 0 ^{0}C (internal code OIC-448)

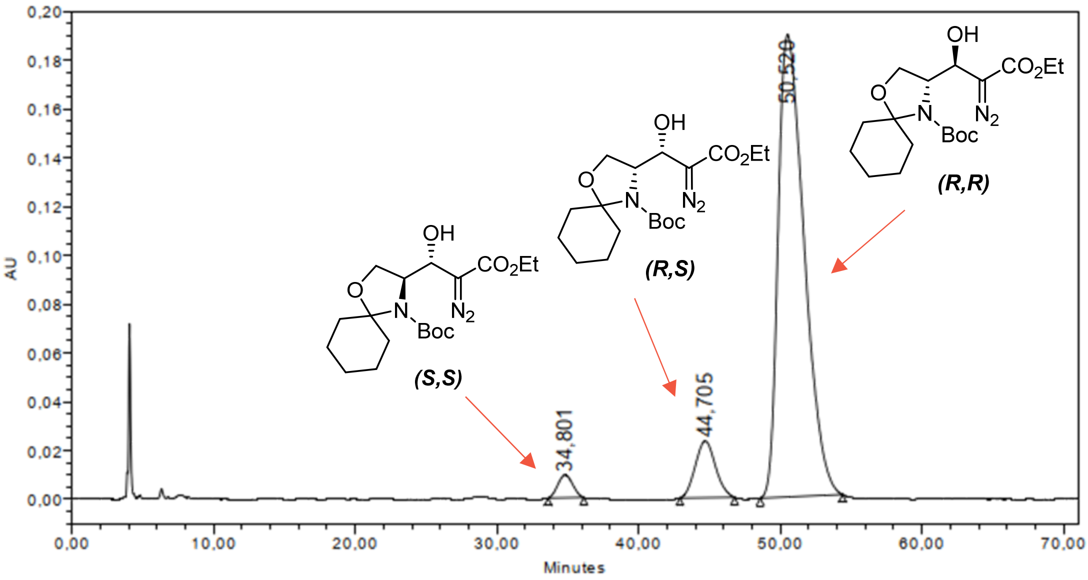

	RT	Area	% Area	Height
1	34,801	674720	2,48	9305
2	44,705	2308920	8,50	23238
3	50,520	24180326	89,02	189448

2.2.3 HPLC chromatogram (***R,R***)-**6** from a reaction carried out at RT (internal code OIC-447)

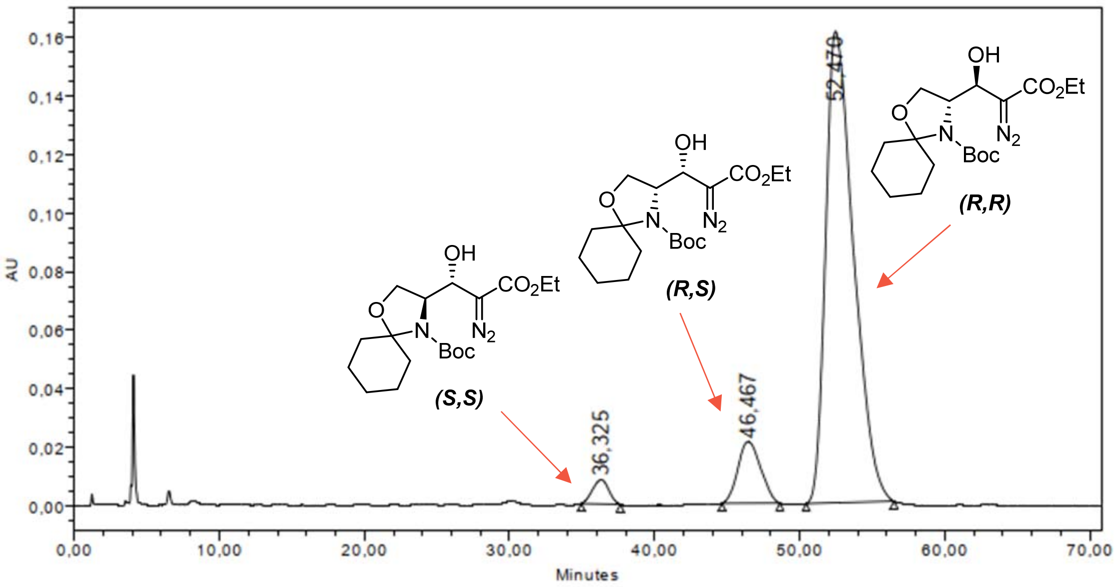

	RT	Area	% Area	Height
1	36,325	645644	2,64	8274
2	46,467	2295571	9,39	21074
3	52,470	21498900	87,97	161152

2.3 ^{1}H NMR control experiments

2.3.1 Control experiment without TMAP (internal code OIC-443)

Procedure: Benzaldehyde (1.0 equiv, 37 μmol), ethyl diazoacetate (1.1 equiv., 41 μmol), *N,O*-bis(trimethylsilyl)acetamide (2.0 equiv., 75 μmol), dibenzyl ether (1 equiv., 37 μmol) were dissolved in CD_3CN (600 μL). The resulting yellow solution was transferred to an NMR tube, was capped and inverted three times to ensure homogeneity, and the sample was inserted into the NMR probe to record the spectra. The reaction was followed by ^{1}H NMR for 3 days by monitoring the diagnostic benzaldehyde ^{1}H NMR peak at 10.01 ppm. Between measurements NMR tube has been kept at r.t. in the absence of light. The results were processed with MestReNova Single Rection Monitoring plug-in and converted into concentrations using integration of internal standard with the correction on the initial concentration, and the relative rate was obtained by exponential fit using OriginPro 2017 followed the equation $y = 0.027 + 0.037 \times\ e^{(-6.187\times x)}$.

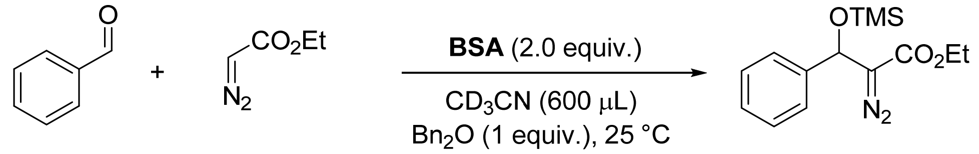

1c (6.6 mg/mL) 1 equiv. **5a**

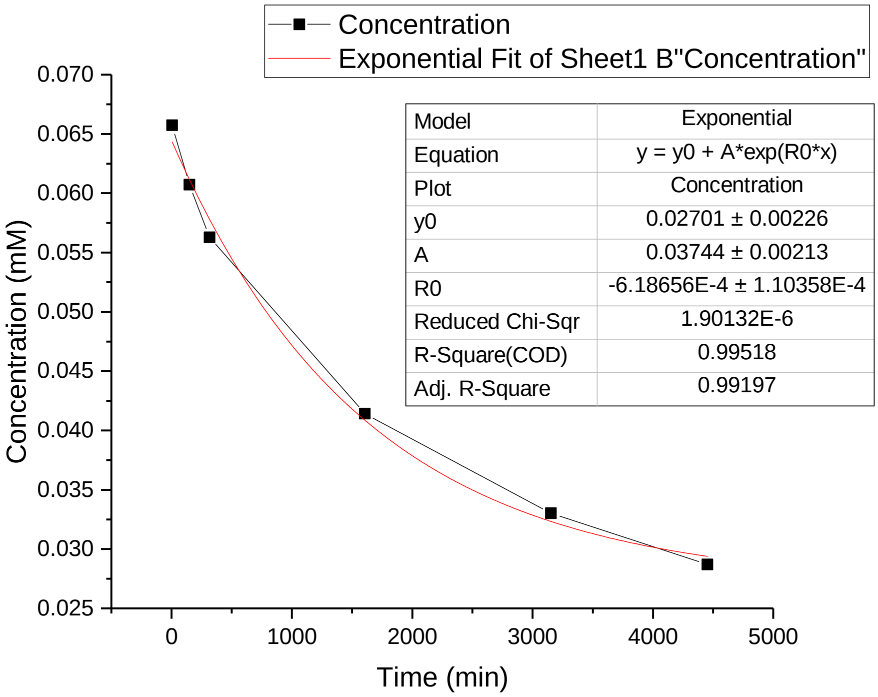

2.3.2 TMS- ethyl diazoacetate formation (internal code OIC-444)

Procedure: Ethyl diazoacetate (1 equiv., 48 μmol), *N*,*O*-bis(trimethylsilyl)acetamide (2.0 equiv., 96 μmol), dibenzyl ether (1 equiv., 48 μmol) were dissolved in CD_3CN (600 μL). The resulting yellow solution was transferred to an NMR tube and catalyst (TMAP solution, 62 mM in CD_3CN, 0.01 equiv.) was then added, the NMR tube was capped and inverted three times to ensure homogeneity, and the sample was reinserted into the NMR probe. The reaction was followed by ^1H NMR for 3 days by monitoring the diagnostic ethyl diazoacetate ^1H NMR peak at 4.93 ppm. Between measurements NMR tube has been kept at r.t. in the absence of light. The results were processed with MestReNova Single Rection Monitoring plug-in and converted into concentrations using integration of internal standard with the correction on the initial concentration, and the relative rate was obtained by exponential fit using OriginPro 2017 followed the equation $y = 0.027 + 0.037 \times e^{(-6.465 \times x)}$.

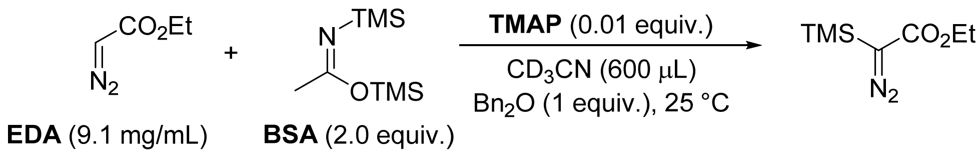

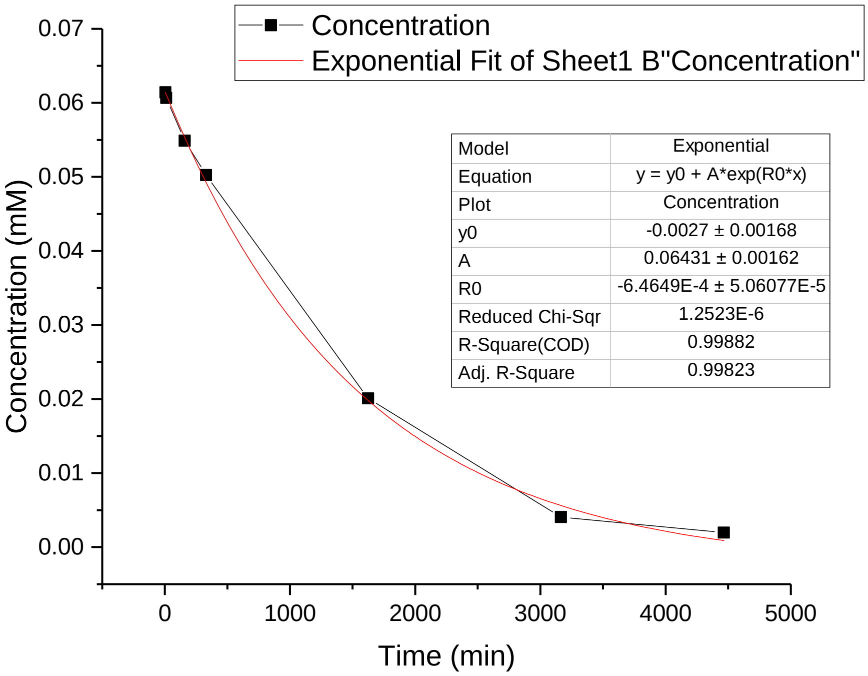

2.3.3 Reaction rate determination with BSA (internal code OIC-451)

Procedure: Benzaldehyde (1.0 equiv, 40 µmol), ethyl diazoacetate (0.9 equiv., 36 µmol), *N,O*-bis(trimethylsilyl)acetamide (2.0 equiv., 80 µmol), dibenzyl ether (1 equiv., 40 µmol) were dissolved in CD_3CN (600 µL). The resulting yellow solution was transferred to an NMR tube and the first ^1H NMR spectrum was recorded. The catalyst (TMAP solution, 62 mM in CD_3CN, 0.001 equiv.) was then added, the NMR tube was capped and inverted three times to ensure homogeneity, and the sample was reinserted into the NMR probe. The reaction was followed by ^1H NMR for 10 minutes with 30 seconds intervals by monitoring the diagnostic aldehyde ^1H NMR peak at 10.00 ppm. The results were processed with MestReNova Single Rection Monitoring plug-in and converted into concentrations using integration of internal standard with the correction on the initial concentration, and the relative rate was obtained by exponential fit using OriginPro 2017 followed the equation $y = 0.051 + 0.024 \times e^{(-0.347 \times x)}$.

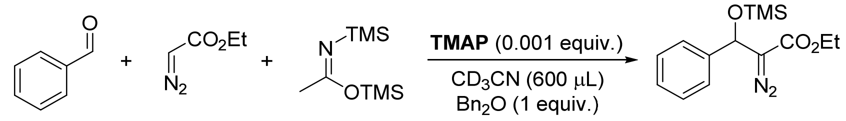

1a (7 mg/mL) **EDA** (0.9 equiv.) **BSA** (2.0 equiv.) **5a**

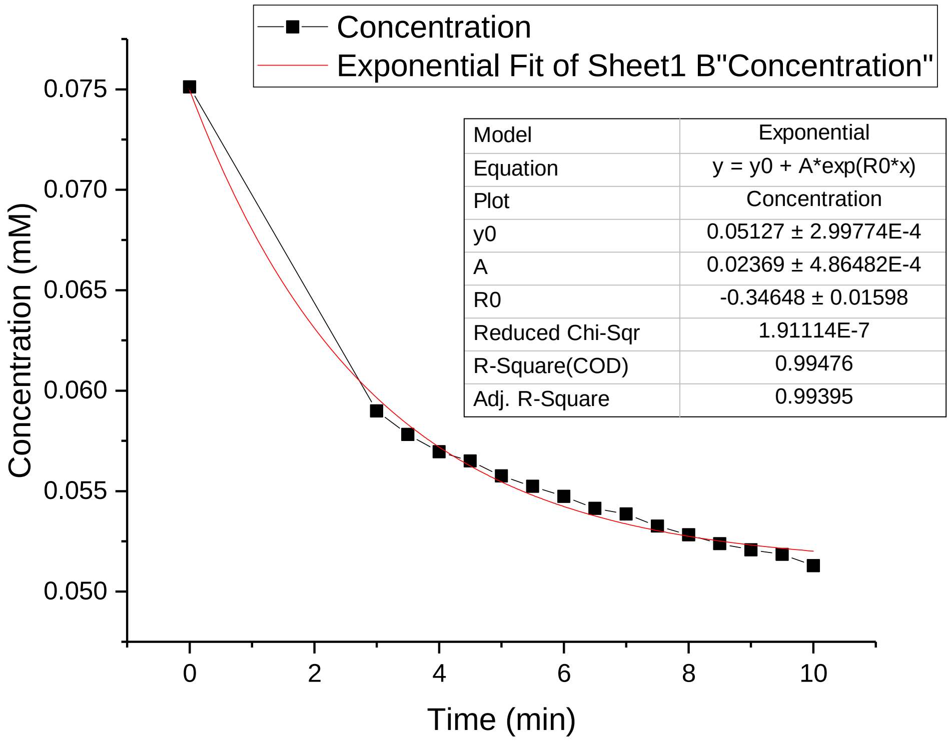

2.3.4 Reaction rate determination with BSTFA (internal code OIC-452)

Benzaldehyde (1.0 equiv, 40 µmol), ethyl diazoacetate (0.9 equiv., 36 µmol), *N,O*-bis(trimethylsilyl)trifluoroacetamide (2.0 equiv., 80 µmol), dibenzyl ether (1 equiv., 40 µmol) were dissolved in CD_3CN (600 µL). The resulting yellow solution was transferred to an NMR tube and the first ^1H NMR spectrum was recorded. The catalyst (TMAP solution, 62 mM in CD_3CN, 0.001 equiv.) was then added, the NMR tube was capped and inverted three times to ensure homogeneity, and the sample was reinserted into the NMR probe. The reaction was followed by ^1H NMR for 10 minutes with 30 seconds intervals by monitoring the diagnostic aldehyde ^1H NMR peak at 10.00 ppm. The results were processed with MestReNova Single Rection Monitoring plug-in and converted into concentrations using integration of internal standard with the correction on the initial concentration, and the relative rate was obtained by exponential fit using OriginPro 2017 followed the equation $y = 0.059 + 0.023 \times e^{(-0.308 \times x)}$.

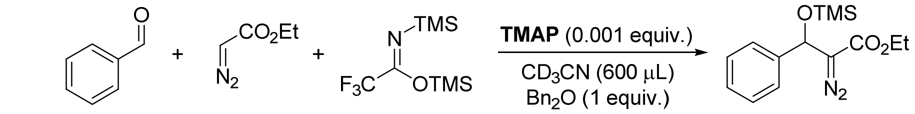

1a (7 mg/mL) **EDA** (0.9 equiv.) **BSTFA** (2.0 equiv.) **5a**

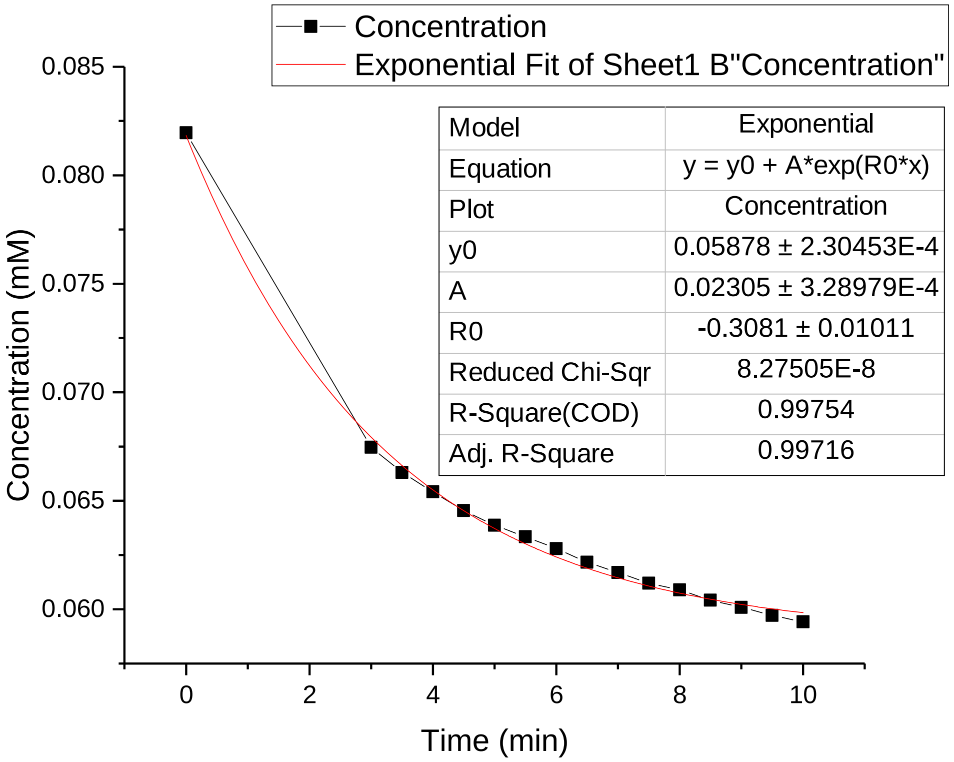

2.4 ^{1}H NMR competition experiments

Procedure: Benzaldehyde (1.0 equiv, 197 μmol), aldehyde (1.0 equiv., 197 μmol), ethyl diazoacetate (0.9 equiv., 182 μmol), *N*,*O*-bis(trimethylsilyl)acetamide (2.0 equiv., 393 μmol), trichloroethylene (1.1 equiv., 222 μmol) were dissolved in CD_3CN (537 μL). The resulting yellow solution was transferred to an NMR tube and the first ^{1}H NMR spectrum was recorded. The catalyst (TMAP solution, 62 mM in CD_3CN, 0.01 equiv.) was then added, the NMR tube was capped and inverted three times to ensure homogeneity, and the sample was reinserted into the NMR probe. The reaction was followed by ^{1}H NMR for 25-30 minutes by monitoring the diagnostic aldehyde ^{1}H NMR peaks at 9.85-10.00 ppm. The results were processed with MestReNova and converted into concentrations using integration of internal standard with the correction on the initial concentration in Microsoft Excel, and the relative rates were obtained by exponential fits using Microsoft Excel.

2.4.1 Competition experiment with **1a** and **1c** (internal code TFN-4-110)

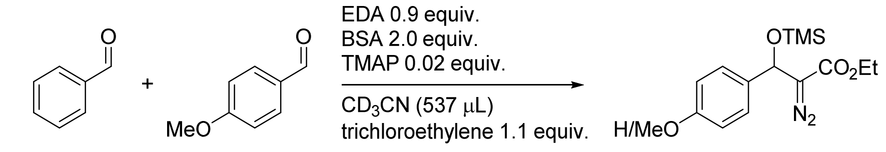

1. First ^{1}H NMR scan of the reaction mixture after 5 min (left zoomed area - C**H**O signals, right zoomed area - ArC**H**OTMSR).

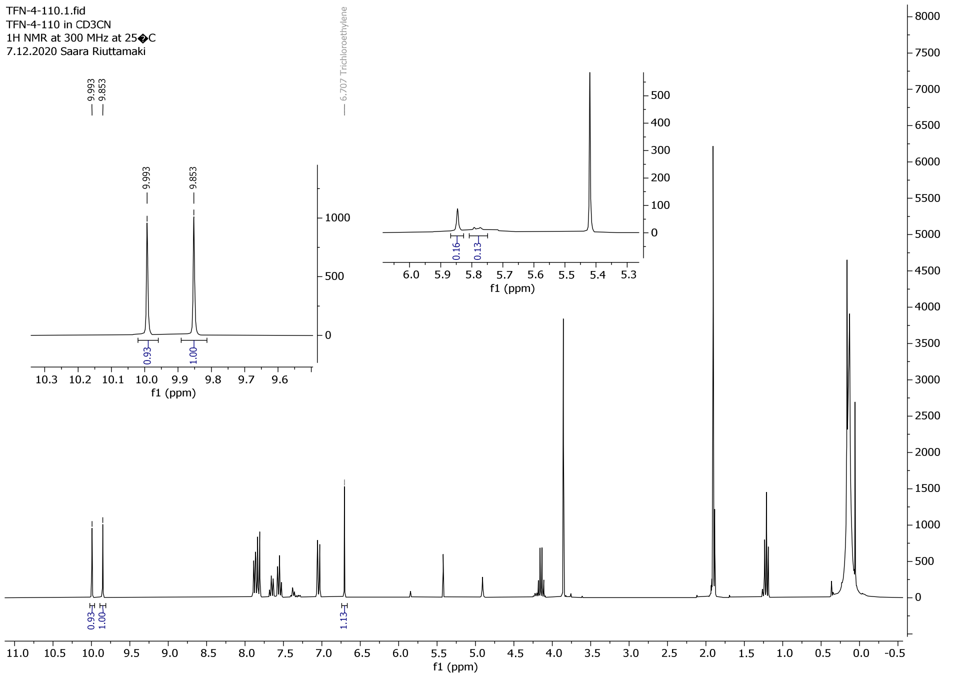

2. Last ^{1}H NMR scan of the reaction mixture after 30 min (left zoomed area - C**H**O signals, right zoomed area - ArC**H**OTMSR).

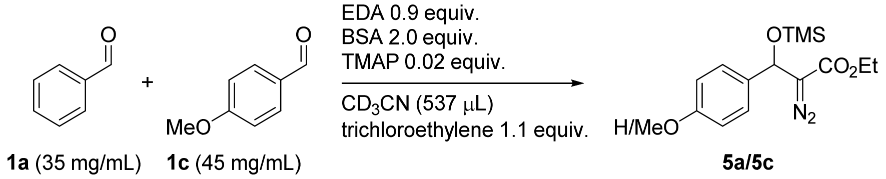

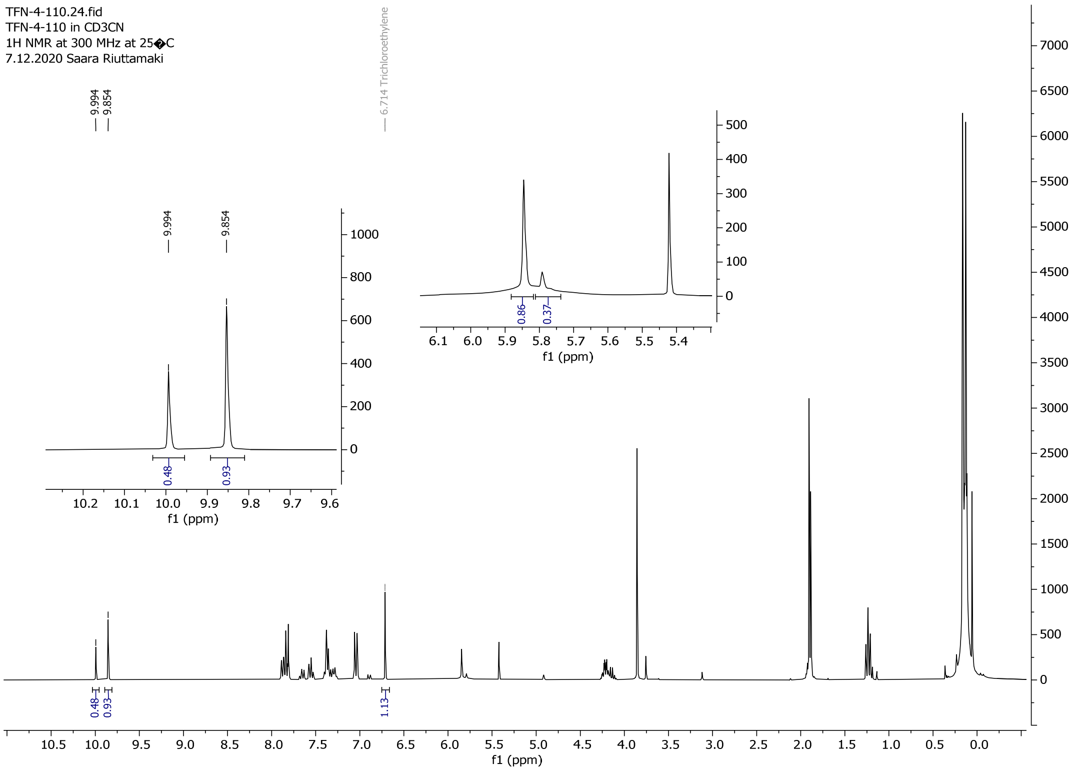

3. Decay curves and exponential fits for aldehyde **1a** (reddish orange spots) and aldehyde **1c** (blue spots). The relative rates can be obtained from the exponentials (in this case, the rate ratio is (–0.003069/–0.0268 = 0.114515).

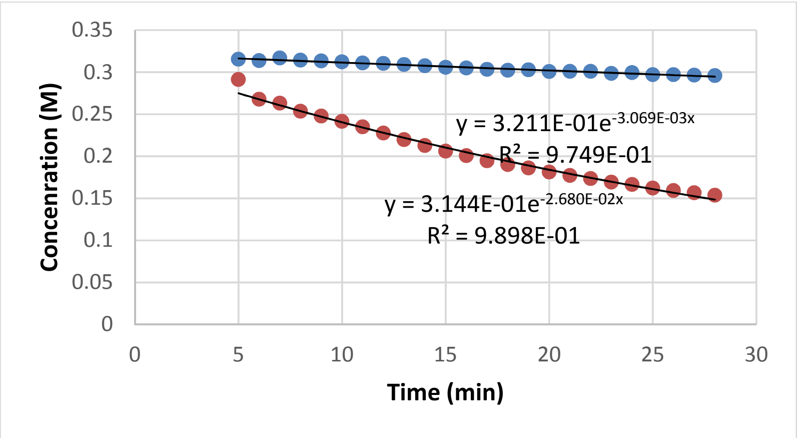

2.4.2 Competition experiment with **1a** and **1d** (internal code TFN-4-112)

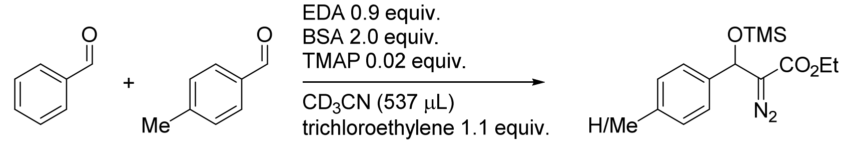

1. First ^{1}H NMR scan of the reaction mixture after 5 min (left zoomed area - C**H**O signals, right zoomed area - ArC**H**OTMSR).

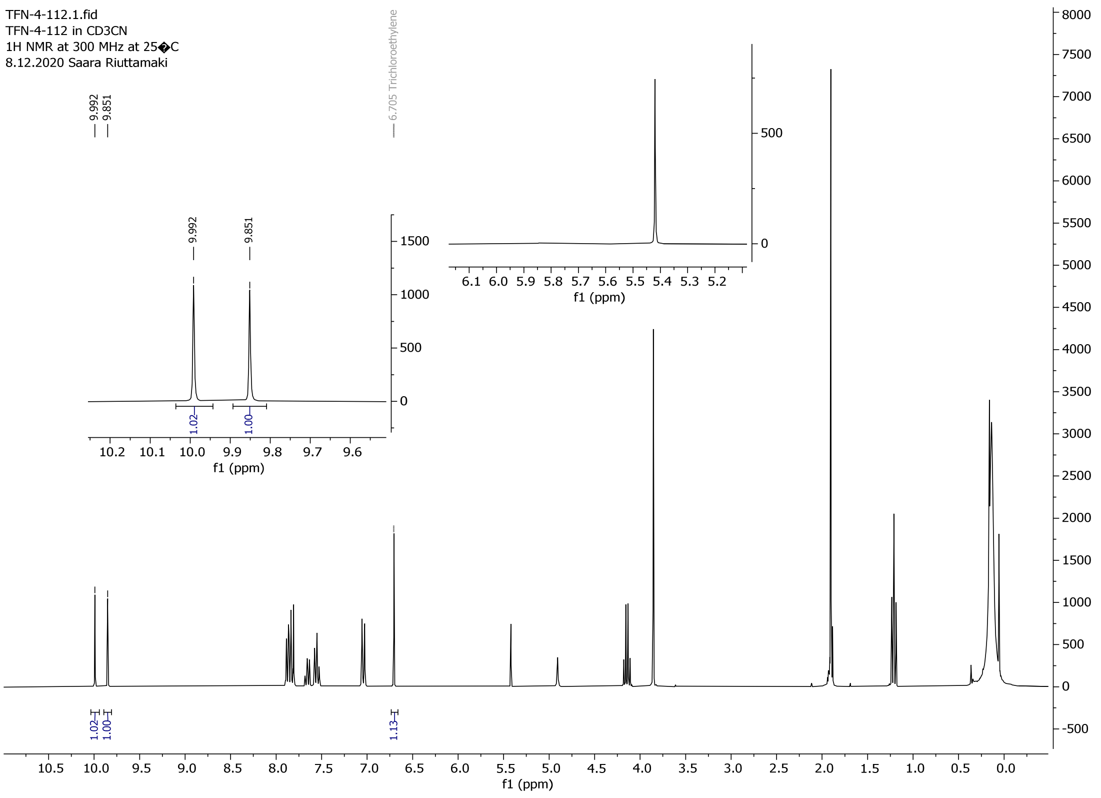

2. Last ^{1}H NMR scan of the reaction mixture after 30 min (left zoomed area - C**H**O signals, right zoomed area - ArC**H**OTMSR).

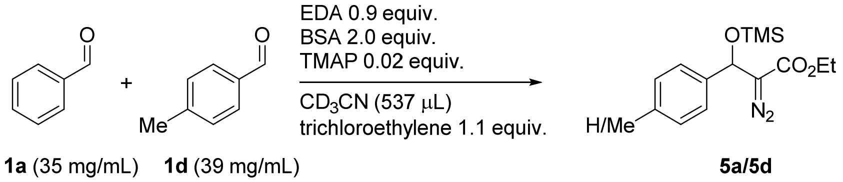

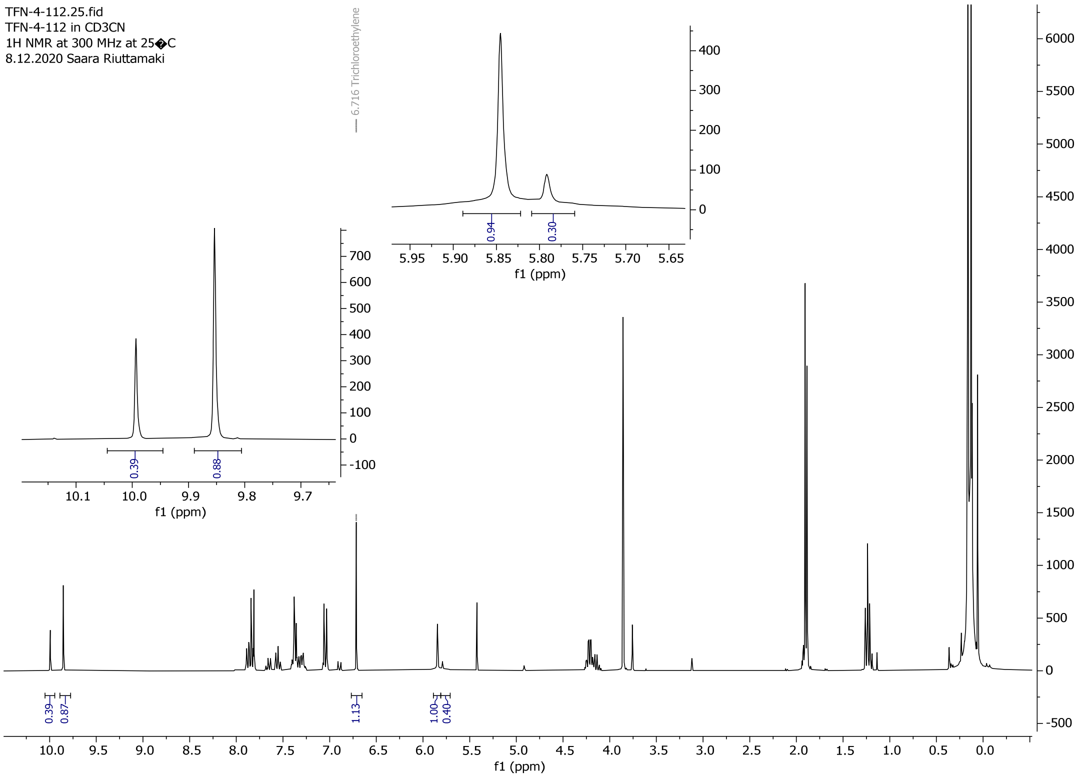

3. Decay curves and exponential fits for aldehyde **1a** (reddish orange spots) and aldehyde **1d** (blue spots).

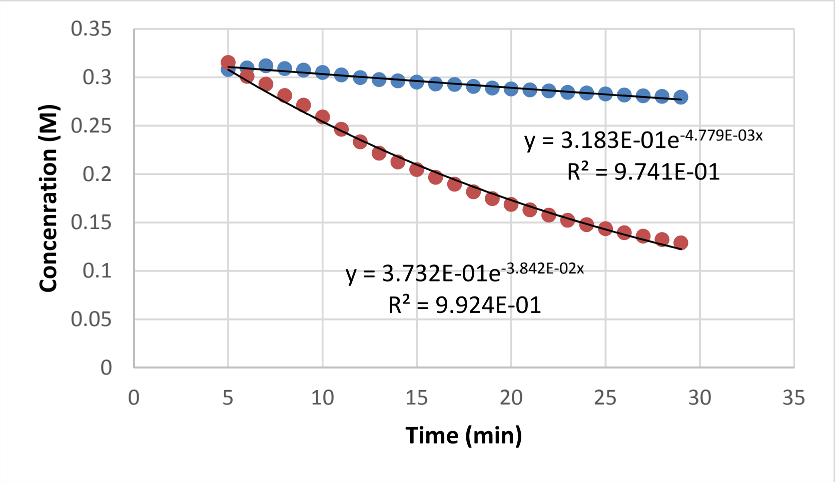

2.4.3 Competition experiment with **1a** and **1f** (internal code TFN-4-114)

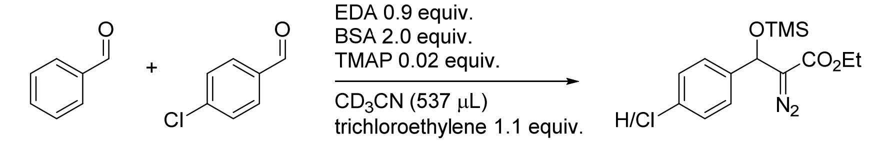

1. First ^{1}H NMR scan of the reaction mixture after 5 min (left zoomed area - C**H**O signals, right zoomed area - ArC**H**OTMSR).

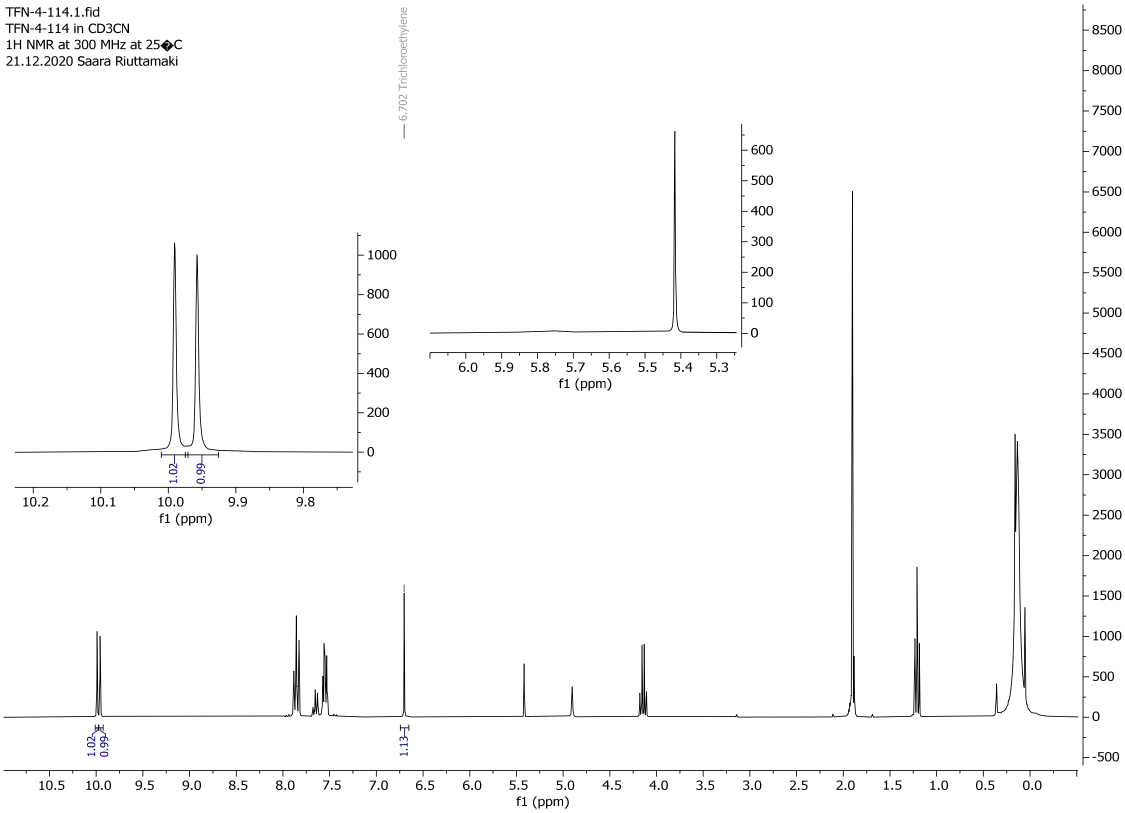

2. Last ^{1}H NMR scan of the reaction mixture after 30 min (left zoomed area - C**H**O signals, right zoomed area - ArC**H**OTMSR).

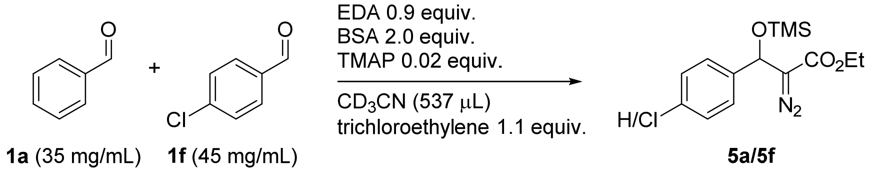

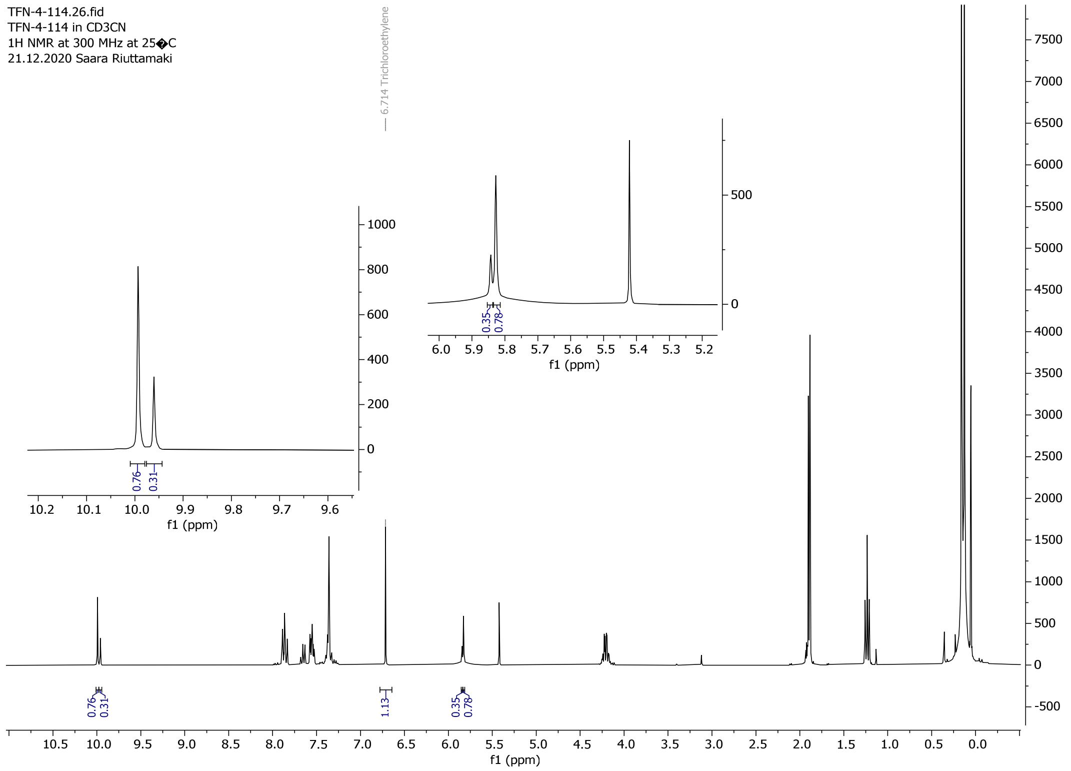

3. Decay curves and exponential fits for aldehyde **1a** (reddish orange spots) and aldehyde **1f** (blue spots).

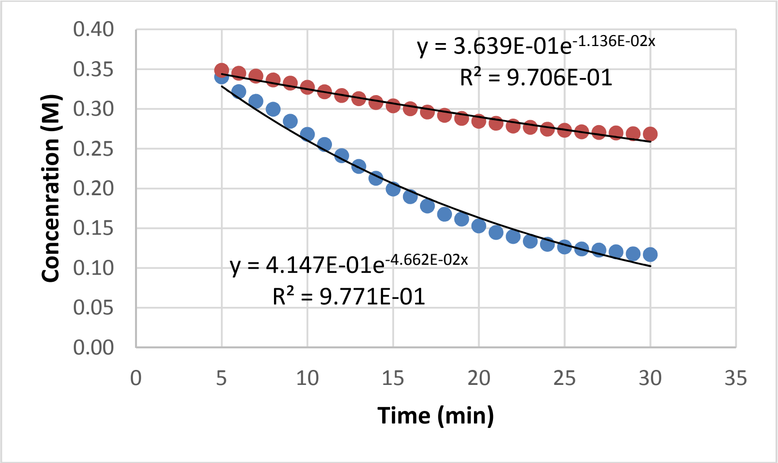

2.5 ^{1}H NMR cross-over experiments

2.5.1 Cross-over experiment with **1b** and **4a** in the presence of BSA (internal code OIC-456)

Procedure: 4-(Trifluoromethyl)benzaldehyde (**1b**) (1.0 equiv, 40 µmol), diazoaldol (**4**) (1.0 equiv., 40 µmol) and dibenzyl ether (1 equiv., 40 µmol) were dissolved in CD_3CN (537 µL). The resulting yellow solution was transferred to an NMR tube and the first ^{1}H NMR spectrum was recorded. *N*,*O*-Bis(trimethylsilyl)acetamide (2.0 equiv., 80 µmol) and the catalyst (TMAP solution, 62 mM in CD_3CN, 0.01 equiv.) were then added subsequently, the NMR tube was capped and inverted three times to ensure homogeneity, and the sample was reinserted into the NMR probe. The reaction was followed by ^{1}H NMR for 20 minutes by monitoring the diagnostic aldehyde ^{1}H NMR peaks at 10.01 and 10.09 ppm respectively. Cross-over reaction equilibrate almost immediately allowed observed only a final ratio between aldehyde **1a** and **1b** using diagnostic peaks at 10.01 and 10.09 ppm respectively. The results were processed with MestReNova Single Rection Monitoring plug-in and converted into concentrations using integration of internal standard with the correction on the initial concentration in Microsoft Excel and plotted in OriginPro 2017.

1. First ^{1}H NMR scan of the reaction mixture without TMAP and BSA (left zoomed area - C**H**O signals, right zoomed area – ArC**H**(OH)R).

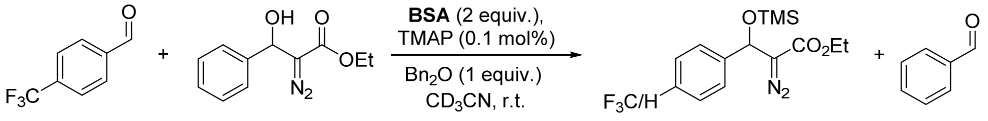

1b **4a** **5a:5b = 83:17** **1a**

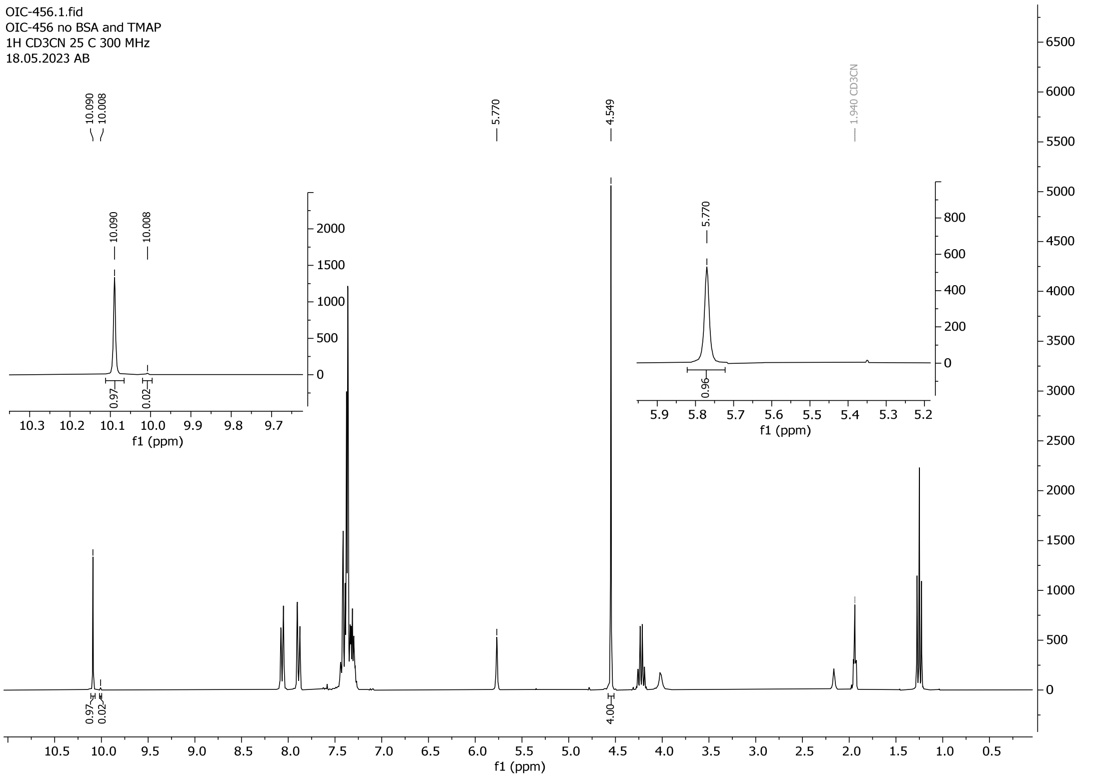

2. Last ^{1}H NMR scan of the reaction mixture with added TMAP and BSA (left zoomed area - C**H**O signals, right zoomed area – ArC**H**(OTMS)R).

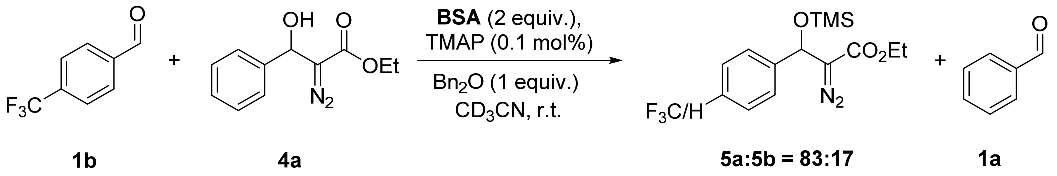

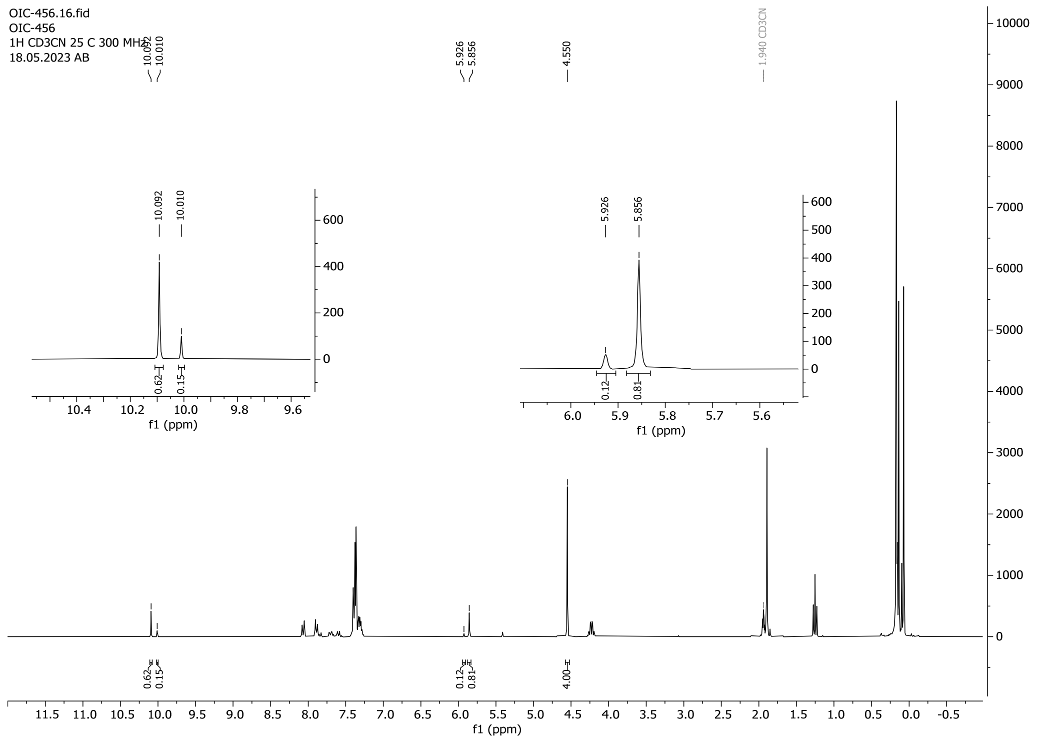

3. Decay plots for aldehyde **1b** (gray spots) and aldehyde **1a** (reddish orange spots).

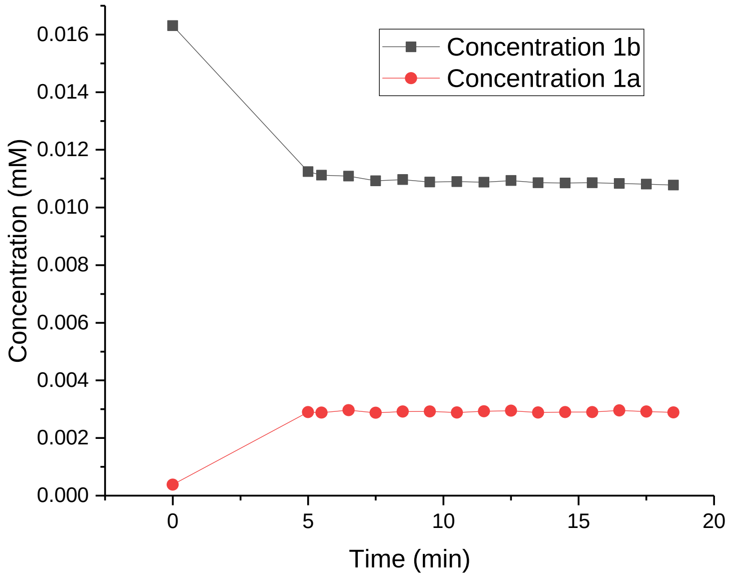

2.5.2 Cross-over experiment with **1b** and **4a** without BSA (internal code OIC-457)

Procedure: 4-(Trifluoromethyl)benzaldehyde (**1b**) (1.0 equiv, 40 µmol), diazoaldol (**4**) (1.0 equiv., 40 µmol) and dibenzyl ether (1 equiv., 40 µmol) were dissolved in CD_3CN (537 µL). The resulting yellow solution was transferred to an NMR tube and the first ^1H NMR spectrum was recorded. The catalyst (TMAP solution, 62 mM in CD_3CN, 0.01 equiv.) was then added, the NMR tube was capped and inverted three times to ensure homogeneity, and the sample was reinserted into the NMR probe. The reaction was followed by ^1H NMR for 20 minutes by monitoring the diagnostic aldehyde ^1H NMR peaks at 10.01 and 10.09 ppm respectively. Cross-over reaction equilibrate almost immediately allowed observed only a final ratio between aldehyde **1a** and **1b** using diagnostic peaks at 10.01 and 10.09 ppm respectively. The results were processed with MestReNova Single Rection Monitoring plug-in and converted into concentrations using integration of internal standard with the correction on the initial concentration in Microsoft Excel and plotted in OriginPro 2017.

1. First ^1H NMR scan of the reaction mixture without TMAP (left zoomed area - C**H**O signals, right zoomed area – ArC**H**(OH)R).

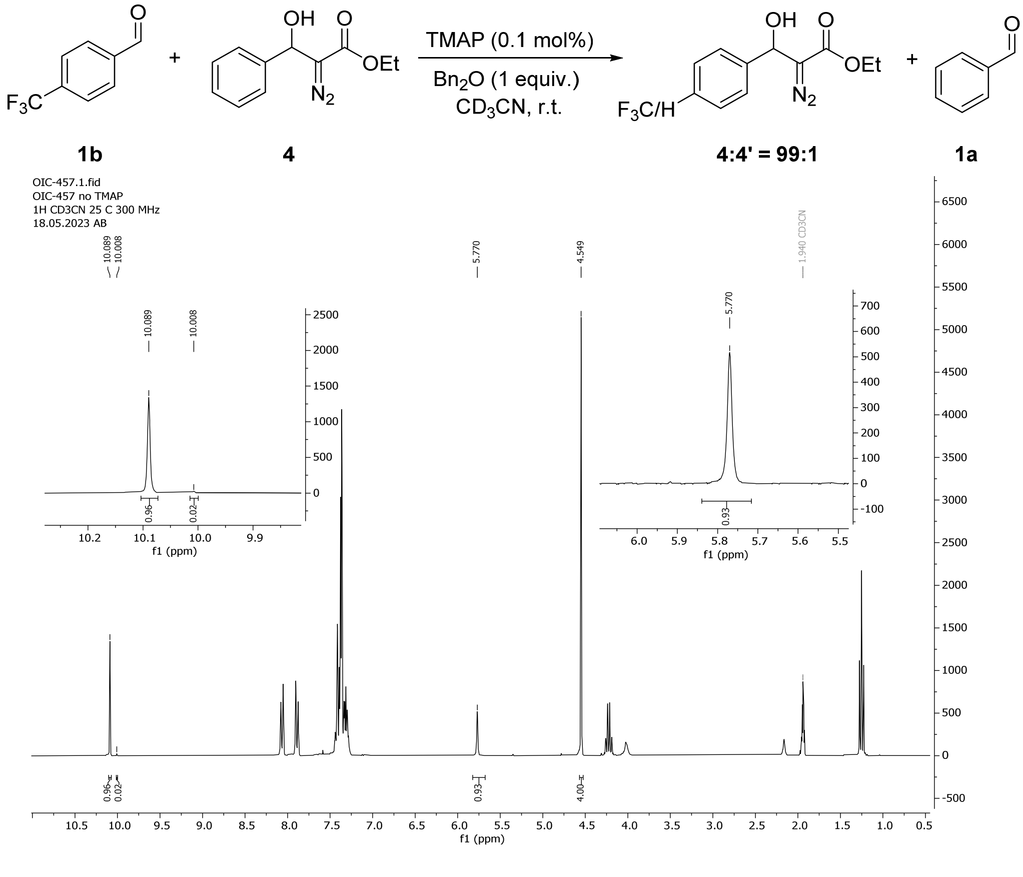

2. Last ^{1}H NMR scan of the reaction mixture with TMAP (left zoomed area - C**H**O signals, right zoomed area – ArC**H**(OH)R).

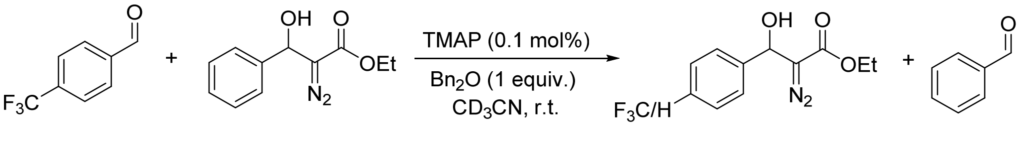

1b **4** **4:4' = 99:1** **1a**

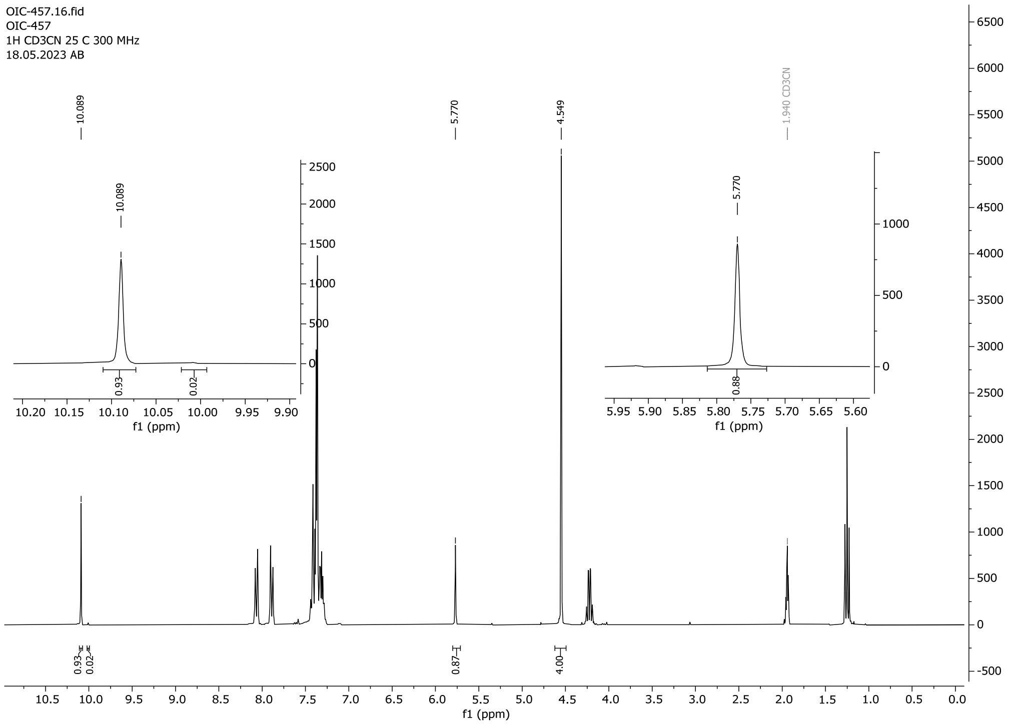

3. Decay plots for aldehyde **1b** (reddish orange spots) and aldehyde **1a** (gray spots).

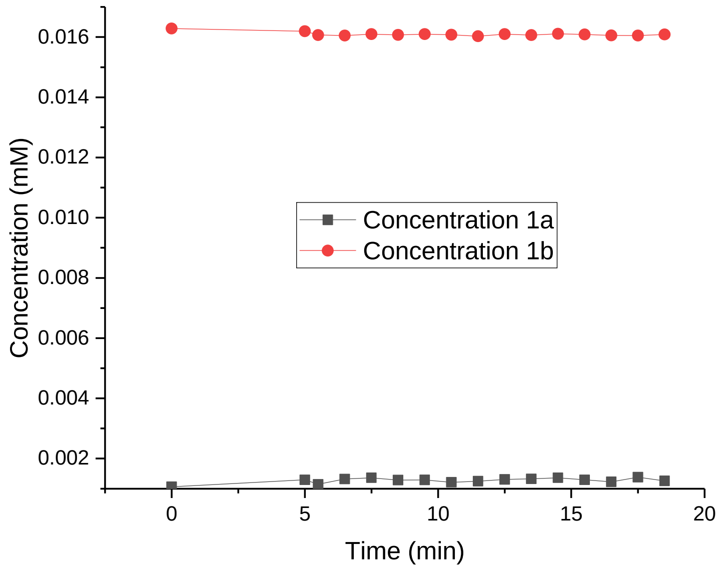

Supplement: Supplementary file 1 — jo3c01304_si_001.pdf [file jo3c01304_si_001.pdf]
