# Supplementary material for: Carboxylate Catalysis: A Catalytic O-Silylative Aldol Reaction of Aldehydes and Ethyl Diazoacetate
Source: J Org Chem. 2023 Sep 28;88(20):14396–403. doi: 10.1021/acs.joc.3c01304 (PMC10594658; doi:10.1021/acs.joc.3c01304)
Supplement: Supplementary file 2 — jo3c01304_si_002.zip [file jo3c01304_si_002.zip › NMR revised/Information.docx]

**Name of the manufacturer of the spectrometer used to collect the data:**

^1^H NMR and ^13^C NMR spectra were recorded on Bruker Avance 500 or 300 MHz spectrometers in CDCl_3_, CD_3_CN or DMSO-*d3.*

**Acquisition software:** Bruker TopSpin 3.5 pl 7 on 500 MHz and Bruker TopSpin 3.5 pl 6 on NMR 300 MHz.

**Processing programs used to analyse the data:** MestReNova 14.3.0

**The field strength used to measure each nucleus:**

**5a**: **^1^H NMR** 300 MHz, **^13^C NMR** 75 MHz

**5b**: **^1^H NMR** 300 MHz; **^13^C NMR** 75 MHz

**5c**: **^1^H NMR** 300 MHz; **^13^C NMR** 75 MHz

**5d**: **^1^H NMR** 300 MHz; **^13^C NMR** 75 MHz

**5e**: **^1^H NMR** 300 MHz; **^13^C NMR** 75 MHz

**5f**: **^1^H NMR** 300 MHz; **^13^C NMR** 75 MHz

**5g**: **^1^H NMR** 300 MHz; **^13^C NMR** 75 MHz

**5h**: **^1^H NMR** 300 MHz; **^13^C NMR** 75 MHz

**5i**: **^1^H NMR** 300 MHz; **^13^C NMR** 75 MHz

**5j**: **^1^H NMR** 300 MHz; **^13^C NMR** 75 MHz

**5k**: **^1^H NMR** 300 MHz; **^13^C NMR** 75 MHz

**5l**: **^1^H NMR** 300 MHz; **^13^C NMR** 75 MHz

**5m**: **^1^H NMR** 300 MHz; **^13^C NMR** 75 MHz

**5n**: **^1^H NMR** 500 MHz; **^13^C NMR** 126 MHz

**6**: **^1^H NMR** 300 MHz; **^13^C NMR** 75 MHz

**7**: **^1^H NMR** 300 MHz; **^13^C NMR** 75 MHz

**8**: **^1^H NMR** 300 MHz; **^13^C NMR** 75 MHz

**9a**: **^1^H NMR** 300 MHz; **^13^C NMR** 75 MHz

**9b**: **^1^H NMR** 500 MHz; **^13^C NMR** 126 MHz

**(±)-5n**: **^1^H NMR** 300 MHz; **^13^C NMR** 75 MHz

**(±)-6:** **^1^H NMR** 300 MHz; **^13^C NMR** 75 MHz
